# Correlation of gene expression and protein production rate - a system wide study

**DOI:** 10.1186/1471-2164-12-616

**Published:** 2011-12-20

**Authors:** Mikko Arvas, Tiina Pakula, Bart Smit, Jari Rautio, Heini Koivistoinen, Paula Jouhten, Erno Lindfors, Marilyn Wiebe, Merja Penttilä, Markku Saloheimo

**Affiliations:** 1 VTT Technical Research Centre of Finland, Tietotie 2, P.O. Box FI-1000, 02044 VTT, Espoo, Finland; 2 NIZO food research, Kernhemseweg 2, 6718ZB Ede, the Netherlands; 3 Plexpress, Viikinkaari 6, 00790 Helsinki, Finland; 4 Ypap Oy, Hyrsynkulmantie 68, FI-32100 Ypäjä, Finland

## Abstract

**Background:**

Growth rate is a major determinant of intracellular function. However its effects can only be properly dissected with technically demanding chemostat cultivations in which it can be controlled. Recent work on *Saccharomyces cerevisiae *chemostat cultivations provided the first analysis on genome wide effects of growth rate. In this work we study the filamentous fungus *Trichoderma reesei *(*Hypocrea jecorina*) that is an industrial protein production host known for its exceptional protein secretion capability. Interestingly, it exhibits a low growth rate protein production phenotype.

**Results:**

We have used transcriptomics and proteomics to study the effect of growth rate and cell density on protein production in chemostat cultivations of *T. reesei*. Use of chemostat allowed control of growth rate and exact estimation of the extracellular specific protein production rate (SPPR). We find that major biosynthetic activities are all negatively correlated with SPPR. We also find that expression of many genes of secreted proteins and secondary metabolism, as well as various lineage specific, mostly unknown genes are positively correlated with SPPR. Finally, we enumerate possible regulators and regulatory mechanisms, arising from the data, for this response.

**Conclusions:**

Based on these results it appears that in low growth rate protein production energy is very efficiently used primarly for protein production. Also, we propose that flux through early glycolysis or the TCA cycle is a more fundamental determining factor than growth rate for low growth rate protein production and we propose a novel eukaryotic response to this i.e. the lineage specific response (LSR).

## Background

Cell growth, i.e. the increase in cell mass per unit of time by macromolecular synthesis, is a major determinant of cell physiology. In the yeast *Saccharomyces cerevisiae *and likely in eukaryotes in general, transcriptome, proteome and metabolome are greatly influenced by the growth rate [1,2]. The small genome of *S. cerevisiae *[3] and its recent genome duplication [4] make its genome exceptional among fungi [5]. In addition, it is a single cell organism capable of anaerobic growth. In *S. cerevisiae *expression of protein synthesis, essential and conserved genes is positively correlated with growth rate, while genes related to signalling, external stimuli and communication have a negative correlation [1].

In general the transcript levels of genes are regulated through interplay of transcription factors, chromatin modifications and RNA degradation rate. The TOR (Target of Rapamycin) network links intra- and extra cellular signals to control the growth rate of *S. cerevisiae*. It regulates gene expression through a variety of transcription factors [1,6]. In parallel, the *SNF1 *network is a central regulator of carbon metabolism [6,7]. The yeast *SNF1 *protein kinase complex is composed of *α *(*SNF1*), *β *(*GAL83*, *SIP1 *or *SIP2*) and *γ *(*SNF4*) subunits. In particular, it induces glucose repressed genes by phosphorylating and hence inactivating a repressing transcription factor, *MIG1*, and activating other inducing transcription factors such as *ADR1 *and *CAT8*. The *TOR1 *and *SNF1 *networks are likely to regulate for example amino acid, energy and lipid metabolic pathways in concert [7], integrating signals of nutritional and metabolic state. Histone acetylation at promoters by histone acetyl transferases (HATs), such as *S. cerevisiae GCN5 *and *ESA1*, or methylation across transcribed sequence are generally associated with active transcription of genes [8]. In particular, the SWI/SNF complex acts as a chromatin remodelling complex for glucose regulated genes under the control of *SNF1 *and enables their transcription in concert with HATs [9]. Fungal genomes are a mosaic of chromosomal regions (or whole supernumary chromosomes [10,11]), where gene content and order is mostly conserved between closely related species (syntenic blocks) and regions where it is not conserved (non-syntenic blocks). In Pezizomycotina non-syntenic blocks may be enriched in orphan genes [10,12,13] and in specific protein families [14,15], that are typical to Pezizomycotina, such as plant biomass degradation and secondary metabolism related proteins [5]. Alternatively, the distribution of orphan genes across a fungal genome can be uniform [16]. Genes in non-syntenic blocks can be particularly short [15]. Non-syntenic blocks are often found near telomers [15,17], where recombination rates can be high [10,16,18] and secreted, orphan [19] and paralogous genes [16] and single nucleotide polymorphisms [10] may be enriched.

Starvation-like conditions can cause general induction of genes in non-syntenic blocks [20,21]. Similarly, carbon limitation and in particular lack of glucose can induce or derepress plant biomass degradation and secondary metabolism related genes in filamentous fungi. In *S. cerevisiae*, which mostly lacks above mentioned functions, carbon limitation induces only genes related to metabolism of storage carbohydrates and use of alternative carbon sources [2,6]. In relation to regulation of gene expression, it is of note that by gene count the 'DNA binding N-terminal zinc binuclear cluster' (Zn2Cys6) (for review [22]) transcription factor family is one of the most variable and abundant protein families in Ascomycota [5]. On average a Pezizomycotina species has three times more of Zn2Cys6 genes than a Saccharomycotina species. These often reside beside secondary metabolism genes clusters in fungi [23] and more generally in non-syntenic blocks in *Trichoderma reesei *[14] and thus are prime candidates as direct regulators of non-syntenic block genes. However, secondary metabolism gene clusters can be directly activated by manipulation of histone methylation [24] or histone deacetylation related genes [25] as well as induction of a cluster's Zn2Cys6 transcription factor [26]. Furthermore, the order and timing of transcriptional activation of a secondary metabolism cluster might be determined by histone acetylation [27].

The Pezizomycotina *T. reesei *(teleomorph *Hypocrea jecorina*) is a known producer of native cellulase and hemicellulase enzymes, but also of recombinant proteins. *T. reesei *is an important model organism of lignocellulosic biomass degradation and it can, remarkably, produce over 100*g = l *yields of extracellular protein in industrial cultivations [28]. In chemostat cultivations the highest specific extracellulaer protein production rates for *T.reesei *have been detected at a relatively low specific growth rate of *D *= 0.03 i.e. it exhibits a low growth rate protein production phenotype [29-32]. This phenotype has been described in other Sordariomycetes [33], while high growth rate protein production has been described in Eurotiomycetes [34,35].

In *T. reesei *both inducing and repressing regulators of cellulase gene expression are known. *cre1 *[36] is the orthologue of *S. cerevisiae MIG1 *i.e. the transcription factor responsible for carbon catabolite repression.

This repression ensures that in presence of D-glucose, or other monosaccharides whose catabolism provides a high yield of ATP, no energy is wasted in production of cellulases. Suprisingly, for *T. reesei*, lactose is a carbon source that induces cellulase expression (for review [37,38]). Soluble lactose is far easier to handle in liquid cultivations than the natural inducing carbon sources e.g. cellulose.

To study the effects of growth rate or to expilicitly exclude the effect of growth rate from a study one must be able to control it precisely. A chemostat is a bioreactor cultivation in which some substrate component such as the main carbon source, e.g. lactose, limits biomass production and is fed at a constant rate which determines the specific growth rate of the organism. In addition, use of bioreactors instead of flask cultivations allows for a very fine control of growth conditions and hence more reliable and comparable measurements [39,40].

In order to study the intracellular effects of the low growth rate protein production phenotype we carried out transcriptomic and proteomic profiling on chemostat cultivations. We find a strong co-regulation and induction of genes related to secondary metabolism and of secreted proteins, and a general down regulation of major cellular systems of primary metabolism, protein synthesis and secretion in condition of high cellulase production. Our results suggest the existance of eukaryotic response to low flux through early glycolysis or TCA cycle in the form of induction of lineage specific genes.

## Results

### Chemostat cultivations

In order to study the correlation of gene and protein expression with specific extracellular protein production rate (SPPR) we grew *T. reesei *in lactose limited chemostats in three conditions: specific constant growth rates of 0.03 h^-1 ^(D03) and 0.06 h^-1 ^(D06) with 10 g/L of lactose and 0.03 h^-1 ^with 40 g/L lactose for higher cell density (HD). Triplicate cultivations were analysed for the three conditions. Based on [32], the highest specific extracellular protein production rate was expected in D03 and the lowest in D06 cultivations. HD cultivations enable us to try to separate growth rate effects from specific extracellular protein production rate effects and provide valuable data from high density conditions often used in the protein production industry.

**Figure 1:**
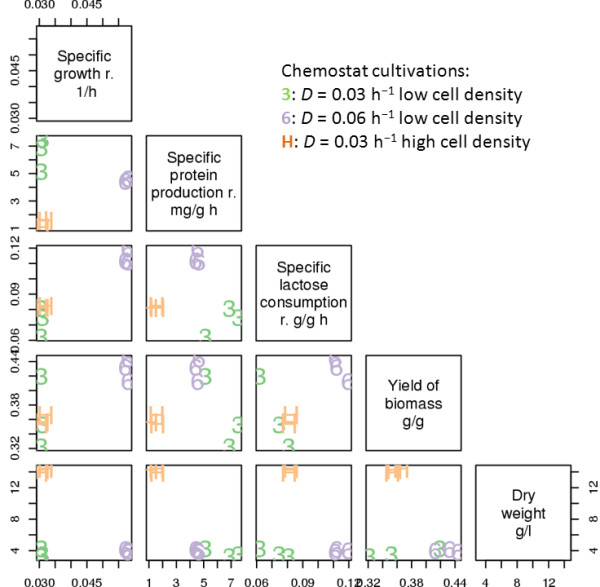
**Scatterplot of cultivation parameters**. Diagonal panels contain axes labels. For each scatterplot the × axis label is found on the diagonal panel above the plot and for the Y axis on the right side. The chemostat cultivations are coded as '3' = *D *= 0.03 h^-1 ^low cell density (D03), '6' = *D *= 0.06 h^-1 ^low cell density (D06) and 'H' = *D *= 0.03 h^-1 ^high cell density (HD).

Scatterplots of cultivation parameters are shown in Figure 1, and all the parameters are shown in Additional file 1, Table S1. The specific extracellular cellulase production rates and the yield of extracellular protein correlated strongly with SPPR and the specific sulphate consumption rate with specific lactose consumption rate and hence, are not shown in Figure 1.

As expected the highest SPPR, and the accordingly highest specific cellulase production rate was observed in D03 cultivations along with the lowest lactose consumption rate. D06 cultivations had the highest specific lactose consumption rate and on average the highest yield of biomass. HD cultivations had the highest dry weight, the lowest specific extracellular protein production rate and on average 0.07 lower yield of biomass than in D06 cultivations (*p <*0.005 in Student's t-test). Hence, in HD cultivations both the production of biomass and protein was inefficient. Since in the HD cultivations the lactose concentration fed to the cultivation was 4 times higher than in D03 cultivations, accordingly the dry weight was 3.9 times higher. Residual lactose was not detected in any of the cultivations.

One of the D03 cultivations, F20, differed from the other two D03 cultivations (Figure 1). Although, F20 had higher protein production rate than any D06 or HD cultivation, the yield of biomass was in the range of D06 cultivations, while its specific lactose consumption rate was lower than that of other D03 cultivations.

### Transcriptome analysis

We used oligonucleotide microarray transcriptome profiling to analyse the nine chemostat cultivations. A general workflow of the analysis is presented in Additional file 1, Figure S1. Nine genes relevant for protein production were previously analysed in a separate study with Northern hybridisations in conditions similar to *D *= 0.03 h^-1 ^low cell density (D03) and *D *= 0.06 h^-1 ^low cell density (D06) [32]. In our transcriptome profiling data the direction of change for all of these genes, except *hac1 *(encoding the unfolded protein response transcription factor), was the same. However, we found that the gene model used for design of *hac1 *microarray probes was incorrect and moreover *hac1 *has splice variation [41] that is not measured by our microarray.

In addition, we verified our microarray analysis with re-analysis of expression of 31 genes from the same nine samples with the TRAC method [42]. In order to correlate TRAC and microarray data we tested various models and a model that best explained the data based on Akaike information criteria was selected. We estimated correlation of *r*^2 ^= 0.986 with a p-value *<*2.2*e *- 16 between TRAC and microarray data. The model fits a gene specific intercept i.e. it shows that the basal signal values of each gene between TRAC and microarray signal differ (Additional file 1, Figure S2). However, some heteroscedasticity, i.e. non-consistency of variation, can be seen in the 'Residuals vs Fitted' panel of Additional file 1, Figure S2. We then looked for genes with significant change in expression between the three conditions with false discovery rate of 5%. We found that the largest amount of changing genes was detected between *D *= 0.03 h^-1 ^low cell density condition (D03) and *D *= 0.03 h^-1 ^high cell density condition (HD) (203 higher in D03 and 736 higher in HD), while *D *= 0.06 h^-1 ^low cell density condition (D06) versus *D *= 0.03 h^-1 ^high cell density condition (HD) had less differencies (97 and 375, respectively) and D03 versus D06 only minor differences (10 and 30, respectively) (Additional file 1, Table S2). Genes at a higher expression level in D03 than D06 included four glycoside hydrolase (endomannanase *man1*, endoglucanase *egl3*, alpha-xylosidase and a putative xylanase) and three transporters of the MFS family. *cbh1 *and *egl1 *genes were detected to have a higher expression level in D03 cultivations than in D06 cultivations by microarray, TRAC and previously by Northern [32], but their differential expression was not statistically significant with the cut-offs used, possibly due to microarray signal saturation. The annotation category that was enriched among the genes at higher expression level in D06 than in D03 was mitochondrial genes (Additional file 1, Table S3). No function could be determined for 30% of the genes at higher expression level in D06 than in D03. Among the genes at higher expression level in D06 than in HD there was enrichment in genes of unknown function and genes related to secondary metabolism. In addition, GCN5-related N-acetyltransferases were enriched. All categories that were enriched among genes at higher expression level in HD than in D06 included less than 2% of these genes i.e. the enriched categories do not reveal general trends among these genes. Among genes at higher expression level in D03 than in HD, genes of unknown function, secreted proteins, secondary metabolism related and transporters were enriched. Among genes at higher expression level in HD than in D03, protein synthesis and energy related genes were particulary enriched. Notably, genes of the pentose phosphate pathway, TCA cycle and threonine metabolism were enriched in this gene set.

Results of the chemostats showed that each of our conditions had its own distinct SPPR which enabled a correlation between SPPR and each gene's expression values to be calculated. However, as already noted F20 was slightly exceptional among the *D *= 0.03 h^-1 ^low cell density (D03) cultivations. In a standard analysis of significant change of gene expression between conditions, as presented above, such an outlier could seriously affect the results, if the differences in its cultivation parameters would be reflected on its transcriptome. In contrast, in an analysis of correlation of SPPR and each gene's expression, F20 is just one data point on the regression line correctly positioned by its SPPR. As F20 is not considered as a repeat of other D03 cultivations, but as an independent data point, F20 is no longer considered as an outlier. Thus, for each gene we calculated its correlation with SPPR and after correction for multiple testing, set a cut-off of absolute correlation 0.8, which corresponds to a false discovery rate of 3.3%. 490 genes were found to have a negative correlation and 477 a positive correlation with SPPR (Additional file 1, Table S2). If F20 is excluded from the data set the correlation of genes to SPPR remains essentially the same, but the count of genes with absolute correlation above 0.8 increases by 40% (Additional file 1, Figure S3). Hence, we retained F20 in the data set in order to present a more conservative analysis.

Given the non-random structure of fungal genomes, we estimated that the probability of detecting a pair of adjacent genes on a chromosome which would both have absolute correlation *>*0.7 to SPPR and the same direction of correlation, was *p <*1*e *- 5. 187 genes were found to belong to such a pair with negative correlation and 224 with positive correlation. However, the above p-value does not take into account that the co-regulation of the gene pair might be due to a shared promoter. Among gene pairs positively correlated to SPPR, 24 pairs were arranged so that they could share a promoter and of these in 10 pairs the beginnings of genes were separated with less than 1000 bases. Among negatively correlated pairs the respective numbers were 17 and 13 pairs. Hence, the effect of promoter sharing is not large.

#### General characteristics of genes with differential correlations with SPPR

Given the characteristic differences of fungal genes in syntenic blocks and non-syntenic blocks, we analysed the genes with significant positive (477) or negative (490 genes) correlation with SPPR for their maximum expression value, absolute fold change, distance to scaffold end, length and GC% (Table 1 Additional file 1, Figure S4). We found that genes correlating positively with SPPR have on average a maximum expression value which was clearly lower than that of genes correlating negatively with SPPR. Similary, genes correlating positively tend to be situated nearer to scaffold ends than negatively correlated, have lower fold change than negatively correlated and have a lower GC%. No evidence was found for difference in GC% of negatively correlated genes and non correlated genes or for differences in gene length of genes correlating from non correlating genes. We then verified that the same trends also applied for pairs of adjacent genes correlating with SPPR (Additional file 1, Figure S4). Notably, the GC% of pairs of adjacent correlating genes with a correlation between 0.7 - 0.8, was even lower (55.6, *p <*1.7*e *- 09) than genes with a correlation above 0.8. Furthermore, we verified that maximum expression value does not explain the correlation between gene expression and SPPR when considering all genes (Additional file 1, Figure S5).

**Table 1:** General characteristics of genes with differential correlations to SPPR

General gene characteristic	Non-correlated genes	Genes with significant negative correlation with SPPR (-C08)	p-value of difference of -C08 to non-correlated	Genes with significant positive correlation with SPPR (C08)	p-value of difference of C08 to non-correlated
Maximum expression	8.8	10.0	0.0001	8.4	2E-16
Log2 fold change	1.2	1.6	2E-16	1.3	3.6E-15
GC percentage	58.2	58.5	-	57.5	0.001
Distance to scaffold end	194603.8	220703.5	0.05	154861.6	0.0006
Length	1306.5	1271.9	-	1357.9	-

For each gene characteristic an average value in three gene groups (non-correlated, negatively correlated and positively correlated) and a p-value for the difference of the average of a correlated group versus the non-correlated group is given.

#### Distribution of correlation of gene expression to SPPR

**Figure 2:**
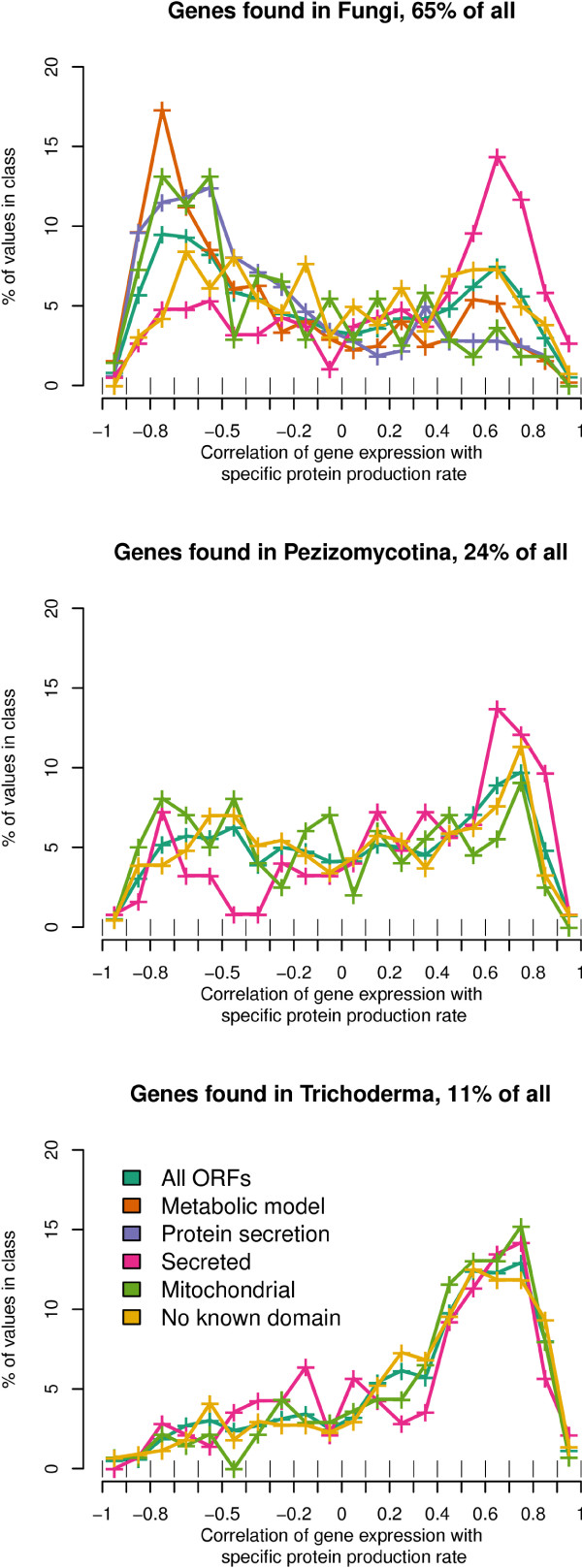
**Distribution of correlation of gene expression with SPPR**. Genes of *T. reesei *genome are divided in three plots based on their taxonomic specificity, either found in general in Fungi, only in Pezizomycotina and only in Trichoderma. Each plot shows a distribution for all genes in it and in four different mutually exclusive cellular categories. The Y axis shows the percentage of genes of all genes in the category contained in each bin. The × axis shows the correlation and borders of bins. Plus signs indicate mid values of bins and lines connect them. Cellular categories with at least 50 genes are shown.

In order to understand how the correlation of gene expression to SPPR varied in different functional categories, *T. reesei *genes were mapped to five broad, mutually exclusive cellular categories: genes of the *T. reesei *metabolic model, genes involved in protein secretion, genes of secreted proteins, mitochondrial genes and genes with no known domain i.e. unknown genes (Figure 2). For example, the genes of secreted proteins are genes predicted to be secreted, but not included in the secretion pathway or any other of the categories. Furthermore, we divided all genes of the genome in three categories of lineage specificity based on whether they are found in sequenced Fungi in general, Pezizomycotina only or only in the genus Trichoderma (i.e. Hypocrea). Overall, a bimodial distribution was detected. Genes of protein secretion and the metabolic model had a negative correlation, while unknown genes and genes of secreted proteins tended to have a positive correlation with SPPR. For protein secretion, metabolic model and unknown genes, this closely parallels their lineage specificity i.e. genes found in Fungi tended to have negative correlation while those limited to Trichoderma tended to have a positive correlation to SPPR.

Mitochondrial genes are divided into two groups, depending on whether they are known mitochondrial genes found in Fungi (negative correlation with SPPR) or genes found in Trichoderma with little other information than the predicted location (positive correlation with SPPR). Of mitochondrial genes with correlation to SPPR above 0.6 only 31% have a known domain.

Genes of secreted proteins had a positive correlation to SPPR regardless of their lineage specificity. Of genes of secreted proteins, with correlation to SPPR above 0.6, 61% of those found in Fungi, 9% of those only in Pezizomycotina and 4% of those only in Trichoderma were CAZymes.

Overall 19% of all glycoside hydrolases were found to have a positive correlation above 0.7 with SPPR and 12% of them a correlation below -0.7. Of multi gene glycoside hydrolase families in *T.reesei*, families 1 (beta-glucosidases) and 37 (trehalases) had an average correlation to SPPR below -0.6 and 89 (alpha-N-acetylglucosaminidases), 75 (chitosanase), 11 (xylanases) and 79 (beta-glucuronidase) above 0.6. Given these trends, exceptions to them are of special interest. Of the 22 genes in metabolic model with a correlation to SPPR above 0.7, 9 are possibly related to secondary metabolism and one is a chitin synthase. Eight have a broad substrate range making their functional assingment difficult. Four genes with orthologues in *S. cerevisiae *do not belong to the above mentioned categories. Of these *GLY1*, *HIS6 *and *DAL1 *are related to amino acid metabolism and *PNC1 *is a longevity gene [43].

In protein secretion 16 genes have a correlation to SPPR above 0.7. 9 are related to glycosylation including *dpm2 *[44] and orthologues of *S. cerevisiae DIE2*, *ALG12 *and *YEA4*. Other genes with orthologues of *S. cerevisiae *include *ERO1*, *SEC6*, and *pdi1 *[45].

#### Enrichment of functional categories in genes with significant correlation to SPPR

To further understand the correlations of genes to SPPR we carried out enrichment tests for genes with correlation above 0.8 ('C08') or below -0.8 ('-C08') and respectively for adjacent gene pairs of absolute correlation above 0.7 and of the same direction of correlation ('C07wN', '-C07wN', Table 2). Although sets of genes changing significantly between the three conditions, discussed above, never overlap with genes with significant correlation to SPPR by more than 57%, the functional categories are very similar in both gene sets. For 'C08' secreted, transporters and secondary metabolism related categories are enriched. In contrast in 'C07wN' no transporters are enriched, but protein degradation related genes are enriched. In '-C08' protein synthesis, cytoskeleton and protein degradation related genes are mainly detected. In contrast in '-C07wN' no protein degradation or cytoskeleton related genes are enriched, but more regulation related genes are enriched. Of genes related to protein degradation, proteasome components are enriched among genes with negative correlations while peptidases are enriched among genes with positive correlations.

**Table 2:** Enriched functional categories of genes correlating with specific extracellular protein production rate

ID	Description	Expression	% of ex-pressed	% of annotated	p-value	Intepretation
IPR000639	Epoxide hydrolase-like	C07wN	2.2	26.7	0.000	Various
IPR006163	Phosphopantetheine-binding	C07wN	2.7	18.5	0.000	2ndary metabolism?
IPR000873	AMP-dependent synthetase/ligase	C07wN	2.7	12.8	0.001	2ndary metabolism?
IPR001579	Glycoside hydrolase, chitinase active site	C07wN	1.6	27.3	0.001	Secreted
IPR002938	Monooxygenase, FAD-binding	C07wN	1.6	23.1	0.002	2ndary metabolism?
IPR000209	Peptidase S8/S53, subtilisin/kexin/sedolisin	C07wN	2.2	14.8	0.003	Protein degradation
IPR001223	Glycoside hydrolase, family 18, catalytic domain	C07wN	1.6	18.8	0.005	Secreted
IPR000073	Alpha/beta hydrolase fold-1	C07wN	2.2	10.3	0.010	2ndary metabolism?
IPR011701	Major facilitator superfamily MFS-1	C07wN	4.4	5.3	0.017	Transporter
IPR003663	Sugar/inositol transporter	C07wN	2.2	8.3	0.020	Transporter
IPR000217	Tubulin	-C07wN	1.6	60.0	0.000	Cytoskeleton
IPR001353	Proteasome, subunit alpha/beta	-C07wN	2.2	28.6	0.000	Protein degradation
IPR000504	RNA recognition motif, RNP-1	-C07wN	2.7	8.8	0.008	Protein synthesis
IPR000719	Protein kinase, catalytic domain	-C07wN	3.8	5.8	0.016	Regulation
12.04	translation	-C07wN	3.6	14.0	0.017	Protein synthesis
12.04.01	translation initiation	-C07wN	2.3	18.5	0.018	Protein synthesis
IPR000623	Shikimate kinase	C08	0.7	60.0	0.001	Various
IPR011701	Major facilitator superfamily MFS-1	C08	3.9	10.5	0.003	Transporter
IPR003819	Taurine catabolism dioxygenase TauD/TfdA	C08	0.7	42.9	0.003	Sulphur metabolism
IPR006094	FAD linked oxidase, N-terminal	C08	1.2	19.2	0.007	2ndary metabolism?
IPR003663	Sugar/inositol transporter	C08	1.7	14.6	0.008	Transporter
IPR006163	Phosphopantetheine-binding	C08	1.2	18.5	0.009	2ndary metabolism?
IPR002403	Cytochrome P450, E-class, group IV	C08	1.0	22.2	0.010	2ndary metabolism?
IPR004841	Amino acid permease domain	C08	1.2	15.6	0.018	Transporter
IPR000254	Cellulose-binding domain, fungal	C08	0.7	23.1	0.023	Secreted
IPR002018	Carboxylesterase, type B	C08	0.7	21.4	0.028	Secreted
IPR002085	Alcohol dehydrogenase superfamily, zinc-containing	C08	1.5	11.8	0.036	2ndary metabolism?
IPR001452	Src homology-3 domain	-C08	1.6	28.0	0.000	Cytoskeleton
IPR001353	Proteasome, subunit alpha/beta	-C08	1.1	35.7	0.001	Protein degradation
IPR002423	Chaperonin Cpn60/TCP-1	-C08	0.9	40.0	0.001	Cytoskeleton
IPR002108	Actin-binding, cofilin/tropomyosin type	-C08	0.7	60.0	0.001	Cytoskeleton
IPR005937	26S proteasome subunit P45	-C08	0.7	50.0	0.003	Protein degradation
IPR000717	Proteasome component (PCI) domain	-C08	0.9	30.8	0.004	Protein degradation
IPR004827	Basic-leucine zipper (bZIP) transcription factor	-C08	1.1	22.7	0.005	Regulation
IPR000594	UBA/THIF-type NAD/FAD binding fold	-C08	0.7	37.5	0.007	Protein degradation
12.04	translation	-C08	2.9	24.6	0.012	Protein synthesis
12.04.01	translation initiation	-C08	1.6	29.6	0.017	Protein synthesis
IPR001199	Cytochrome b5	-C08	0.9	19.0	0.024	Energy
IPR003593	ATPase, AAA+ type, core	-C08	2.2	9.8	0.046	Various

'ID' is InterPro or Funcat identifier. 'Description' is short name for the category. 'Expression' is type of expression behaviour the genes have. 'C07wN' genes belong to a pair of adjacent genes that both have correlation to specific protein production rate (SPPR) above 0.7, '-C07wN' is the opposite case, 'C08' genes have correlation to SPPR above 0.8 and '-C08' is again the opposite. '% of expressed' is the percentage of genes among the group of genes with same expression that belong to the annotation category. '% of annotated' is the percentage of genes among all genes that belong to the annotation category. 'p-value' is for the significance of enrichment. 'Intepretation' is a biological consept to which the genes relate to as intepreted by the authors.

#### Pathway analysis of the metabolic network

In order to specifically understand the metabolism underlying the transcriptomic responses the transcriptome profiling data was studied in the context of the metabolic network of *T. reesei *with the 'Reporter metabolite' [46] and 'Enriched Molecular Path detection' (EMPath) [47] methods. A draft metabolic network for *T. reesei *was constructed by computationally transfering the *Aspergillus niger *[48] network through sequence homology detection (Additional file 2).

**Figure 3:**
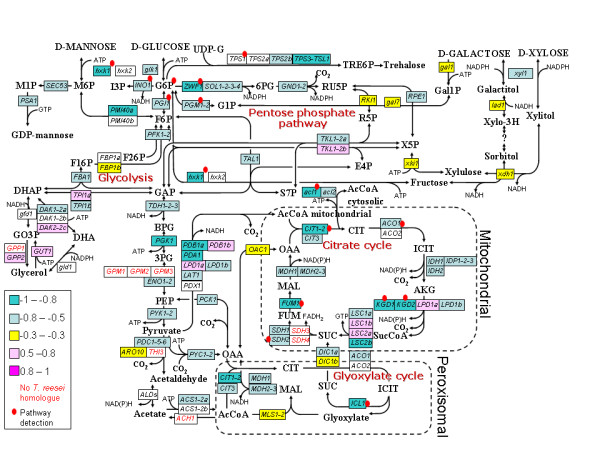
**Correlations of genes in central carbon metabolism with SPPR**. Metabolic network of metabolites and genes (rectangles). Genes are colored according to correlation to SPPR. Gene names are as in *S. cerevisiae*, except that paralogs not found in yeast are distinguished by additional letters (a,b,...) and genes corresponding to many genes in *S. cerevisiae *have names extended with '-'. *T. reesei *gene names have been used when available (*hxk1, glk1 *[65], *lxr1 *[126], *xdh1 *[127], *xyl1 *[128], *lad1 *[129], *gld1, gfd1 *[61], *gal10, gal7 *[130], *xdh1 *[127] ) and others when *T. reesei *nor *S. cerevisiae *name exists (*acl1, acl2 *[93]). Cellular locations are merely indicative. Genes detected by pathway detection are specified with a red dot.

We then applied the 'Reporter metabolite' -method to find metabolites that participate to reactions by enzymes whose gene expression had the highest correlations (positive or negative) to SPPR. In parallel, we used 'EMPath' to detect paths of metabolic enzymes whose gene expression had the highest (positive or negative) correlations to SPPR (Figure 3, Additional file 1, Table S4 and S5). Neither of these methods requires predefined pathways, instead they explore the structure of the network freely. Of the metabolites detected by 'Reporter metabolite' using a cut-off of *p <*0.05 and with more than one adjacent gene and enzyme in the metabolic network, glucose 6-phosphate in upper glycolysis, alpha-ketoglutarate in TCA cycle, 5, 10-methenyltetrahydrofolate and tetrahydrofolate related to folate metabolism, 3-(4-hydroxyphenyl)pyruvate related to secondary and amino acid metabolism and D-glucosamine related to chitin biosynthesis were detected. In addition we detected two sets of genes by 'EMPath' using a cut-off of *p <*0.025: genes of the TCA cycle and a set of connected genes from glucose 6-phosphate to amino acid biosynthesis. However, the second set is not likely to truly transfer atoms. One chitin synthase had the only significant positive correlation of gene expression to SPPR among the genes detected by pathway analysis. Other significantly correlated genes, including another chitin synthase, had negative correlations. Given the emphasis on the TCA cycle and upper glycolysis in the pathway analysis results, we further annotated genes of central carbon metabolism to detect paralogs and orthologs in particular in relation to *S. cerevisiae*. We found 11 cases where *T. reesei *has a paralog, i.e. an isoenzyme, not found in *S. cerevisiae*. In 7 of those pairs the two paralogs have an absolute correlation to the SPPR above 0.5, but with opposite direction of correlation (Figure 3).

#### Chromosomal clusters

As secondary metabolism genes are known to occur in chromosomal clusters and were enriched in genes with significant positive correlation to SPPR we tried to detect such chromosomal clusters. Starting from the pairs of adjacent genes with absolute correlation to SPPR above 0.7 and of the same direction of correlation, we looked for such triplets of correlated genes and expanded the triplet by adding genes to the cluster untill the direction of correlation in subsequent genes changed.

**Figure 4:**
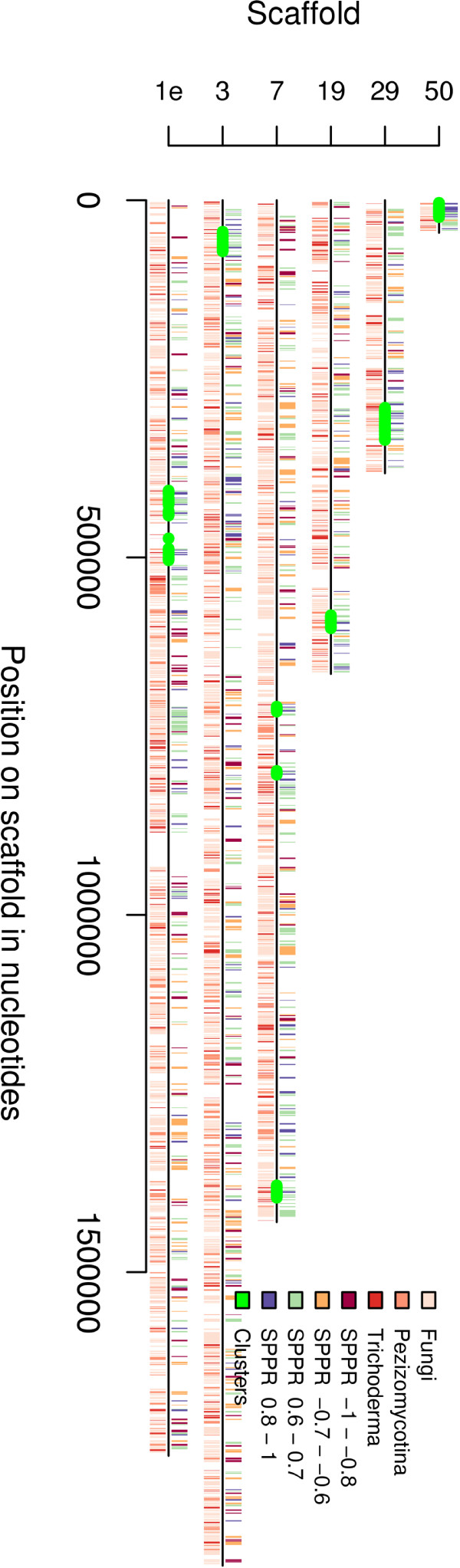
**Chromosomal gene clusters with positive correlation with SPPR on scaffolds**. The Y axis shows the scaffold. Only end of scaffold 1 is shown i.e. 2 - 3.75 Mb (1e). Only scaffolds with chromosomal gene clusters are shown. The × axis shows the nucleotide position on scaffold. Below each scaffold (black line) a color coding is shown for taxonomic specificity as in Figure 3. Above each scaffold a color coding is shown for gene expression correlation to specific protein production rate. Detected clusters (Additional file 1, Table S6) are highlighted in green.

In total nine clusters with positive correlation (c1:c9) to SPPR and seven clusters with negative correlation (cn1:cn7) to SPPR were detected (Figure 4, Additional file 1, Figure S6, Additional file 1, Table S6 and S7). Of the chromosomal clusters with positive correlation to SPPR, three contain typical secondary metabolism genes (c1: non-ribosomal peptide synthase; c9: polyketide synthase; c7: trichothecene C-15 hydroxylase homologue [49] ). Four contain carbon source degradation and uptake related genes (c2: *xyn1 *[50], *ThPg1 *[51] homologue and two other CAZymes; c3: *trhxt1 *[52] and three other putative transporters; c6: glucose-methanol-choline (GMC) oxidoreductase; c7: *cbh1 *[53] and three putative transporters). Of these c1, c6 and c9 contain putative transcription factors of the Zn2Cys6-family. Of the chromosomal clusters with negative correlation to SPPR three contain homologues of genes involved in regulation in *S. cerevisiae *(cn1: *SIP3*, which protein is an interaction partner of Snf1p and cn2: *SNF1*; cn4: *SPT10 *histone acetylase), one in *Schizosaccharomyces pombe *(cn3: *srk1/mkp1 *[54] signalling related protein kinase, cn4: *ace2 *[55] cell division transcription factor) and two in *Emericella nidulans *(cn6: *pldA *signalling related protein [56]; cn7: *oefA *[57] signalling related protein). Homologues of *S. cerevisiae *protein biosynthesis, transport and secretion related proteins are found in six negatively correlating clusters (cn1: putative translation termination factor, cn3: *AGE1 *secretory pathway GTPase and putative translation initation factor; cn4: putative initation factor; cn5: *UBC6 *ER-associated degradation protein; cn6: *BRO1 *vacuolar sorting protein and cn7: *BET3 *transport protein particle complex protein).

In relation to central carbon metabolism a putative D-xylulose 5-P/D-fructose 6-P phosphoketolase (77481) in cn4, also detected by proteomics (Additional file 1, Table S10) and the *ICL1 *isocitrate lyase in cn2, also detected by pathway analysis, are of note (Additional file 1, Table S5).

We also checked whether any of the chromosomal clusters were conserved in other fungi and found that only the negatively correlated cluster cn7 was conserved outside Hypocreales (Additional file 1, Figure S7, Additional file 1, Table S8). Four genes (*oefA*, *BET3*, CCHC Zn finger protein and WD-repeat protein that is a putative homologue of *S. pombe *splicing factor *Spf38 *[58]) of the five genes in cn7 were found close to each other in seven of the 14 studied Pezizomycotina species, which cover species of the subphyla widely.

#### Differentially expressed novel genes detected with sparse arrays

In addition to the predicted genes of the *T. reesei *genome, the microarray used in this study included probes that covered the intergenic regions of the genome with approximately 100 b gap between consecutive probes. Previously we described a method for analysing data from signals of these probes in order to discover novel genes and proposed that we had found novel transcripts of putative regulatory genes [59]. Of the 125 novel genes detected in [59] (Additional file 1, Table S9) 16 were differentially expressed between the cultivation conditions (*D *= 0.03 h^-1 ^low cell density (D03)/*D *= 0.06 h^-1 ^low cell density (D06) none; D03/*D *= 0.03 h^-1 ^high cell density (HD) 12 higher in D03 and three higher in HD; D06/HD five higher in D06 and one higher in HD). In addition to the above, three had a correlation to SPPR over 0.8 and none below -0.8. Eight of the above mentioned 19 genes are neighbours of a gene or genes with a putative role in regulation or signalling. Novel genes between a homologue of *MOB2 *and a putative bZIP transcription factor, between the orthologue of *RIO1 *and Zn2Cys6 transcription factor and adjacent to the orthologue of *DOC1 *and *seb1 *[60,61] were higher in D03 than HD cultivations. One novel gene between a homologue of *OPY2 *and a putative Ras like GTPase was higher in HD than D03 cultivations and higher in D06 than D03 cultivations. Novel genes adjacent to a homologue of *CHD1 *and a putative GPCR [62] had a correlation to SPPR higher than 0.8.

### Proteome analysis

In order to study the effects of specific growth rate and cell density on the proteome of *T. reesei*, as well as to asses how the transcriptomic responses were reflected in the proteome, we carried out 2D gel electrophoresis analysis of the chemostat cultivations. Cell extracts from triplicate cultivations of *D *= 0.03 h^-1 ^low cell density condition (D03), *D *= 0.06 h^-1 ^low cell density condition (D06) and *D *= 0.03 h^-1 ^high cell density condition (HD) were subjected to analysis using the DIGE method, using pH ranges of 3.0-5.6 and 5.3-6.5 in the isoelectric focusing step followed by 11% w/v SDS-PAGE. We compared D03 to HD for comparison of the effect of cell density and D03 to D06 for comparison of the effect of growth rate.

**Figure 5:**
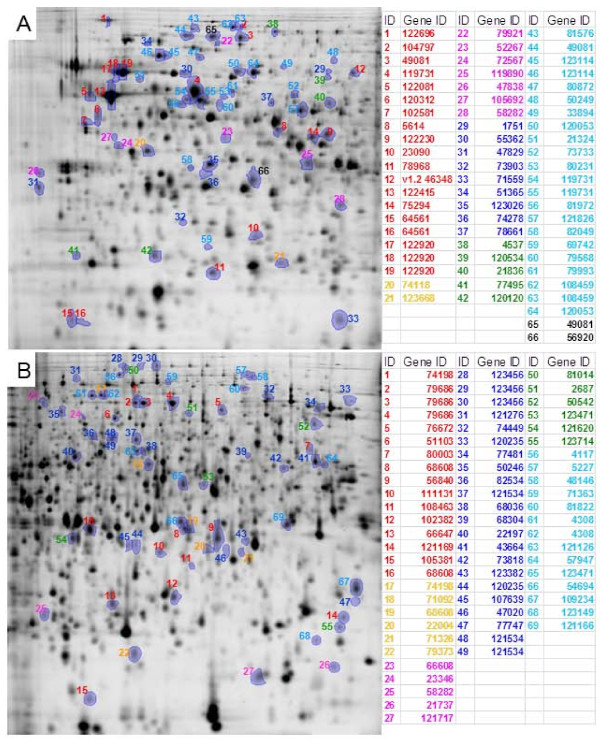
**Identified protein spots in 2D analysis**. In panel 'A' pH interval 3-5.6 in IEF and in panel 'B' 5.3-6.5 in IEF. Protein spots more abundant in D03 cultivations than in HD cultivations in red. Respectively, in D03 than in D06 and HD in orange; in D03 than in HD in pink; in HD than in D03 in dark blue; in HD and D06 than in D03 in green; in D06 than in D03 in light blue; other comparisons in black.

Protein spots showing more than 2-fold significant difference in intensity between the cultivation conditions (significance threshold *p *< 0.05) were subjected to LC-MS/MS analysis for identification (the 2D gel maps of the identified proteins are shown in Figure 5 and the proteins are listed in Additional file 1, Table S10 and S11). In addition to determining the responses of individual proteins, we also searched for enrichment of functional categories among groups of differentially expressed proteins (Table 3) in comparison to all annotated proteins with a cut-off of *p <*0.05.

**Table 3:** Enriched functional categories in proteome analysis

Description	Expression	% of expressed	% of annotated	p-value
Protein secretion	D03/D06	27.8	7.7	0.00
Protein synthesis	-D03/D06	14.6	11.3	0.00
Protein secretion	D03/D06 T0	25.0	4.6	0.01
Glycoside hydrolase	D03/HD	20.0	14.9	0.00
Protein secretion	D03/HD	14.3	7.7	0.01
Stress	-D03/HD	8.3	20.0	0.00
Protein degradation	-D03/HD	11.1	7.0	0.04
Glycoside hydrolase	D03/HD T0	15.8	6.4	0.02

**Figure 6:**
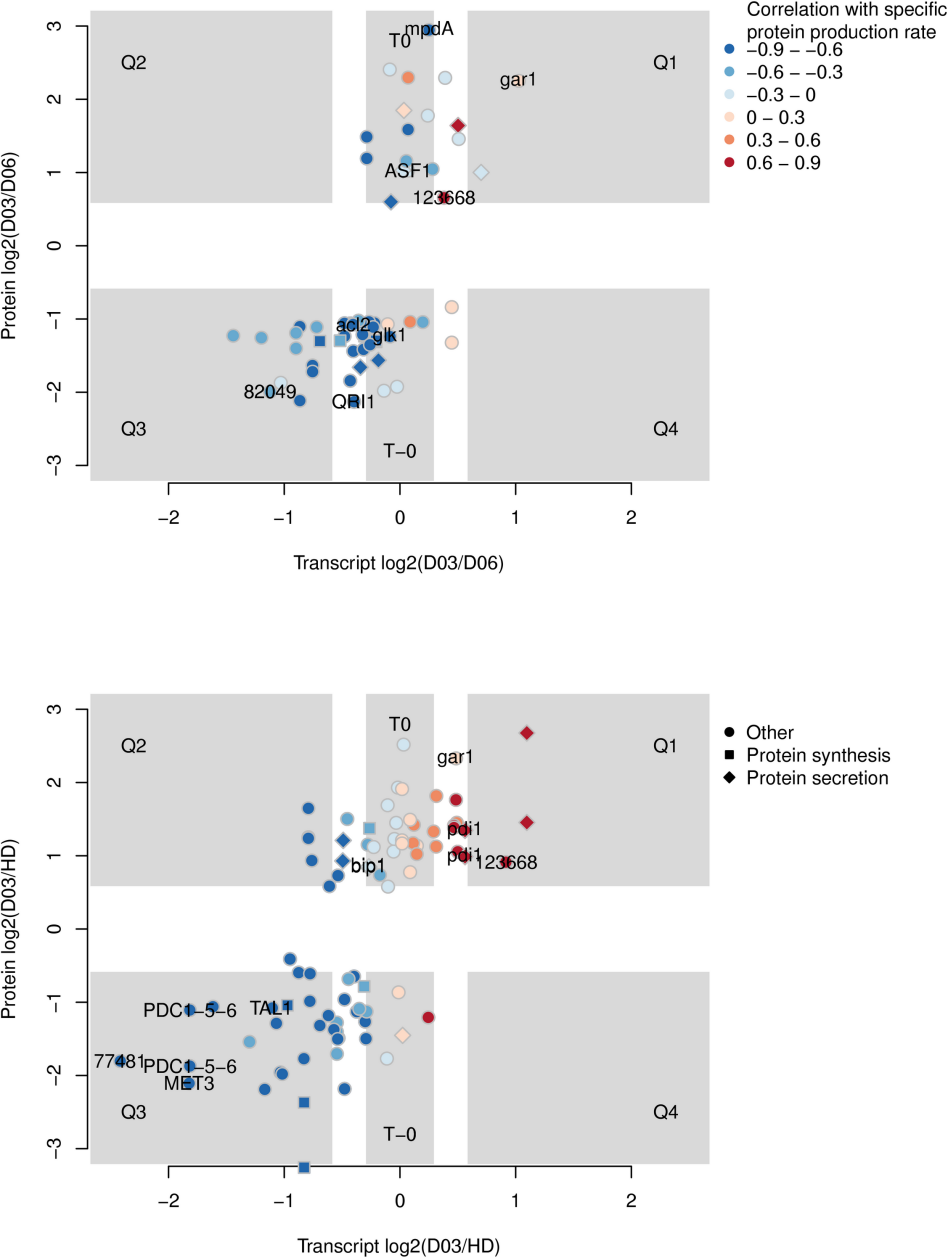
**Correlation between transcriptomics and proteomics data**. The Y axis shows the log2 fold change of protein expression and the × axis the corresponding transcript expression. Upper panel shows a comparison between *D *= 0.03 h^-1 ^low cell density (D03) and *D *= 0.06 h^-1 ^low cell density (D06) and lower panel between D03 and *D *= 0.03 h^-1 ^high cell density (HD). Data points are coloured based on gene expression's correlation to specific protein production rate. Central crosshairs shows the 1.5 (0.6 as log2) fold change cut-off for both axes. In addition, a half of that on log2 scale i.e. 1.2 (0.3 as log2) was used as cut-off for 'non-transcriptionally regulated' assingment (T0, T-0). Q1-4 label the quadrants of transcript-protein relationship classification. Same cut-offs were used for enrichment analysis of proteomics (Table 3) and transcript-protein relationship classification (Additional file 1, Table S10).

'Expression' is type of expression behaviour the proteins have i.e. -D03/D06 stands for proteins more abundant in D06 than in D03. In the two cases labelled with 'T0' and 'T-0' transcripts were not regulated, i.e. the transcript fold change was less than 1.2 (see Figure 6 for further details). See Table 1 for explanations of subsequent columns.

Glycoside hydrolase proteins were detected as enriched in the comparisons (Table 3) and individual predicted secreted proteins were detected as differentially expressed (Additional file 1, Table S10 and S11). However, as the 2D gel analysis carried out did not include extracellular proteins from the supernatant, but only intracellular proteins, we omit predicted extracellular proteins from the detailed analysis presented below.

Comparison of the cultivations with different growth rates (*D *= 0.03 h^-1 ^low cell density condition (D03) and *D *= 0.06 h^-1 ^low cell density condition (D06)) showed differential expression of proteins related to protein secretion and synthesis especially. Proteins involved in protein secretion were enriched among those more abundant in the D03 cultivations as compared to the cultivations of D06, including candidates for a *SGT2*-like ER membrane targeting protein (74118), a Golgi assembly stacking protein (105692), two *α*-1, 2-mannosidases (74198, 79921) putatively involved in glycosylation as well as a protein disulphide isomerase -related protein (119890). The proteins more abundant in D03 cultivations as compared to D06 cultivations also included two proteins with a putative function in chromatin and nucleosome organisation, a GCN5-related N-acetyltransferase (123668) and the *ASF1 *anti silencing protein (47838). A homologue of MPDA [63] mannitol-1-P dehydrogenase (52267) with a putative function in storage carbon utilisation and the D-galacturonic acid reductase (GAR1 [64]) involved in utilisation plant material derived D-galacturonic acid were more abundant in D03 than D06, which could indicate the need for enhanced carbon harvesting under the low carbon feeding conditions.

The functional category enriched among the proteins that were more abundant in D06 cultivations as compared to D03 cultivations was protein synthesis. This group of proteins included several amino acid tRNA synthetases, as well as translation initiation and elongation factors. In addition, the proteins being more abundant in the high specific growth rate cultivations D06 as compared to D03, included other biosynthesis and growth related functions, such as amino acid synthesis (4 proteins), carbon metabolism (3 proteins), purine biosynthesis (1 protein) and cytoskeleton related functions (3 proteins) (Additional file 1, Table S10 and S11). Regarding proteins related to regulation, a protein kinase, a protein phosphatase 2A regulatory subunit, a homologue of Ino1p myoinosotol-1-P synthase, and a GCN5-related histone acetyl transferase were detected as more abundant in D06 than D03 cultivations. Also mitochondrial and cytoplasmic heat shock family proteins had a higher abundance in the D06 than in D03 cultivations. Proteins involved in protein secretion were enriched among proteins more abundant in the low density D03 cultivations as compared to the high density cultivations HD, including proteins involved in folding as well as later stages of protein secretion (*SGT2*-like targeting protein 74118 and a *SNF7*-like sorting/endocytosis protein). In addition, the proteins more abundant in D03 cultivations included e.g. proteins involved in metabolic functions, especially in amino acid synthesis, RNA and DNA related functions (DNA excision, chromatin and RNA binding, Additional file 1, Table S10 and S11).

The proteins more abundant in HD than in D03 cultivations were enriched in proteins with functions related to stress, especially oxidative stress and protein degradation. Several proteins involved in carbon and amino acid metabolism, as well as mitochondrial proteins were also more abundant in HD than in D03 cultivations. Especially the carbon metabolism proteins related to the early glycolytic pathway or pentose phosphate pathway were affected (PGM1-2, TAL1 and SOL1-2-3-4, Additional file 1, Table S10 and S11), along with pyruvate decarboxylase PDC1-5-6.

Responses to different specific growth rate or cell density had many features in common. The analysis revealed 12 proteins that were more abundant in HD and D06 than in D03 cultivations. The proteins had different types of putative functions and included proteins involved in amino acid and amino acid-tRNA synthesis, translation, metabolism of carbon and thiamine or secondary metabolism and oxidative stress (Additional file 1, Table S10 and S11). Of particular note is the putative GCN5-related N-acetyl transferase (120120).

The 7 proteins more abundant in D03 than in HD and D06 cultivations included a putative GCN5-related N-acetyltransferase (123668), GARI a putative *α*-1, 2-mannosidase (74198), and *SGT2*-like ER membrane targeting protein (74118).

#### Comparison of transcriptomics and proteomics data

In order to see how the transcriptomic responses were reflected in the proteome in general, we carried out a correlation analysis of RNA and protein level fold changes. For the comparison of *D *= 0.03 h^-1 ^low cell density (D03) and *D *= 0.06 h^-1 ^low cell density (D06) the overall correlation *r *of transcript and protein fold changes was 0.5 regardless of whether the Spearman or Pearson method was used. Similarly, the correlation was 0.6 for D03 versus *D *= 0.03 h^-1 ^high cell density condition (HD). We then compared fold changes of indidual proteins and transcripts (Figure 6).

The transcript-protein pairs were divided in six (Q1, Q2, Q3, Q4, T0, T-0, see below and Figure 6 for explanation) classes for both comparisons. In order to divide the pairs into these categories two cut-offs were used: 1) a fold change of at least 1.5 (0.6 as log2) was assumed to indicate that a protein or transcript level was differentially expressed and 2) a fold change of less than 1.2 (0.3 as log2) was assumed to indicate that a protein or transcript level was not differentially expressed.

In the comparison of D03 and D06, 2 transcript-protein pairs were found in Q1 (both protein and trancript more abundant in D03), none in Q2 (transcript more abudant in D06, protein more abundant in D03), 9 in Q3 (both more abundant in D06), 12 in T0 (protein more abundant in D03, transcript not differentially expressed) and 17 in T-0 (protein more abundant in D06, transcript not differentially expressed). Proteins related to protein secretion (74118, 105692 and 119890, see above) were found enriched among proteins more abundant in D03 than D06, but transcripts not differentially expressed (D03vD06 T0 in Table 3). In addition, T0 included the Asf1p anti silencing protein (47838) and the MPDA [63] mannitol-1-P dehydrogenase (52267) homologues. The 17 proteins more abundant in D06 than D03, but transcripts not differentially expressed (T-0) included 6 metabolic enzymes (including PGM1-2, ADE5, 7 and GLKI [65]), 2 proteins putatively involved in actin tubulin assembly and 2 amino acid tRNA synthetases.

In the comparison of D03 and *D *= 0.03 h^-1 ^high cell density condition (HD), 2 transcript-protein pairs were found in Q1 (both protein and trancript more abundant in D03), 3 in Q2 (transcript more abundant in HD, protein more abundant D03), 18 in Q3 (both more abundant in HD), 19 in T0 (protein more abundant in D03, transcript not differentially expressed) and 5 T-0 (protein more abundant in HD, transcript not differentially expressed). Glycoside hydrolases were found enriched among proteins more abundant in D03 than HD, but transcripts not differentially expressed (D03vHD T0 in Table 3). Q2 included the only identified protein (v1.2 46348), a homologue of *S. cerevisiae *Sti1p chaperone, that was predicted as gene in genome version 1.2, but not in genome version 2.0. In addition, 79686, a homologue of *S. cerevisiae *Pab1p polyadenylate binding protein and 119731, a homologue of *S. cerevisiae *Hsp60p mitochondrial chaperone, were included in Q2. Proteins more abundant in D03 than HD, but transcript not differentially expressed (T0) included 4 proteins related to amino acid metabolism and BIPI.

We also calculated the translational control efficiency ratio (TCEr) [1] for each protein in both comparisons of conditions (Additional file 1, Table S10) and inspected extremes of the TCEr distributions.

Translational control efficiency (TCE) is a measure of the effective conversion of a gene's transcript into protein, encompassing synthesis and degradation processes, hence TCEr is the ratio of relative changes in translational control efficiencies between the two conditions studied. TCEr can be calculated from genome-wide data, such as ours, although the TCE cannot be.

In the comparison of D03 and D06, 4 proteins, including a HSP78 mitochondrial chaperone (2687) and QRI1 UDP-N-acetylglucosamine pyrophosphorylase (79568) had a TCEr*<*0.33 i.e. they were 3 times more efficiently translated in D06 than in D03. 6 proteins, including the Golgi protein 105692 and MPDA [63] homologue (52267) had a TCEr*>*3 i.e. they were 3 times more efficiently translated in D03 than in D06. In the comparison of D03 and HD, 3 proteins, including the same HSP78 protein (2687) and a translation elongation factor (120235) had a TCEr *<*0.33. 10 proteins, including GARI, a putative mitochondrial HSP60 (119731), protein synthesis initiation factor (111131) and *SNF7*-like protein (121169) had a TCEr*>*3.

The results of enrichment analysis of the proteome agree with the enrichment analysis of the transcriptome with regard to protein synthesis (i.e. correlating negatively with SPPR) and glycoside hydrolases (i.e. correlating positively with SPPR, Table 2, Additional file 1, Table S3 and S10). In enrichment analysis of the transcriptome, peptidases were found to enriched among genes correlating positively with SPPR while proteasome components were among those negatively correlated. In enrichment analysis of the proteome, protein degradation related proteins were found to be enriched among proteins more abundant in *D *= 0.03 h^-1 ^high cell density condition (HD) than in *D *= 0.03 h^-1 ^low cell density condition (D03) (an thus negatively correlating with SPPR). The correlation to SPPR of proteins related to protein secretion is generally negative ('Protein secretion' in Figure 2), while the enrichment analysis of proteome finds 'Protein secretion' enriched among proteins more abundant in D03 than HD or D06 (i.e. correlating positively with SPPR). Notably, *pdi1 *falls to this category.

## Discussion

We have used chemostat cultivations at specific growth rates and cell densities to characterise the transcriptome and proteome of *T.reesei *in order to understand the molecular bases of low growth protein production phenotype. The stability of the cultivations was monitored with online and off-line measurements, including a monitoring for stability of transcription of the 31 reporter genes covering essential cellular processes [66].

We used the strain Rut-C30, instead of the sequenced QM6a strain, due to its improved protein production capabilities. The mutations in Rut-C30 in comparison to QM6a have been described genome wide [67,68] and the phenotype of three of them have been studied. For the single major multi gene deletion of Rut-C30 in scaffold 15, it has been shown that it has no impact to cellulase production on lactose containing medium [68]. The glucosidase II alpha subunit frameshift of Rut-C30 improves protein production by changing the glycosylation pattern of secreted proteins [69]. The *cre1 *truncation weakens carbon catabolite repression of Rut-C30 [70]. Given that we used a non-repressive carbon source, lactose, and no residual glucose was detected and we try to describe the cellular response to protein production, these mutations do not interfere, but rather might improve our study. However, without additional data, for example transcription profiling data of QM6a from the same conditions, it is very hard to deduce, what would be the impact of other yet uncharacterised mutations to the results of our study. We inspected the 967 genes which expression correlated significantly with SPPR. We found that 8 genes correlated positively and 11 negatively, had a substitution in them or in promoter or terminator regions as defined by [67]. Among them only one possible regulator was found, gene 123344 that was negatively correlated to SPPR. It is an orthologue of *S. cerevisiae YPD1 *histidine phosphotransferase involved in osmotic stress. Hence, mutations in Rut-C30 are not likely to have major direct effects to gene regulation in our experiment. Although some details of our results may turn out in time to be strain specific, in light of the current understanding of the phenotype of Rut-C30 it does not seem likely that the general results presented in this paper would be. Also, the low growth rate protein production phenotype we study is not specific for NG14/Rut-C30 strain lineage, but has been described for two other strains of the QM9123/QM9414 lineage [30,31].

We used a correlation-based analysis of gene expression, as has been used previously for *S. cerevisiae *chemostat cultivations of variable growth rate and nutrient availability [2]. This allowed us explicitly to concentrate on our main interest, the correlation of specific extracellular protein production rate (SPPR) and intracellular processes and gives us a single number per gene that defines the genes behaviour in the three conditions, instead of several numbers from pair-wise comparisons. Such an analysis is not sensitive to variations in cultivations provided that adequate parameters have been quantified, as is possible in bioreactor cultivations.

In comparing enrichment analysis of genes that significantly correlate to SPPR and genes with significantly different fold changes between different conditions, we see similar trends for example for genes of secondary metabolism and secreted proteins (positive correlation, higher in *D *= 0.03 h^-1 ^low cell density condition (D03) and *D *= 0.06 h^-1 ^low cell density condition (D06) than in *D *= 0.03 h^-1 ^high cell density condition (HD)) and genes of protein secretion, cytoskeleton and primary metabolism (negative correlation, higher in HD cultivations than in D03 and D06 cultivations, Table 2 and Additional file 1, Table S3). It was not possible to detect significant differences between D03 and D06 cultivations by analysing fold changes alone, thus we could not say whether specific growth rate also affected expression of these genes. In contrast, our analysis of genes with correlation to SPPR clearly reveals that both cell density and specific growth rate contributed to the SPPR.

We found that the highest specific protein production rate was achieved with low cell density cultivations at specific growth rate of *D *= 0.03 h^-1 ^(D03). Transcriptomics and proteomics suggested that the effect resulted from high transcript, and hence protein, levels of the secreted enzymes (Table 2, Figure 6, Additional file 1, Table S10 and S11) in *D *= 0.03 h^-1 ^low cell density (D03) cultivations. Secreted glycoside hydrolases were detected as enriched among proteins more abundant in D03 than in *D *= 0.06 h^-1 ^low cell (D06) or *D *= 0.03 h^-1 ^high cell density (HD) cultivations. As we carried out intracellular proteomics the higher amount of the secreted proteins in the intracellular samples could be either due to corresponding increased synthesis of the protein or due to limitation in transport of the proteins as described in [32] based on in vivo labelling experiments.

In comparison to [32], in which similar cultivations of variable growth rate were studied, we found the same direction of expression change of specific marker genes such as *bip1 *[71] and *pdi1 *[45]. We also found that their proteins were more abundant in *D *= 0.03 h^-1 ^low cell density (D03) than in *D *= 0.03 h^-1 ^high cell density (HD) cultivations (Figure 6 and Additional file 1, Table S10). In [32] it was proposed that the Unfolded Protein Response (UPR) would be active in D03 in response to increased production of secreted proteins. UPR has been described as a large scale induction and modification of the protein secretion pathway involving hundreds of genes [72]. We detect no clear UPR in response to high protein production, rather genes of the protein secretion pathway generally had a negative correlation to SPPR, *pdi1 *being one of the few exceptions to this rule. In *S. cerevisiae BIP1 *and *PDI1 *have been shown to be poor indicators of UPR [73]. In *T. reesei bip1 *and *pdi1 *expression levels have been shown to correlate well with *hac1 *splicing, indicating UPR, in a batch cultivation [74]. As we studied steady state rather than induction conditions, the high level of *bip1 *and *pdi1 *in D03 cultivations could be a remnant of an earlier full UPR. In general our transcriptomics and proteomics data agree well, as we detected for both proteomic comparisons a correlation of *>*0.5 between transcript and protein fold changes, while correlations from 0.4 - 0.7 [1] to 0.21 [75] have been reported earlier. However, when we classified transcript-protein pairs into groups by their expression we found that in most cases the transcript of a differentially expressed protein was not differentially expressed with the strict cut-offs used. As it is known that the normalisations used for transcriptomics can damp fold changes [76] it is hard to know whether this is a true biological phenomena or a data analysis artefact. However, the used classification allowed us to pick outstanding transcript-protein pairs. Three genes were found to have more abundant transcripts in *D *= 0.03 h^-1 ^high cell density (HD) than in *D *= 0.03 h^-1 ^low cell density (D03) cultivations, while their proteins had opposite abundance (Figure 6, D03/HD, Q2). Two of them are homologues of *S. cerevisiae *Pabp1 and Sti1p that belong to a RNA-binding protein network regulating posttranscriptional and posttranslational events of protein synthesis [77]. Sti1p acts as chaperone, while the third of them is a homologue of *S. cerevisiae *Hsp60p, a mitochondrial chaperone. Pab1p has been shown to be involved in control of translation initiation in response to metabolic state of cell [78]. Transcript-protein pairs with non-correlated expression could be required for fast responses to changes in environment [79]. Taken together, the differential expression and predicted function of these genes suggests them as actors in regulation of translation in response to metabolic state of cell. In addition, we have observed condition dependent abundance variation in different pI isoforms of Hsp60p (119731, Figure 5).

Enrichment analysis of proteomics highlighted in particular key processes and genes related to protein production. In the transcriptome peptidases were positively correlated and proteasome components negatively correlated to SPPR (Table 2). In the proteome division between peptidases and proteasome components was not evident. Nevertheless both positive and negative correlations of individual protein degradation related proteins were detected in proteomics (Table 3). Proteins related to the machinery of protein secretion were enriched among proteins more abundant in *D *= 0.03 h^-1 ^low cell density (D03) than *D *= 0.06 h^-1 ^low cell density (D06) and *D *= 0.03 h^-1 ^high cell density (HD) cultivations. These included *α*-1, 2-mannosidases and glycosylation related genes. While on transcriptomics side, 9 out of the 16 genes that had a correlation with SPPR above 0.7, were also related to glycosylation. Furthermore, protein secretion related proteins were implicated as targets of posttranscriptional regulation since their protein abundance appeared to be differentially expressed, while the transcript abudance was not (Table 3, Figure 6). Chitin metabolism was detected by pathway analysis and the genes had, notably, both negative and positive significant correlations to SPPR. Thus, proteins of the protein secretion machinery detected by proteomics and the 16 genes of protein secretion machinery which had a correlation to SPPR *>*0.7 may highlight key activities of protein secretion machinery which are required for especially high level of protein secretion.

### Primary biosynthetic functions are expressed at low level in low growth rate protein production

From analysis of distribution of correlation of gene expression to SPPR we found that genes in metabolism, protein secretion and known mitochondrial genes were generally expressed at low level when protein production rate was high in *T. reesei *(Figure 2). By the gene set enrichment analysis we could further see that some protein biosynthesis, cytoskeleton and protein degradation genes behaved similarly. We then constructed a draft metabolic network model for *T. reesei *(Additional file 2) in order to analyse transcriptional responses in the network's context by pathway analysis. In particular, we found that genes involved in the TCA cycle and related to glucose 6-phosphate, i.e. in upper glycolysis, were typically expressed at low level in high production (Figure 3 and Additional file 1, Table S3, S4 and S5). The expression of *ZWF1 *glucose-6-phosphate dehydrogenase (Figure 3), the key branching point enzyme between glycolysis and the pentose phosphate pathway and responsible for producing NADPH required for growth, had a particularly high negative correlation (-0.85) with SPPR. The negative correlation of the genes involved in TCA cycle to SPPR was detected with the two pathway analysis methods and also in analysis of significantly changing genes between different cultivation conditions.

Transcript and protein abundance changes of pentose phosphate pathway (*tal1*, *sol1-2-3-4*), glycolysis (*pgm1-2*, *pcd1-5-6*, *glk1*) genes and *ade5, 7 *and *acl2 *were coherent (Figure 3 and 5 and Additional file 1, Table S10). *pgm1-2*, phosphoglucomutase, and *ade5, 7*, purine biosynthetic protein, were detected by the pathway analyses and were negatively correlated with SPPR. Thus, central carbon metabolism is overall expressed at low level under high protein production conditions on transcriptional but also on proteome level.

Of special interest are the paralog pairs, i.e. isoenzymes, with opposite direction of correlation to SPPR, found in the central carbon metabolism (Figure 3). However, the correlations were not significant in all of the pairs. Proteome analysis in *S. cerevisiae *suggested clearly defined functional separation for paralog pairs [80]. Even the modest correlations detected for these pairs may highlight key enzymes required for high rate of protein production.

We looked for chromosomal regions i.e. clusters with particular correlation to SPPR. We found 7 chromosomal clusters with negative correlation to SPPR (Additional file 1, Figure S6 and Additional file 1, Table S7). Only one of these clusters was found to be conserved in Pezizomycotina (Additional file 1, Figure S7, Additional file 1, Table S8). The cluster contains secretion and regulation related proteins and thus could be particularly essential for Pezizomycotina. Together these chromosomal clusters contained a wealth of regulators, in particular three with links to carbon source dependent regulation (*SNF1*, *SIP3 *interaction partner of Snf1p and *pldA *[56]).

In *S. cerevisiae *the *SNF1 *complex is activated by carbon derepressed conditions. Likewise, induction of *pldA *homologues in derepressed conditions have been shown [56]. In *T. reesei snf1 *appears not to phosphorylate *cre1 *and its expression is possibly independent from the carbon source [81]. Given its strong negative correlation to SPPR (-0.87) in our study, *SNF1 *could be an active regulator in the non carbon catabolite repressed growth conditions we studied.

### Plant biomass degradation and secondary metabolism genes are induced together

As expected cellulases and other plant biomass degradation related proteins were found to have a positive correlation to SPPR. Genes of predicted secreted proteins in general, and sugar transporters in particular, were found to have a positive correlation (Table 2 and Figure 2). Many genes possibly involved in secondary metabolism were found also to have a positive correlation with SPPR. Accordingly, we identified chromosomal clusters positively correlated to SPPR containing the above mentioned genes (Figure 4 and Additional file 1, Table S6). Seven (c1, c2, c3, c6, c7 and c8) of our nine clusters overlap with cellulase clusters previously predicted for *T. reesei *[14]. Also several of the clusters found by [14] were reported to contain genes encoding proteins involved in secondary metabolism.

The transition to stationary phase in batch cultivations is known to activate secondary metabolism, such as aflatoxin synthesis in Aspergilli [82], in most fungi. To our knowledge the joint induction of secreted proteins and secondary metabolism has not been previously shown. However, the physiology at specific growth rate of optimal protein production (*D *= 0.03 h^-1 ^and low cell density) for *T. reesei *may be similar to the physiology of transition phase in batch cultivation. Further, in both the batch deceleration phase and the chemostat, inducing carbon source would be present in low concentration.

### Lineage specific response to low upper glycolytic or TCA cycle flux?

From analysis of distribution of correlation of gene expression to SPPR we found that not only functional categories had distinctive correlations, but also that genes with positive correlation to SPPR tended to be more lineage specific than genes with negative correlation (Figure 2). Furthermore, we found that genes with significant positive correlation to SPPR had significantly lower maximum expression, fold change, GC% and distance to scaffold end than non-correlated genes (Table 1, Additional file 1, Figure S3). In *Aspergillus oryzae *genes in non-syntenic blocks have particularly low expression levels, but they have particularly high fold changes in conditions inducing plant biomass degradation enzymes [12,20]. Genes in non-syntenic blocks and belonging to protein families enriched in Pezizomycotina [5] tend to be found in or near subtelomers and are particularly short in *Aspergillus fumigatus *[15,17]. In *Nectria haematococca *the GC% of lineage specific genes, particularly of those found in supernumary chromosomes, is lower than that of conserved genes [11]. Thus, the characteristics of genes positively correlating to SPPR agree in regard to protein families, low maximum expression, GC% and chromosomal position with previous descriptions of lineage specific genes found in non-syntenic blocks. However, the fold change trend was opposite in *T. reesei *to that in *A. oryzae *and we did not find a significant difference on gene length, in contrast to NSB genes *A. fumigatus*. These differences could reflect either intrinsic differences between Sordariomycetes and Eurotimycetes or differences due to experimental conditions. The lower GC% could reflect only the lack of codon optimisation, i.e. low expression, but in that case one would expect larger variation around the average GC% rather than a different average GC%. Notably, the average GC% falls even lower when considering only pairs of adjacent positively correlated genes, highlighting the link to genome structure. Gene expression levels do not necessarily correlate with enzyme activities in the cell and hence with the cellular flux of the catalysed reactions. In particular, chemical reactions and changes of enzyme activity happen on much smaller time scales than changes in gene expression. Regardless, transcriptional profiling data has been succesfully used to constrain fluxes in flux balance analysis (FBA) in bacteria [83-85] and algae [86]. Results from these studies suggest that when considering physiologically clearly distinct steady states, such as the conditions we studied, transcriptome profiling data may have, at least at the pathway level, a meaningful correlation to flux.

In *S. cerevisiae*, expression of essential and conserved genes is positively correlated with growth rate and hence, in the particular conditions studied flux through primary metabolism to biomass [1]. We are interested on SPPR rather than growth rate and our data was generated from only two different growth rates. However, we detected a strong negative correlation of gene expression, in particular, of upper glycolysis and TCA cycle, and in general of primary metabolism with SPPR.

Thus, in the conditions we studied SPPR could be strongly negatively correlated with flux through these pathways and thus a more fundamental major determinant of SPPR could be, for example flux through the upper glycolysis or TCA cycle, rather than growth rate. In that case our results and results from *S. cerevisiae *growth rate experiments would be in agreement conserning conditions of high flux through the primary metabolism. The basic biosynthetic machinery, conserved throughout eukaryotes, is induced when high flux to biomass is possible in order to maximise growth. However, the regulation of TCA cycle genes specifically is species specific [87-89] depending on whether a species favours fermentative or respiratory metabolism in the presence of glucose or, possibly, in high enough glycolytic flux.

In *S. cerevisiae *the set of genes with negative correlation to growth rate, i.e. flux to biomass in the experiment in question, is enriched in genes of unknown function [1]. Similarly, we detect that genes with no known domain tend to have negative correlation to SPPR (Figure 2). In addition, we detect that lineage specific genes with characteristics typical to genes in lineage specific genomic regions (Table 1, Additional file 1, Figure S3) and carrying out functions enriched in Pezizomycotina lineage [5] (Table 2) had a positive correlation to SPPR and thus possibly a negative correlation to flux to biomass. Similar lineage specific induction response to low flux to biomass, i.e. starvation or carbon limitation, are well known also from Eurotiomycetes [20,21], that are not known to exhibit low growth rate protein production.

Thus, our results support the conclusion in [1] of existence of a conserved core protein machinery governing cell growth in Eukarya. Furthermore, we propose a novel lineage specific response possibly to low flux to biomass, or more specifically low upper glycolysis or TCA cycle flux, in Eukarya. In these conditions, lineage specific functions are induced. These functions are essentially what make the phenotype and evolutionary niche of an organism, but they are typically poorly understood as study of lineage specific genes has been negleted in molecular biology [90].

### Regulation of the lineage specific response?

Metabolic fluxes have been suggested not only to be set by regulation but also to participate in the regulation via specific metabolite pools acting as flux sensors [91]. It has been proposed in mouse that the flux of the TCA cycle directly controls gene expression via acetyl-CoA whose concentration in the nucleus plays a key role in nucleosome acetylation [92]. In particular, the control of upper glycolysis genes by acetylation was shown, demonstrating a feedback control loop from the TCA cycle to glycolysis.

Saccharomycotina, except for *Yarrowia lipolytica*, do not have the necessary enzyme, for acetyl-CoA production in cytosol from the TCA cycle, ATP-citrate lyase (ACL). However, it is generally found in other fungi as two genes (*acl1 *and *acl2*) encoding the N and C terminal domains separately [93,94]. Regardless, a similar link between acetyl-CoA concentration and regulation of gene expression exists in *S. cerevisiae *through the acetyl-CoA synthases, commonly found in fungi [95]. The depedency of histone acetylation on ACL has been shown in the Sordariomycetes *Gibberella zeae *[96]. In *T. reesei *it has been shown that *cbh1 *and *egl1 *transcription is dependent on mitochondrial function [97].

**Figure 7:**
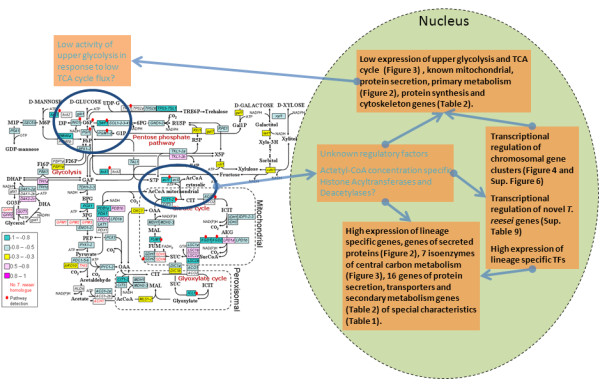
**Major responses in low growth rate protein production and their hypothetic regulatory relationships in T. reesei Rut-C30**.

Given that lineage specific genes tend to occur in specific genomic regions and clusters and especially the control of secondary metabolism clusters has been linked to nucleosomal modifications, nucleosome acetylation would offer a convenient mechanism to control chromatin structure in lineage specific regions and hence, induce or derepress lineage specific genes in conditions where the flux to biomass production is low. Vice-versa, as suggested by the experiments done with mouse [92], acetylation could tune the gene expression of the upper glycolysis and TCA cycle genes to correlate with the flux through the corresponding enzymes, especially under steady state conditions (Figure 7). Undoubtly numerous other intra- and extra cellular signals would be integrated by regulatory networks to enable finer condition specific control of gene expression. Namely the TOR and SNF1 networks discussed above and the velvet and AP-1 networks to integrate information on light, developmental status, oxidative stress etc. (for review [98,99]). Interestingly, the velvet complex protein VeA has been proposed to regulate distribution of carbon flow between primary and secondary metabolic functions [100].

Pezizomycotina genomes are enriched in Zn2Cys6 transcription factors and many of them reside close to the biomass degradation and secondary metabolism genes. However, general mechanisms for their control are not known. A general opening of lineage specific chromosomal regions or more specifically promoters of individual lineage specific genes through histone acetylation could be a major inducing factor in Zn2Cys6 transcription factor transcription. Zn2Cys6 transcription factors could then induce further actual lineage specific, often neighbouring, enzyme genes.

In general histone acetylation and acetylase occupancy at promoters positively correlates with transcriptional activity in *S. cerevisiae *[8]. However, acetylation patterns and their correlation to gene expression can also be gene specific [101]. With on average at least 30 GCN5-like histone acyltransferase family members, thrice more than in Saccharomycotina, Pezizomycotina genomes are well equipped [5] for gene and condition specific histone acetylation. In our data GCN5 family genes were enriched among genes at higher level in *D *= 0.06 h^-1 ^low cell density condition (D06) than in *D *= 0.03 h^-1 ^high cell density condition (HD) (Additional file 1, Table S3) and two were detected with proteomics.

Number of other regulators that could control the responses we detect arise from our data. Namely, *snf1*, not likely having a role in induction of carbon catabolite repressed genes in *T. reesei *[81], could instead be involved in flux or carbon limitation based control of transcription. *PNC1*, required for life span extension by calorie restriction [43], would be a natural candidate for regulation at slow growth rate and *ASF1 *for anti silencing lineage specific silent loci.

A lineage specific response would be likely to involve also lineage specific regulators. Pezizomycotina lineages typically have numerous lineage specific Zn2Cys6 transcription factors [5]. The novel genes discovered from this data set, proposed to be novel regulators [59] and shown to have correlations to SPPR are also prime candidates.

## Conclusions

We present the first genome-wide analysis correlating gene expression and specific extracellular protein production rate (SPPR) and confirm and extend our results with proteomics. We have previously described in detail the protein coding content of Pezizomycotina genomes [5]; in the current work we link lineage specific genomic content to its regulation. We propose that low flux through upper glycolysis or the TCA cycle, resulting in low flux to biomass, is a more fundamental determing factor of protein production at low specific growth rate than the growth rate itself. In addition we propose a response to this flux state, i.e. the lineage specific response (LSR), a large scale induction of lineage specific genes and regulatory factors for it. If our hypothesis about the role of flux in regulation of protein production in *T. reesei *and the lineage specific response holds, the open question is, what then controls the flux? Our data suggests that it is not only the growth rate, but also the density of cells.

Although, protein production at low specific growth rate might be an exception limited to Sordariomycetes, literature from Fungi and Bacteria suggests that lineage specific response to low flux to biomass is a wide spread phenomena. Most previous genome wide studies in Fungi with defined growth rates have been limited to *S. cerevisiae*. With its small genome, minimal number of lineage specific genes [5] and lack of the potential key enzyme, ATP-citrate lyase, detecting the lineage specific response in *S. cerevisiae *could be difficult.

## Methods

### Bioreactor cultivations

*Trichoderma reesei *strain Rut-C30 [102] was grown in chemostat cultivations as described in [103]. Strain Rut-C30 was used instead of the sequenced strain QM6a for its enhanced protein production capabilites. Cultivations were done in lactose-limited chemostats in three conditions: specific constant growth rate of 0.03 h^-1 ^(D03) or 0.06 h^-1 ^(D06), both with low cell density and 0.03 h^-1 ^with high cell density (HD). The high cell density was achieved by increasing the lactose concentration of the feed medium from 10 g/L to 40 g/L. Triplicate cultivations were analysed for the three conditions.

The medium contained: KH_2_PO_4 _15 g l^-1^, (NH_4_)_2_SO_4 _5 g l^-1^, CaCl_2_x2H_2_O 0.6 g l^-1^, MgSO_4_x6H_2_O 0.6 g l^-1^, CuSO_4_x5H_2_O 30 mg l^-1^, FeSO_4_x5H_2_O 5 mg l^-1^, MnSO_4_xH_2_O 1.6 mg l^-1^, ZnSO_4_x7H_2_O 1.4 mg l^-1^, CoCl_2_x6H_2_O 3.7 mg l^-1 ^and lactose 20 or 80 g l^-1^. Salt concentrations in cultivations containing 80 g lactose l^-1 ^were increased to: KH_2_PO_4 _15 g l^-1^, (NH_4_)_2_SO_4 _12.5 g l^-1^, CaCl_2_x2H_2_O 1.5 g l^-1^, MgSO_4_x6H_2_O 1.5 g l^-1^, CuSO_4_x5H_2_O 30 mg l^-1^, FeSO_4_x5H_2_O 12.5 mg l^-1^, MnSO_4_xH_2_O 4.0 mg l^-1^, ZnSO_4_x7H_2_O 5.6 mg l^-1^, CoCl_2_x6H_2_O 14.8 mg l^-1^.

The stability of several physiological parameters was monitored before and after the onset of continuous medium feeding to evaluate the steady state of chemostat cultivations. Standard on-line (e.g. base consumption, dissolved O_2 _concentration and off gas concentrations for CO_2_, O_2_, N_2_) and off-line (e.g. dry weight, NH_3 _concentration, cellulase activity) measurements were used. In addition to these conventional process analyses a novel method for rapid transcriptional profiling called TRAC [42] was used to monitor gene expression of 31 genes and chemostat stability [103].

The TRAC probe set included genes related to product formation (123989:*cbh1*, 122081:*egl1*, 120749:*bgl2*, 80240:*bga1*), various stress responses (e.g. 122920:*bip1*, 122415:*pdi1*, 21745:*hsp105*, 62100:*hsp30*, 50039:SOD2, 79565:TRR1, 104135:*trx2*, 65290:*cpc1*, 120676:NTH1, 62040:*nsf1*), central carbon metabolism (73774:ACS1-2a, 120568:ENO1-2, 119735:TDH1-2-3/*gpd1*, 48707:TPS2b), growth and conidiation (46490:RPS16B, 124010:RPL16A, 34312:*con6*, 51492:*chs1*, 121491:*ccg9*), proteases (121495:*aep1*, 77579:VPA1, 60676:MCA1), oxygen regulation (123382:*hem6*, 55362:*hsp70*) and transport (122043:*ctaA*, 51110:GAP1).

Stable chemostat cultivations were attained within two or three residence times, and three generations after steady states were attained, samples were withdrawn for transcriptomic and proteomic analyses. Smaller samples were taken 1-3 times per day and used for limited transcript analysis with TRAC, dry weight and enzyme activity measurements. CO_2 _production in the fermentor was measured on line. Samples were collected and analysed as in [103]. Briefly, samples were withdrawn from the fermentor rapidly, filtered, washed and the biomass frozen with liquid nitrogen and stored at -80°C. The supernatant was also frozen in liquid nitrogen and stored at -80°C for protein, sugar and enzyme analyses. For determination of dry weight, two sample aliquots were weighed, collected by filtration, washed and dried to constant weight at 105°C. Residual lactose, glucose and galactose in the cultivation filtrate was measured enzymatically (Lactose kit, Roche, Basel, Switzerland). Soluble protein concentration was measured using the Bio-RAD Protein assay (Hercules, CA). The ammonium concentration was determined using the Roche ammoniak test kit (Basel, Switzerland) adapted for automated analysis with the Cobas-mira (Roche). The activities of the cellulolytic enzymes were measured as in [103].

### Microarray analysis of transcriptome

Microarray analysis was carried out as in [59]. Briefly, total RNA was extracted and submitted to microarray analysis by Roche Nimblegen (WI, USA). Probe design and synthesis, RNA labelling, hybridisation and signal quantification were carried out by Nimblegen. Design of the microarray and analysis of microarray data were carried out with the *T. reesei *genome [14] version 1.2 [104]. Each of the 9997 genes were covered with 11 25mer probes.

In addition the, plus strand of intergenic regions were covered with 187, 641 25mer oligonucleotide probes with approximately 100 nt spacing as described in [59].

The data has been submitted to GEO with accession number GSE30458.

Annotation of the genes and all homology based analysis were carried out with genome version 2.0 [105] unless otherwise stated, provided the gene was found among the 9129 genes of version 2.0.

### 2D-gel analysis of proteome

Mycelial samples from steady state chemostat cultivations were collected by filtering through Whatman GF/B filters, washed with 0.9% w/v NaCl, frozen immediately in liquid nitrogen and stored at -80°C. The cells were disrupted by grinding under liquid nitrogen, resuspended in 10mM NaN_3_- 10% TCA-20mM N-ethylmaleimide, and kept on ice for 30 min. Two volumes of cold acetone (-20°C) was added, and after 30 min incubation on ice the samples were centrifuged at 14 000 g, +4°C, for 10 minutes. The pellets were washed with -20°C acetone and resuspended (30 min, room temperature) in sample buffer (7M urea, 2M thiourea, 15mM Tris base, 4% w/v CHAPS). In order to remove insoluble material, the samples were centrifuged 14 000 g at +4°C for 10 min. The pH in the samples was adjusted to pH 8.5, and the protein concentration was adjusted to 7mg/ml. The 2-D Quant Kit (GE Healthcare) was used to measure protein concentration in the samples, according to the instructions of the manufacturer. Three replicate protein extracts were prepared for each of the cultivations.

For 2D gel electrophoresis, three replicate protein extracts from each cultivation were labelled with CyDye DIGE Cy5 (GE Healthcare) as instructed by the manufacturer. A mixture containing equal amounts of all the samples was labelled with CyDye DIGE Cy3 and used as an internal standard in the analysis (GE Healthcare). 50 *μ*g of the Cy5 labelled protein samples mixed with an equal amount of the Cy3 labelled internal standard were subjected to isoelectric focusing using immobilised pH gradients Immobiline DryStrip pH 4-7 or pH 3-5.6 and pH 5.3-6.5 (GE Healthcare) and the IPGphor equipment (GE Healthcare) according to the instructions of the manufacturer. The isoelectric focusing was followed by 11% w/v SDS-PAGE as the second dimension.

The 2D gels were scanned independently for Cy5 and Cy3 labelled proteins using Typhoon 8600 scanner (GE Healthcare), and the image analysis was done using Progenesis software (Nonlinear Dynamics). The ratiometric method provided by the Progenesis software (based on the ratio of the Cy5 signal to the Cy3 signal of the spot in the same gel) was used for normalisation of the signals in the 2D gels. The normalised intensities of the protein spots in the replicate protein extracts of the cultivation were averaged and the average values used in Student's t-test to evaluate the significance of the difference between the groups of cultivations.

Protein spots with more than 2-fold differences (t-test *p <*0.05) in the intensity between the cultivations were selected to LC-MS/MS for identification. The gel spots were excised manually and stored in -20°C. Before LC-MS/MS analysis, the gel spots were destained as instructed by manufacturer and trypsin digested as described earlier [106]. Protein digests were desalted and concentrated with ZipTip *μ*-C18 reverse phase columns (Millipore Corporation). The eluent was evaporated in vacuum centrifuge and peptides dissolved in 0.1% HCOOH and then subjected to automated nanoLC-MS/MS. The used instruments and methods were as in [107]. Obtained spectral data was analysed by in-house Mascot software (Matrix Science) against *T. reesei *genome sequence version 1.2 [104].

### Data analysis

#### Analysis of TRAC data

In order to correlate TRAC and microarray data different linear models were tested and the best selected based on Akaike Information Content. The three best models are shown as scatter plots in Additional file 1, Figure S2, along with diagnostic plots for the best model. m0 is a model with single intercept and slope, m1 a model with gene specific intercept and m2 a model with gene specifc slope. m1 had the lowest AIC and was thus selected:

$$Array_{ij}=\alpha_{j}+\beta *TRAC_{ij}+\varepsilon_{ij}\text{ and }\varepsilon_{ij}\sim N\left(0,\sigma^{2}\right)$$

Where Array is the microarray signal of *j*th gene in the *i*th sample, TRAC is respectively the TRAC signal, *α *the gene specific intercept, *β *the slope (estimated as 0.52) and *σ *the residual standard error (estimated as 0.25 with R package nlme [108]).

#### Gene annotation

Functional annotation of genes was based on [5] and included Interpro protein domain prediction [109], Protfun protein function prediction [110] and TargetP protein localisation prediction [111].

Protein clusters from [5], updated to 49 species [112], were used to transfer annotation between species, namely from the Saccharomyces Genome Database [113] and MIPS Funcat [114], provide a taxonomic specificity for each *T. reesei *gene and construct phylogenetic trees as in [112] with TNT [115]. Taxonomic specificity was defined for each gene by checking from each protein cluster whether it contains only Trichoderma or Pezizomycota genes or is found more generally in Fungi.

Carbohydrate Active enZYme database [116,117] predictions were obtained from [14].

Based on these computationally created annotations and literature searches, genes with significant changes or significant correlation were also manually categorised in a two level hierarchy of 'Class' and 'Extension'. Metabolic model for *T. reesei *(Additional file 2) was contructed by mapping *T. reesei *genes to *Aspergillus niger *genes by bi-directional best hit blastp [118] with a cut-off of bit score *>*50 in either direction ). Metabolic reactions were then transferred for each gene from the *A. niger *metabolic model [48]. Homology relationships of genes of central carbon metabolism were further verified by constructing a phylogenetic tree for each protein cluster which contained several genes from *Saccharomyces cerevisiae *or *Trichoderma reesei*. Annotation of the fungal secretion system was retrieved from [119]. Genes were mapped to protein clusters from [5] and from them to *T. reesei*.

Gene names in capitals are derived from the *S. cerevisiae *according to Saccharomyces Genome Database [113], while names in italics are from other fungal species as specified. Numbers after gene names or descriptions refer to *T. reesei *genome version 2.0 gene identifiers.

#### Analysis of transcriptome data

All data analysis was carried out with R [120] and Bioconductor [121]. The raw array data obtained from Nimblegen was first normalised with RMA (Robust Multichip Average) [122] and LIMMA (Linear Models for Microarrays) [123] was subsequently used to select significantly changing genes with a cut-off of p-value *<*0.05 (which corresponds to a false-discovery rate of 5% in this analysis) and log2 fold-change *>*0.5.

For each gene its correlation to specific protein production was calculated. The false discovery rate was estimated from the Q-value [124] using the R package 'qvalue', and found to be 3.3% for absolute correlation *>*0.8 and 4.8% for *>*0.7. The probability of observing a pair of adjacent genes both with absolute correlation *>*0.7 and the same direction of correlation, was estimated with a permutation test and found to be *p <*1*e *- 5.

Genomic clusters were found by looking for three adjacent genes with absolute correlation *>*0.7 with the same direction of correlation, and then extending the cluster to adjacent genes untill the direction of correlation changed.

Conservation of chromosomal clusters was studied by looking for combinations of Interpro domains found in the genes of the genomic cluster in other genomes. Protein sequences of 33 fungi [5] were mapped to their respective genome sequences by tblastn [118] in order to find genomic co-ordinates for each gene. From the gene co-ordinates, windows of 16 or 30 consecutive genes along chromosomal sequence were calculated, moving the window along the chromosome with increments of 2 or 5 genes, respectively, to cover all 33 genomes with overlapping windows. For each window its protein domain content, i.e. InterPro identifier content, was determined, based on protein domains of individual genes. We then searched for windows in which Interpro domain content overlapped that of the *T. reesei *genomic clusters found above and verified that the genes in these windows were either ortho- or paralogoues using protein clusters from [5].

Gene enrichment tests were carried out with a hypergeometric test for computationally created annotations and with a Ficher test for manual annotations, both with a cut-off of *p <*0.05.

Significance of difference of general characterics (GC%, fold change, etc.) of genes significantly positively or negatively correlated to SPPR from non-correlated genes was checked with a linear model.

Metabolic pathways correlated with specific protein production rate were identified with EMPath as in [47] using the color coding algorithm [125]. The p-value of correlation to specific exracellular protein production rate was assigned to node (gene) weights of the metabolic network. Edge (metabolite linking genes) were weighted with the probability of observing such an edge i.e. by the product of the bi-directional blast hit scores from *A. niger *versus *T. reesei *homology search used to construct the model.

Path lengths from 3 to 12 were investigated. The p-value of a path was estimated from an empirical distribution of shuffling the edge and node weights 10 000 times and the overlapping paths with a p-value below the cut-off, combined into the final result paths. Reporter metabolites [46] were identified using the same weight settings in gene nodes of the metabolic network.

## Authors' contributions

MA prepared and submitted RNA for microarray analysis, carried out transcriptomics data analysis and drafted the manuscript. JR carried out TRAC experiments and their preliminary data analysis. MW, JR and BS carried out bioreactor cultivations and their data analysis. TP carried out proteomics analysis and its data analysis. HK carried protein identifications. PJ carried out Reporter Metabolite analysis. EL carried out EMPath analysis. MW, TP, MS and MP conceived the study and participated in the design of the study. All authors read and approved the final manuscript.

## Supplementary Material

Additional file 1 **Supplementary Figures and Tables**. Click here for file

Additional file 2 **Metabolic model**. Excel file of the mapping of *T. reesei *genome to the *A. niger *metabolic model including enzymes named by phylogenetic analysis for Figure 3. Click here for file
