# Supplementary material for: Correlation of gene expression and protein production rate - a system wide study
Source: BMC Genomics. 2011 Dec 20;12:616. doi: 10.1186/1471-2164-12-616 (PMC3266662; doi:10.1186/1471-2164-12-616)
Supplement: Additional file 1 — Supplementary Figures and Tables. [file 1471-2164-12-616-S1.PDF]

Supplementary data to 'Correlation of gene expression and protein production rate - a system wide study'

Mikko Arvas*[1], Tiina Pakula[1], Bart Smit[2], Jari Rautio[3], Heini Koivistoinen[4], Paula Jouhten[1], Erno Lindfors[1], Marilyn Wiebe[1], Merja Penttilä[1] and Markku Saloheimo[1]

[1]VTT Technical Research Centre of Finland, Tietotie 2, P.O. Box FI-1000, 02044 VTT, Espoo, Finland
[2]NIZO food research, Kernhemseweg 2, 6718ZB Ede, the Netherlands
[3]Plexpress, Helsinki, Viikinkaari 6, 00790 Helsinki, Finland
[4]Ypap Oy, Hyrsynkulmantie 68, FI-32100 Ypäjä, Finland

Email: Mikko Arvas*- mikko.arvas@vtt.fi; Tiina Pakula - tiina.pakula@vtt.fi; Bart Smit - basmit@gmail.com; Jari Rautio - jari.rautio@plexpress.fi; Heini Koivistoinen - heini.koivistoinen@gmail.com; Paula Jouhten - paula.jouhten@vtt.fi; Erno Lindfors - erno.lindfors@vtt.fi; Marilyn Wiebe - marilyn.wiebe@vtt.fi; Merja Penttilä - merja.penttila@vtt.fi; Markku Saloheimo - markku.saloheimo@vtt.fi;

*Corresponding author

Supplementary Figures

Figure 1 - Workflow of the data analysis process

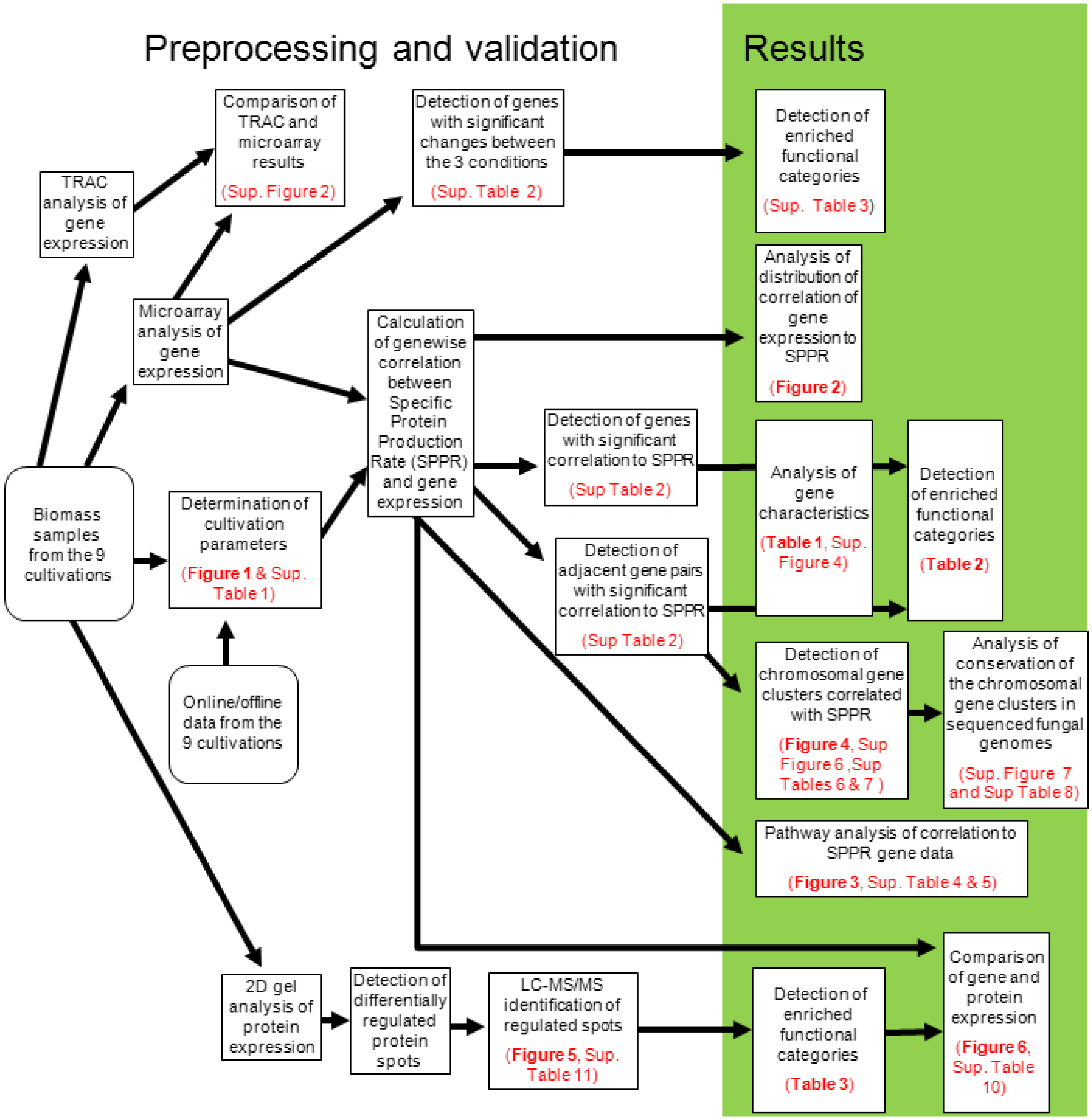

Preprocessing and validation
Results
TRAC analysis of gene expression
Comparison of TRAC and microarray results (Sup. Figure 2)
Detection of genes with significant changes between the 3 conditions (Sup. Table 2)
Detection of enriched functional categories (Sup. Table 3)
Microarray analysis of gene expression
Calculation of genewise correlation between Specific Protein Production Rate (SPPR) and gene expression
Analysis of distribution of correlation of gene expression to SPPR (Figure 2)
Detection of genes with significant correlation to SPPR (Sup Table 2)
Analysis of gene characteristics (Table 1, Sup. Figure 4)
Detection of enriched functional categories (Table 2)
Biomass samples from the 9 cultivations
Determination of cultivation parameters (Figure 1 & Sup. Table 1)
Detection of adjacent gene pairs with significant correlation to SPPR (Sup Table 2)
Detection of chromosomal gene clusters correlated with SPPR (Figure 4, Sup Figure 6 ,Sup Tables 6 & 7)
Analysis of conservation of the chromosomal gene clusters in sequenced fungal genomes (Sup. Figure 7 and Sup Table 8)
Online/offline data from the 9 cultivations
Pathway analysis of correlation to SPPR gene data (Figure 3, Sup. Table 4 & 5)
Comparison of gene and protein expression (Figure 6, Sup. Table 10)
2D gel analysis of protein expression
Detection of differentially regulated protein spots
LC-MS/MS identification of regulated spots (Figure 5, Sup. Table 11)
Detection of enriched functional categories (Table 3)

Figure 1:

Figure 2 - Correlation between TRAC and microarray expression signal

The three panels on the left are identical scatterplots of array signal on the X axis and Trac signal on the Y axis. Signals are averages over biological and technical repeats i.e. there is one signal for each of the three conditions. All the three points of a gene are in same color, but several genes might have the same color. On each scatterplot a different linear regression model is superimposed as black line(s) and the Akaike information criterion (AIC) of the model is shown as title of the plot. In model m1 and m2 a gene specific model is fitted thus the black line spans the three data points of a single gene. Further diagnostic plots are shown on the left for the model with the lowest AIC i.e. m1 On top a histogram of array signals, in middle the residuals versus fitted plot and at the bottom the quantile-quantile plot.

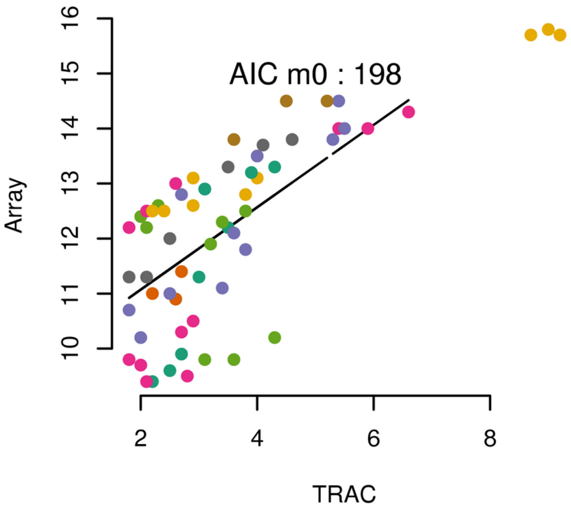
AIC m0 : 198
Array
TRAC
16
15
14
13
12
11
10
2
4
6
8

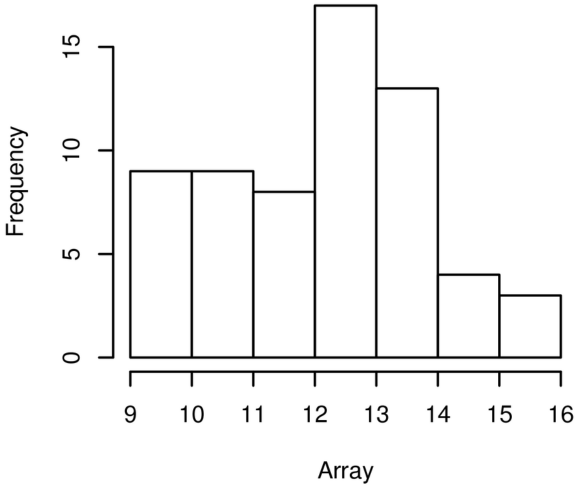
Frequency
Array
0
5
10
15
9
10
11
12
13
14
15
16

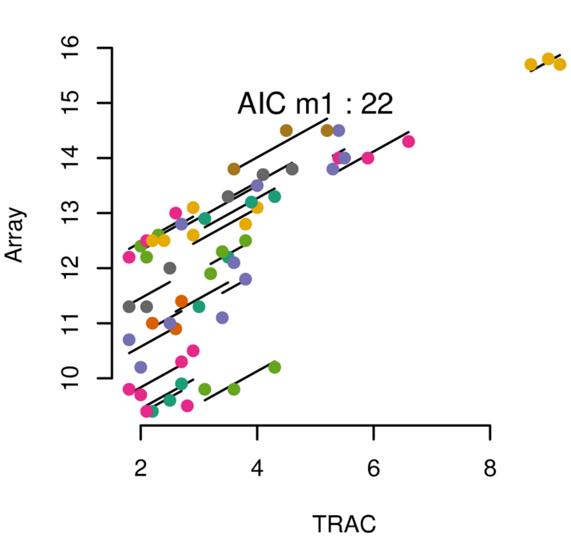
AIC m1 : 22
Array
TRAC
16
15
14
13
12
11
10
2
4
6
8

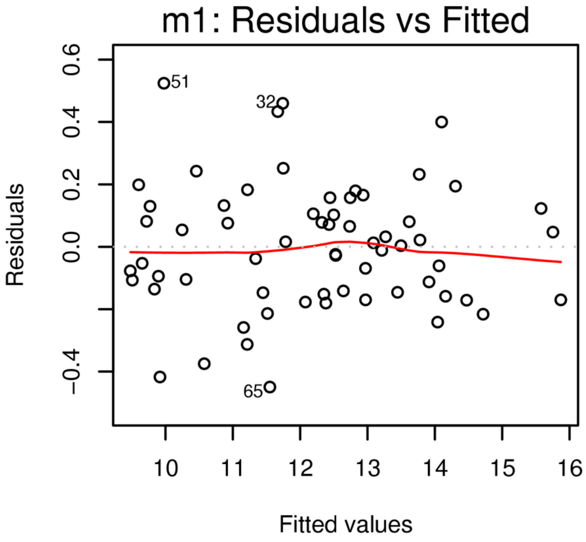
m1: Residuals vs Fitted
Residuals
Fitted values
51
32
65
0.6
0.4
0.2
0.0
−0.4
10
11
12
13
14
15
16

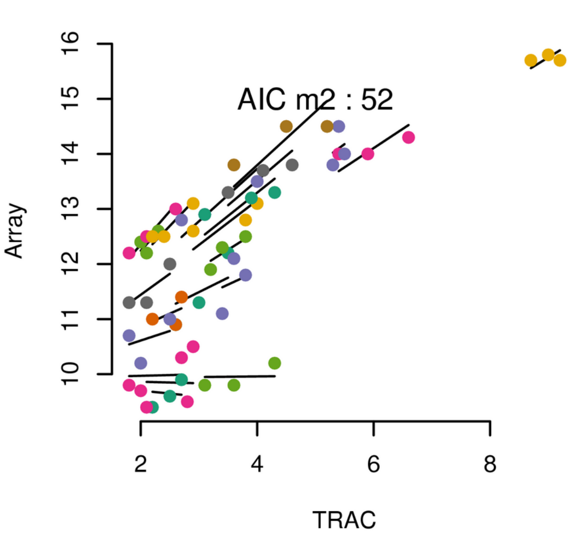
AIC m2 : 52
Array
TRAC
16
15
14
13
12
11
10
2
4
6
8

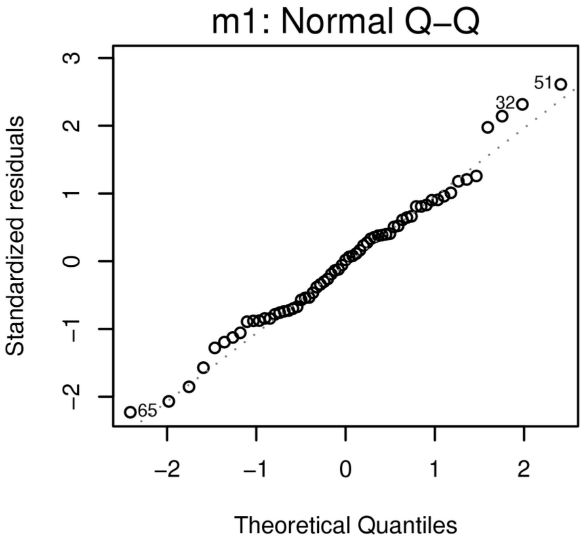
m1: Normal Q−Q
Standardized residuals
Theoretical Quantiles
51
32
65
3
2
1
0
−1
−2
−2
−1
0
1
2

Figure 3 - Comparison of gene wise correlations to SPPR calculated from full data and data lacking cultivation F20

The Y axis shows the correlation to SPPR calculated from data where cultivation F20 was excluded and the X axis correlation to SPPR from full data for each gene. Genes plotted in red have correlation above abs(0.8) in full data i.e. they are discussed in the paper. Of the 491 with positive correlation with full data, 484 have a correlation above 0.8 also in F20 excluded data set, 477 and 470 respectively for negative correlation. Using F20 excluded data set there are 689 genes with correlation above 0.8 and 676 respectively for negative correlation. Pearson correlation between correlations to SPPR between the full and F20 excluded data sets is 0.995.

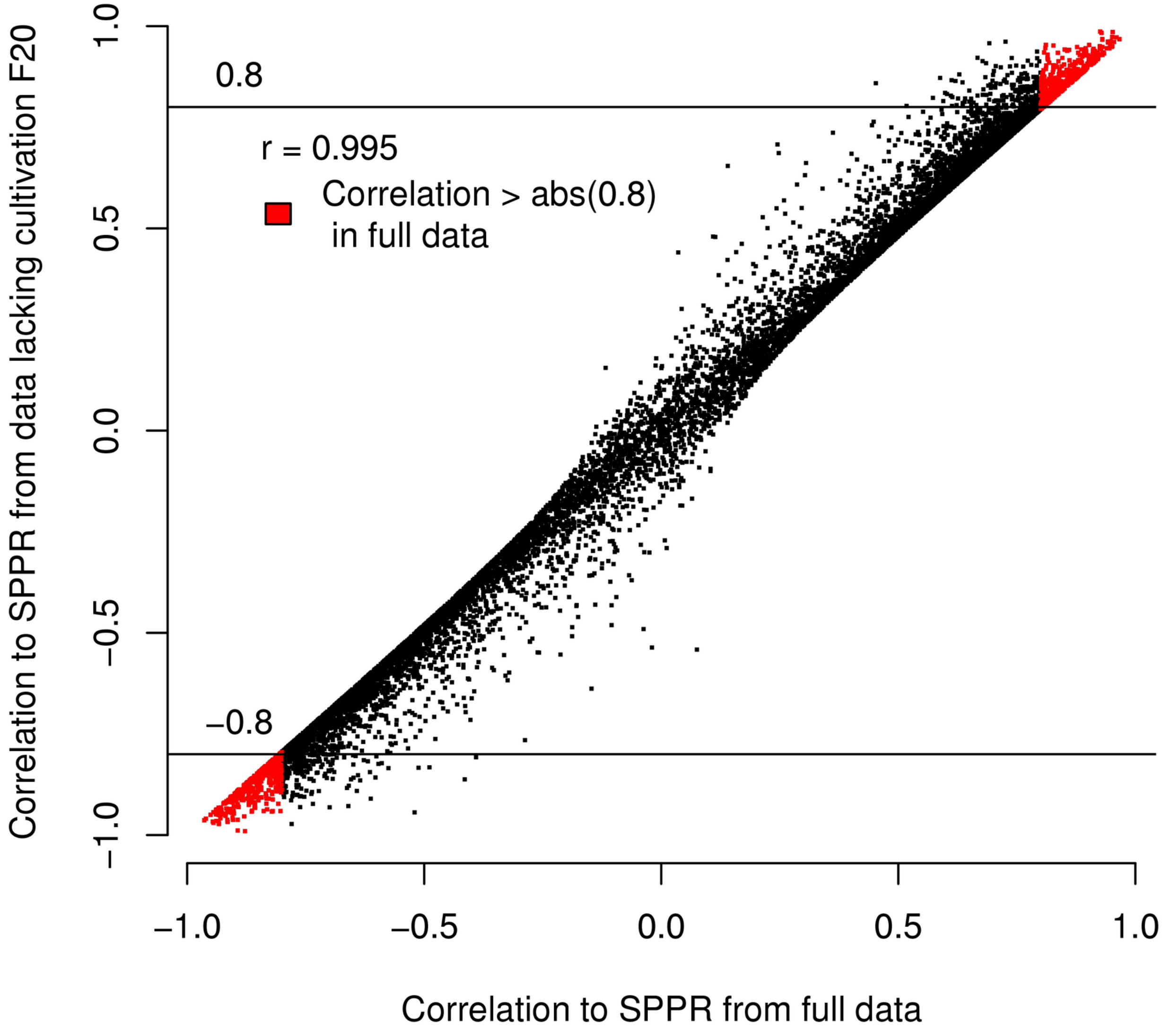

0.8
r = 0.995
Correlation > abs(0.8)
in full data
−0.8
1.0
0.5
0.0
−0.5
−1.0
Correlation to SPPR from data lacking cultivation F20
−1.0
−0.5
0.0
0.5
1.0
Correlation to SPPR from full data

Figure 3:

Figure 4 - Boxplot of general gene characteristics

The Y axis is specified on the title of each plot. On the X axis genes are categorised according to their correlation with specific protein production rate. First of the four digits specifies that the genes belong to C07wN, and rest of the digits respectively specify membership of - C07wN, C08 and -C08. For example '1010' means that the genes belong to a pair of chromosomally adjacent genes which both have correlation with specific protein production rate above 0.7 and the genes have correlation with specific protein production rate above 0.8. Categories that include -C08 genes are highlighted in red.

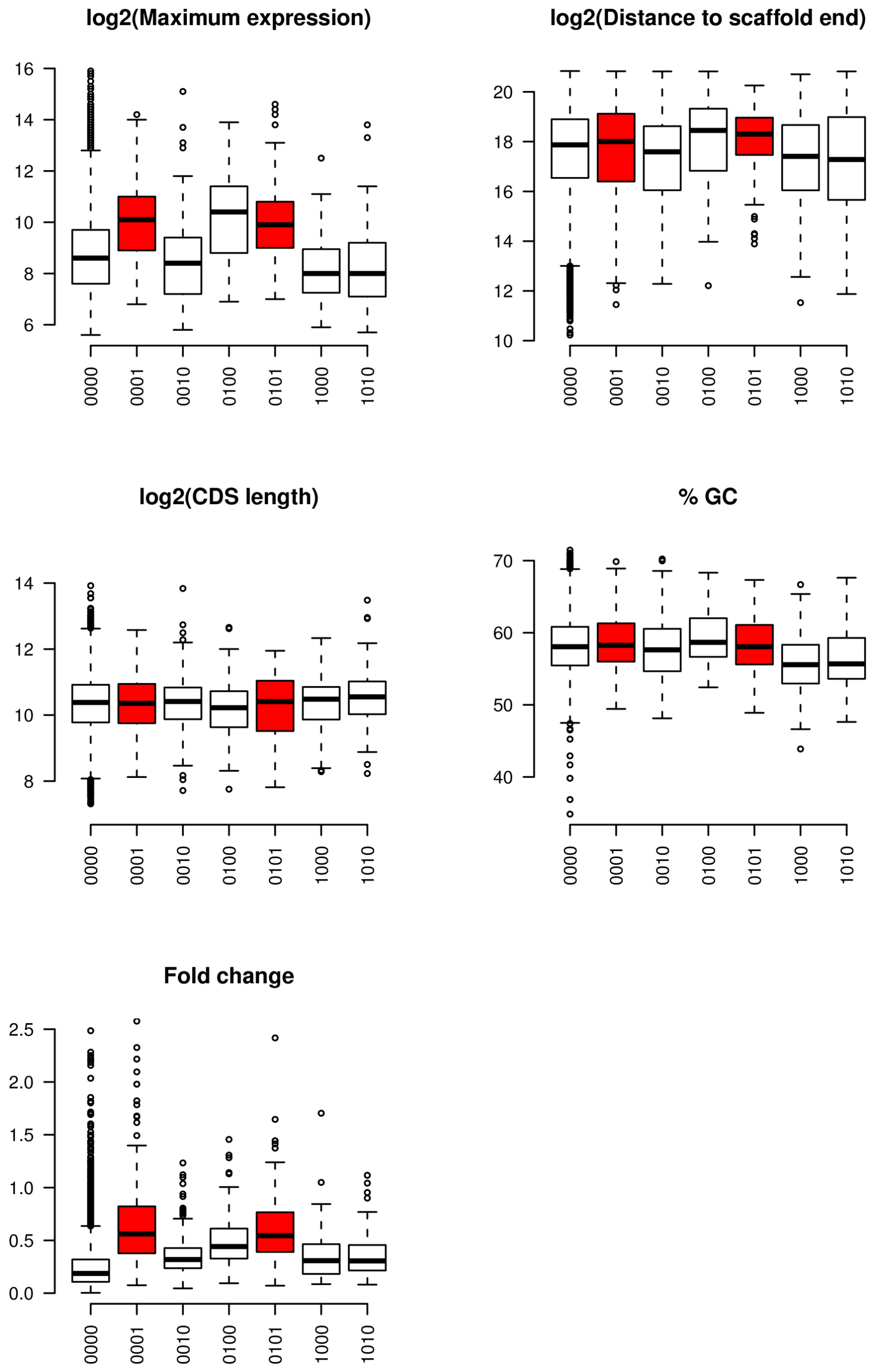
log2(Maximum expression)
log2(Distance to scaffold end)
log2(CDS length)
% GC
Fold change
0000
0001
0010
0100
0101
1000
1010

Figure 4:

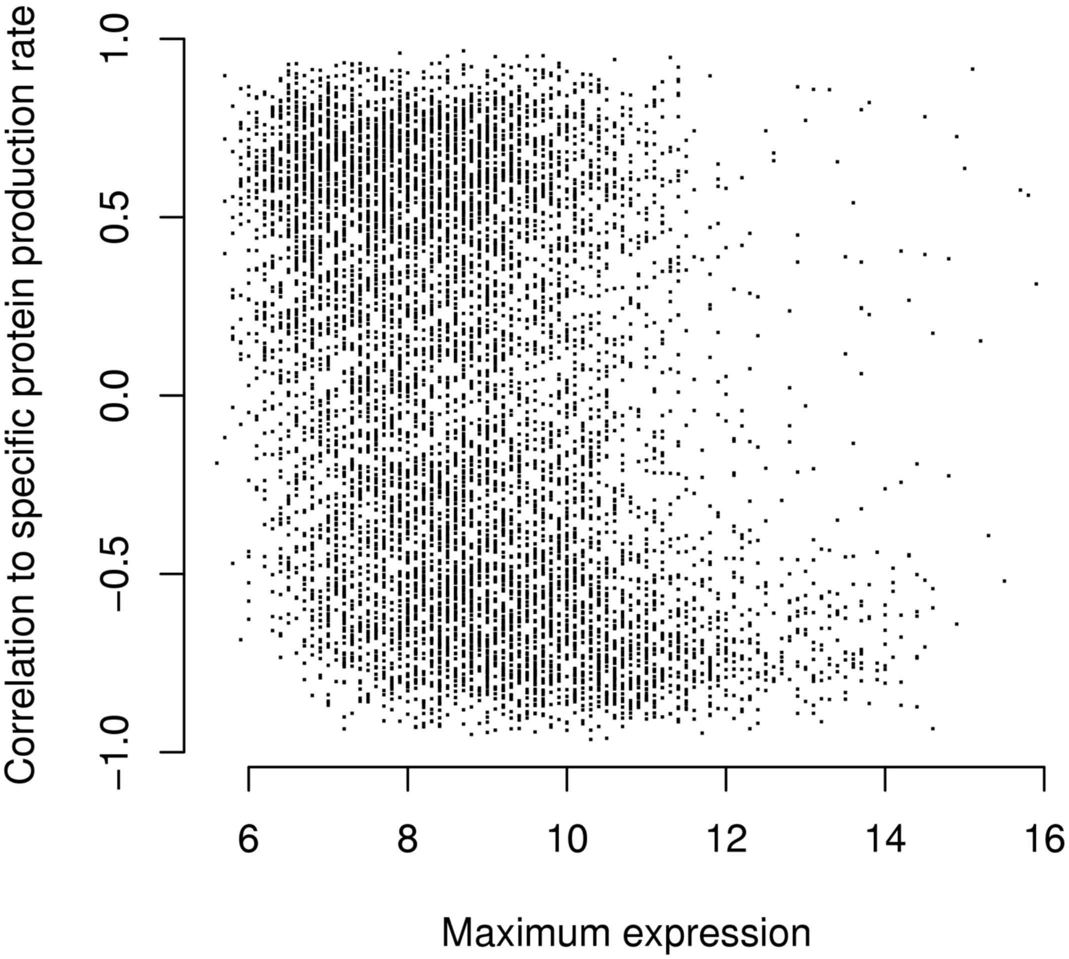

Figure 5:

Figure 5 - Scatterplot of specific protein production rate and maximum expression

The Y axis shows the correlation to specific protein production rate and X axis the maximum expression in the whole experiment for each gene.

Figure 6 - Cromosomal gene clusters with negative correlation to specific protein production rate on scaffolds

See main paper Figure 4 for legend.

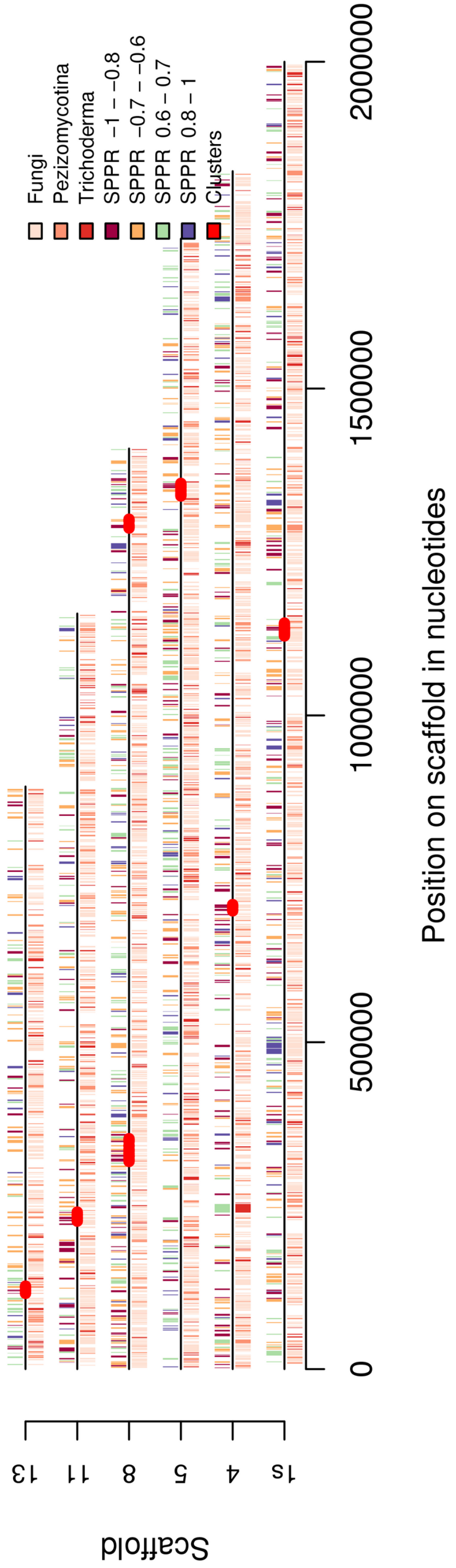
Fungi
Pezizomycotina
Trichoderma
SPPR −1 − −0.8
SPPR −0.7 − −0.6
SPPR 0.6 − 0.7
SPPR 0.8 − 1
Clusters
Position on scaffold in nucleotides
0
500000
1000000
1500000
2000000
Scaffold
13
11
8
5
4
1s

Figure 6:

Figure 7 - Comparison of negatively correlating gene cluster 7 (cn7 in Supplementary Table 8) between fungal species

A stretch of scaffold from seven fungal species containing the cluster and neighbouring genes. Left to each strech an abreviation for the fungal species and the scaffold identifier (Fgra: *Fusarium graminearum*; TrRee: *Trichoderma reesei*; Cglo: *Chaetomium globosum*; *Ncra: Neurospora crassa*; *Afum: Aspergillus fumigatus*; Anid: *Aspergillus nidulans*; Cimm: *Coccidioides immitis*). The X axis specifies the position relative to start and the start and end co-ordinates of each stretch on the scaffold are shown at its beginning and end, respectively. Genes are shown as boxes on the scaffold stretch. Homologues of the genes of actual cluster 7 (CZ, UN1, WD, bet3 and OefA) are shown in red. Interpro protein domain identifiers are shown under each gene excluding leading IPR0 i.e. 01878 is IPR001878. Grey lines connect genes with identical protein domains, excluding domains found in the genes coloured red in order to reveal syntenies between the scaffolds. Full scaffold, gene and protein domain names can be found from supplementary data Table 8.

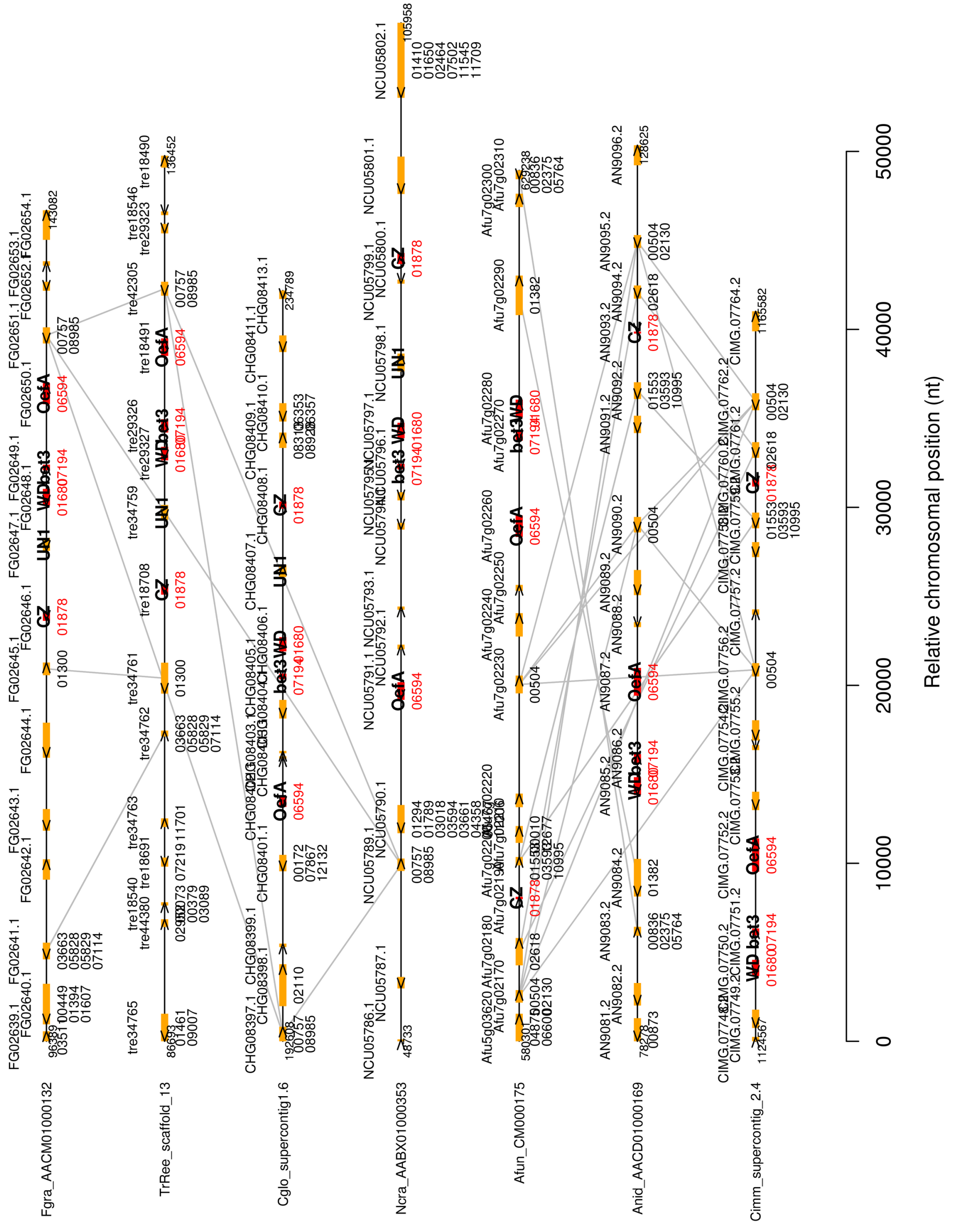
Fgra_AACM01000132
TrRee_scaffold_13
Cglo_supercontig1.6
Ncra_AABX01000353
Afun_CM000175
Anid_AACD01000169
Cimm_supercontig_2.4
0
10000
20000
30000
40000
50000
Relative chromosomal position (nt)

Figure 7:

Supplementary Tables

Table 1 - Characteristics of the chemostat cultivations.

Cultivation	Type	Specific growth rate 1/h	Specific protein production rate mg/g h	Specific CBHI production rate μkat/g h	Specific EGI production rate μkat/g h	Specific lactose consumption rate g/g h	Specific ammonium sulphate consumption rate g/g h	Yield of biomass g/g	Yield of extracellular protein g/g	Dry weight g/l
F20	D03	0.031	5.11	0.006	0.013	0.06	0.017	0.42	2.29	4.2
F21	D03	0.031	6.88	0.007	0.016	0.08	0.022	0.32	2.31	3.2
F23	D06	0.058	4.62	0.004	0.007	0.11	0.025	0.44	3.04	3.8
F25	D03	0.031	7.50	0.004	0.015	0.07	0.021	0.35	2.22	3.5
F26	D06	0.058	4.58	0.002	0.009	0.12	0.027	0.41	2.82	4.0
F27	D06	0.057	4.42	0.001	0.009	0.11	0.026	0.43	2.80	4.1
F30	HD	0.031	1.07	0.002	0.003	0.08	0.015	0.36	5.27	14.2
F31	HD	0.030	1.60	0.005	-0.001	0.08	0.015	0.35	5.21	13.9
F32	HD	0.032	1.55	0.004	0.001	0.08	0.015	0.37	5.25	14.4

Table 2 - Counts of significant genes

Counts of significantly different genes detected by Limma and analysis of correlation of gene expression to specific protein production rate. Column 'Direction' shows if gene expression was higher in first condition of a contrast (1), second condition (-1) or not significant (0), or if correlation was positive (1), negative (-1) or not significant (0). Column 'Test' shows the test: Limma (L), correlation above 0.8 or below -0.8 (C08) or correlation of two chromosomally nearest neighbouring genes both above 0.7 or below -0.7 (C07wN). Column 'Total count' shows the count of genes and subsequent columns breakdown by contrast. For example of the 411 genes that were significant in C07wN, 224 had a positive correlation and 187 negative, of these 30 were significantly higher in D03 than in HD and 95 in HD than in D03.

Direction	Test	Total count	D03/D06	D03/HD	D06/HD
1	L		10	203	97
0	L	9559	9519	8620	9087
-1	L		30	736	375
1	C08	477	4	74	17
0	C08	8592	959	616	813
-1	C08	490	4	277	137
1	C07wN	224	2	30	8
0	C07wN	9148	408	286	366
-1	C07wN	187	1	95	37

Table 3 - Enriched functional categories of differentially regulated genes between cultivations

'ID' is InterPro or Funcat identifier or is blank for authors' manual annotation. 'Description' is short name for the category. 'Expression' is type of expression behaviour the genes have. For each gene significant difference of expression between the three different conditions (D03, D06 and HD) was tested by three different contrasts (D03/D06, D03/HD and D06/HD) i.e. D03/HD means that the genes in the set were higher in D03 than in HD and -D06/HD that the genes were higher in HD than in D06. '% in expression' is the percentage of genes among the group of genes with same expression that belong to the annotation category. '% of annotated' is the percentage of genes among all genes that belong to the annotation category. For authors' manual annotations this is the percentage of genes with the same annotation among the group of genes with opposite expression behaviour. 'p-value' is for the significance of enrichment. 'Intepretation' is a biological concept to which the genes relate to as intepreted by the authors.

ID	Description	Expression	% of expressed	% of annotated	p-value	Intepretation
IPR003663	Sugar/inositol transporter	D03/D06	30.0	6.3	0.000	Transporter
20.09.04	mitochondrial transport	-D03/D06	10.3	5.4	0.009	Mitochondrial
Class	Secreted	D03/HD	11.3	1.5	0.000	Secreted
IPR004841	Amino acid permease domain	D03/HD	3.6	18.8	0.000	Transporter
Class_Extension	Secreted Transmembrane	D03/HD	3.8	0.0	0.000	Transporter
Class_Extension	Secreted Unknown	D03/HD	7.5	1.3	0.000	Secreted
Class	Glycoside hydrolase	D03/HD	9.4	2.4	0.000	Secreted
IPR011701	Major facilitator superfamily MFS-1	D03/HD	5.5	5.9	0.003	Transporter
IPR002085	Alcohol dehydrogenase superfamily, zinc-containing	D03/HD	3.0	9.8	0.003	2ndary metabolism?
Class_Extension	Metabolism SDR	D03/HD	1.9	0.0	0.007	2ndary metabolism?
Class_Extension	Unknown Unknown	D03/HD	15.1	8.9	0.027	Unknown
IPR000073	Alpha/beta hydrolase fold-1	D03/HD	1.8	7.7	0.041	2ndary metabolism?
Class_Extension	Metabolism 2ndary	D03/HD	2.5	0.6	0.047	2ndary metabolism?
12	PROTEIN SYNTHESIS	-D03/HD	10.8	29.6	0.000	Protein synthesis
12.01.01	ribosomal proteins	-D03/HD	5.1	34.3	0.000	Protein synthesis
IPR001353	Proteasome, subunit alpha/beta	-D03/HD	0.8	42.9	0.001	Protein degradation
IPR001804	Isocitrate/isopropylmalate dehydrogenase	-D03/HD	0.6	66.7	0.001	Primary metabolism
IPR002423	Chaperonin Cpn60/TCP-1	-D03/HD	0.7	50.0	0.001	Cytoskeleton
IPR010929	CDR ABC transporter	-D03/HD	0.6	57.1	0.001	Transporter
12.04.01	translation initiation	-D03/HD	1.7	44.4	0.002	Protein synthesis
12.01	ribosome biogenesis	-D03/HD	5.8	28.9	0.002	Protein synthesis
IPR001023	Heat shock protein Hsp70	-D03/HD	0.7	41.7	0.002	Protein synthesis
02	ENERGY	-D03/HD	7.7	26.8	0.002	Energy
12.04	translation	-D03/HD	2.8	35.1	0.003	Protein synthesis
IPR000717	Proteasome component (PCI) domain	-D03/HD	0.7	38.5	0.003	Protein degradation
Class	Protein transport	-D03/HD	0.6	5.8	0.003	Protein transport
IPR005225	Small GTP-binding protein	-D03/HD	1.3	23.7	0.004	Protein transport
IPR000626	Ubiquitin	-D03/HD	0.7	35.7	0.004	Protein degradation
IPR011761	ATP-grasp fold	-D03/HD	0.6	44.4	0.005	Primary metabolism
IPR000217	Tubulin	-D03/HD	0.4	60.0	0.005	Cytoskeleton
IPR002078	RNA polymerase sigma factor 54, interaction	-D03/HD	0.4	60.0	0.005	Protein transport
IPR002108	Actin-binding, cofilin/tropomyosin type	-D03/HD	0.4	60.0	0.005	Cytoskeleton
Class	Mitochondrial	-D03/HD	1.9	7.0	0.014	Mitochondrial
IPR003439	ABC transporter-like	-D03/HD	1.4	18.2	0.016	Transporter
IPR001452	Src homology-3 domain	-D03/HD	0.8	24.0	0.016	Cytoskeleton
IPR002194	Chaperonin TCP-1, conserved site	-D03/HD	0.4	42.9	0.016	Cytoskeleton
IPR001969	Peptidase aspartic, active site	-D03/HD	0.6	30.8	0.020	Protein degradation
02.07	pentose-phosphate pathway	-D03/HD	0.8	46.2	0.023	Primary metabolism
IPR003593	ATPase, AAA+ type, core	-D03/HD	2.1	14.7	0.024	Various
IPR002889	Carbohydrate-binding WSC	-D03/HD	0.4	37.5	0.024	Secreted
IPR000008	C2 calcium-dependent membrane targeting	-D03/HD	0.6	28.6	0.026	Various
01.03.01.03	purine nucleotide anabolism	-D03/HD	0.6	57.1	0.027	Primary metabolism
12.10	aminoacyl-tRNA-synthetases	-D03/HD	1.7	32.4	0.034	Protein synthesis
IPR000449	Ubiquitin-associated/translation elongation factor EF1B, N-terminal	-D03/HD	0.4	33.3	0.034	Various
IPR001202	WW/Rsp5/WWP	-D03/HD	0.4	33.3	0.034	Various
IPR004000	Actin/actin-like	-D03/HD	0.4	33.3	0.034	Cytoskeleton
42.04	cytoskeleton	-D03/HD	3.0	27.6	0.038	Cytoskeleton
02.10	tricarboxylic-acid pathway (citrate cycle, Krebs cycle, TCA cycle)	-D03/HD	1.1	36.4	0.041	Primary metabolism
01.01.06.04	metabolism of threonine	-D03/HD	0.6	50.0	0.046	Primary metabolism
IPR004365	Nucleic acid binding, OB-fold, tRNA/helicase-type	-D03/HD	0.4	30.0	0.046	Protein synthesis
Class	DNA	D06/HD	6.5	0.6	0.003	Chromatin
IPR001395	Aldo/keto reductase	D06/HD	3.8	9.4	0.003	2ndary metabolism?
Class_Extension	Secreted Transmembrane	D06/HD	3.9	0.0	0.006	Transporter
IPR000182	GCN5-related N-acetyltransferase	D06/HD	3.8	7.1	0.007	Chromatin
IPR002347	Glucose/ribitol dehydrogenase	D06/HD	5.0	3.4	0.025	2ndary metabolism?
Class_Extension	Unknown Unknown	D06/HD	20.8	11.4	0.039	Unknown
Class	Unknown	D06/HD	28.6	17.9	0.040	Unknown
Class	Secreted	D06/HD	10.4	4.1	0.042	Secreted
IPR010929	CDR ABC transporter	-D06/HD	0.8	42.9	0.002	Transporter
IPR001452	Src homology-3 domain	-D06/HD	1.4	20.0	0.004	Cytoskeleton
IPR002889	Carbohydrate-binding WSC	-D06/HD	0.8	37.5	0.004	Secreted
02.07	pentose-phosphate pathway	-D06/HD	1.4	38.5	0.005	Primary metabolism
IPR002772	Glycoside hydrolase, family 3, C-terminal	-D06/HD	0.8	27.3	0.010	Secreted
IPR005225	Small GTP-binding protein	-D06/HD	1.4	13.2	0.021	Protein transport
IPR004843	Metallophosphoesterase	-D06/HD	1.1	14.3	0.029	Various

Table 4 - Results of reporter metabolite analysis by metabolite

Name of the metabolite and an abbreviation for it, p-value in reporter metabolite analysis, count of genes adjacent to the metabolite in metabolic network and respectively count of different enzymes adjacent to the metabolite.

Metabolite	Metabolite abbr.	p-value	Adjacent genes	Adjacent enzymes
5,10-Methenyltetrahydrofolate	METHF	0.02	2	2
alpha-D-Glucose 6-phosphate	G6P	0.02	5	5
5-Phosphoribosylamine	PRAM	0.02	1	1
UDP-N-acetyl-D-glucosamine	UDPNAG	0.02	5	1
3-(4-Hydroxyphenyl)pyruvate	4HPP	0.03	4	2
D-Mannose	MAN	0.03	2	1
D-Glucosamine	GLCN	0.03	3	2
protein-dihydrolipoyllysine	DHPLYSm	0.04	1	1
tetrahydrofolate	THFm	0.04	3	2
O-Phospho-L-homoserine	PHSER	0.04	1	1
(thiamine diphosphate)-alpha-ketoglutarate dehydr.	TDPE1m	0.05	1	1
alpha-ketoglutarate bound to alpha-ketoglutarate dehyd.	AKGE1m	0.05	2	2

Table 5 - Results of reporter metabolite and pathway detection analysis by gene

Gene ID, detection methods (reporter metabolite RM or pathway detection PD or both), homologous gene in *S. cerevisiae*, annotation by authors (Class and Extension), KEGG enzyme name and EC number. Gene expression regulation in the three different contrasts for statistically significant differences between cultivation conditions (D03/D06, D03/HD and D06/HD). 0 stands for no difference, 1 that expression was higher in first condition of a contrast and -1 that is was higher in second condition of a contrast. Correlation to specific protein production rate (Cor2SPPR). Abbrevation of the reporter metabolite (see Supplementary Table 4) and in which of the two paths the gene was detected in pathway detection.

Gene ID	Method	Homologue	Class	Extension	Enzyme name	EC	D03/D06	D03/HD	D06/HD	Cor2SPPR	Reporter metabolite	Path in Pathway detection
5233	RM	AAT2	Metabolism	Amino acid	aspartate transaminase	2.6.1.1	0	-1	0	-0.8	4HPP	
74041	RM		Metabolism	Amino acid	aspartate transaminase	2.6.1.1	0	0	0	-0.4	4HPP	
74725	RM	AAT2	Metabolism	Amino acid	aspartate transaminase	2.6.1.1	0	-1	-1	-0.8	4HPP	
65295	RM, PD	SHM2	Metabolism	Amino acid	glycine hydroxymethyltransferase	2.1.2.1	0	-1	0	-0.9	THFm	
78409	RM	THR4	Metabolism	Amino acid	threonine synthase	4.2.3.1	0	-1	0	-0.8	PHSER	
46545	RM	TYR1	Metabolism	Amino acid	prephenate dehydrogenase (NADP+)	1.3.1.13	0	0	0	-0.9	4HPP	
66541	PD	ICL2	Metabolism	Amino acid	methylisocitrate lyase	4.1.3.30	0	-1	0	-0.9		1
74123	PD	ILV2	Metabolism	Amino acid	acetolactate synthase	2.2.1.6	0	-1	0	-0.8		1
73665	RM, PD	hxk1	Metabolism	Carbohydrate	hexokinase	2.7.1.1	0	-1	-1	-0.9	MAN,GLCN	2
79677	RM	hxk2	Metabolism	Carbohydrate	hexokinase	2.7.1.1	0	0	0	0.5	MAN,GLCN	
21836	RM	PGM1-2	Metabolism	Carbohydrate	phosphoglucomutase	5.4.2.2	0	0	0	-0.7	G6P	
5776	RM, PD	PGI1	Metabolism	Carbohydrate	glucose-6-phosphate isomerase	5.3.1.9	0	-1	-1	-0.9	G6P	2
75769	RM	ZWF1	Metabolism	Carbohydrate	glucose-6-phosphate dehydrogenase	1.1.1.49	0	-1	0	-0.8	G6P	
77602	RM	TPS1	Metabolism	Carbohydrate	alpha,alpha-trehalose-phosphate synthase (UDP-forming)	2.4.1.15	0	0	0	-0.3	G6P	
122385	PD	GFA1	Cell wall	Chitin	glutamine-fructose-6-phosphate transaminase (isomerizing)	2.6.1.16	0	-1	-1	-0.9		2
51492	RM	CHS2	Cell wall	Chitin	chitin synthase	2.4.1.16	0	-1	-1	-0.9	UDPNAG	
55341	RM	CHS2	Cell wall	Chitin	chitin synthase	2.4.1.16	0	0	0	-0.5	UDPNAG	
58188	RM	CHS3	Cell wall	Chitin	chitin synthase	2.4.1.16	0	0	0	-0.5	UDPNAG	
112271	RM	CHS1	Cell wall	Chitin	chitin synthase	2.4.1.16	0	0	0	0.5	UDPNAG	
124228	RM	CHS3	Cell wall	Chitin	chitin synthase	2.4.1.16	0	0	0	0.8	UDPNAG	
81339	RM	GCV2	Metabolism	Folate	aminomethyltransferase, aminomethyltransferase	1.4.4.2	0	0	0	-0.8	DHPLYSm	
75890	RM	MIS1	Metabolism	Folate	methylenetetrahydrofolate dehydrogenase (NADP+)	3.5.4.9	0	-1	0	-0.8	METHF, THFm	
46244	RM	MTD1	Metabolism	Folate	methylenetetrahydrofolate dehydrogenase (NAD+)	1.5.1.15	0	0	0	-0.9	METHF	
79059	RM	INO1	Metabolism	Inositol	inositol-3-phosphate synthase	5.5.1.4	0	-1	0	-0.8	G6P	
54071	PD	QNS1	Metabolism	Nucleotide	NAD+ synthase (glutamine-hydrolyzing)	6.3.5.1	0	-1	0	-0.8		2
22799	PD	ADE6	Metabolism	Nucleotide	phosphoribosylformylglycinamidine synthase	6.3.5.3	0	-1	0	-0.7		2
80872	RM, PD	ADE5,7	Metabolism	Nucleotide	phosphoribosylamine-glycine ligase, phosphoribosylamine-glycine ligase	6.3.3.1	0	-1	0	-0.9	PRAM	2
105072	RM		Metabolism	Polysaccharide deacetylase	N-acetylglucosamine deacetylase	3.5.1.33	0	0	0	0.2	GLCN	
44041	PD	ACP1-like	Mitochondrial	Respiration	NADH dehydrogenase (ubiquinone)	1.6.5.3	0	-1	0	-0.8		1
2745	PD	FUM1	Metabolism	TCA	fumarate hydratase	4.2.1.2	0	-1	0	-0.9		1
121824	PD	ncra_acl1	Metabolism	TCA	ATP citrate synthase	2.3.3.8	0	-1	-1	-0.9		1
77336	PD	ACO1	Metabolism	TCA	aconitate hydratase	4.2.1.3	0	-1	0	-0.8		1
122296	PD	CIT1-2	Metabolism	TCA	citrate (Si)-synthase	2.3.3.1	0	0	0	-0.8		1
3653	RM	KGD2	Metabolism	TCA	dihydrolipoyllysine-residue succinyltransferase	2.3.1.61	0	-1	-1	-0.8	AKGE1m	
50531	RM	KGD1	Metabolism	TCA	oxoglutarate dehydrogenase (succinyl-transferring)	1.2.4.2	0	-1	0	-0.8	TDPE1m, AKGE1m	
121019	PD	SDH1	Metabolism	TCA	succinate dehydrogenase (ubiquinone)	1.3.5.1	0	-1	0	-0.8		1
21758	PD	ICL1	Metabolism	TCA	isocitrate lyase	4.1.3.1	0	-1	-1	-0.9		1

Table 6 - Positively correlating chromosomal gene clusters detected by chromosomal triplets of genes with SPPR over 0.7 **and cluster 'ce1' visible in supplementary Figure 6**

Cluster identifier, gene identifier, strand, scaffold and start and end of the gene. Manual annotation by authors (Class and Extension), additional description and taxonomic specificity (T = Trichoderma, P = Pezizomycotina and F = Fungi) as in main paper Figure 2 of a gene, gene expression regulation as in Table 5 and genes correlation to specific protein production rate.

Cluster	Gene ID	Strand	Scaffold	Start	End	Class	Extension	Description	Taxon	D03/D06	D03/HD	D06/HD	Cor2SPPR
c1	103031	-	1	2406353	2407454	Unknown	Unknown		T	0	0	0	0.7
c1	103032	-	1	2408398	2411706	Metabolism	NRPS-like		F	0	0	0	0.8
c1	103033	+	1	2416227	2416974	Secreted	Unknown		P	0	0	0	0.9
c1	103034	+	1	2417912	2420710	Regulatory functions	Transcription factor	Fungal transcriptional regulatory protein, N-terminal	P	0	0	0	0.7
c1	54972	+	1	2421292	2422906	Transporter	MFS	Major facilitator superfamily	F	0	0	0	0.7
c1	74215	-	1	2423105	2424133	Toxin resistance		RTA1-like	F	0	0	0	0.7
c1	44306	-	1	2425072	2426314	Regulatory functions	Transcription factor	Fungal transcriptional regulatory protein, N-terminal	P	0	1	1	0.9
c1	v1.2_15869	+	1	2434372	2434524	Unknown	Unknown			0	0	0	0.6
c1	29115	-	1	2434650	2435925	Unknown	Unknown		P	0	0	0	0.6
c1	53378	-	1	2437048	2439032	Transporter	MFS	Major facilitator superfamily	F	0	0	0	0.8
c1	103045	+	1	2440405	2441266	Protein degradation	Peptidase S51	Secreted	T	0	0	0	0.6
c2	103045	+	1	2473263	2473319	Unknown	Unknown		T	0	0	0	0.5
c2	2033	+	1	2487538	2489016	Unknown	Unknown		P	0	0	0	0.7
c2	74223	-	1	2489251	2490032	Glycoside hydrolase	Family 11	XYN1, xylanase, secreted	F	0	1	1	0.8
c2	103048	+	1	2492608	2493610	Unknown	Unknown	Unknown	T	0	0	0	0.9
c2	103049	+	1	2495262	2496643	Glycoside hydrolase	Family 28	THPG1, Endo-polygalacturonase, secreted	F	0	1	1	0.9
c2	103050	+	1	2499430	2500116	Glycoside hydrolase	Family 23		T	0	0	0	0.4
c2	53903	-	1	2501770	2503864	Transporter	MFS	Major facilitator superfamily,Aspergillus fumigatus qutD, quinate permease	F	0	0	0	0.4
c3	57045	-	3	44151	45144	Metabolism	Esterase		F	0	0	0	0.6
c3	104064	+	3	46038	46860	Unknown	Unknown		T	0	0	0	0.8
c3	56314	+	3	48357	50225	Transporter	Amino acid permease		F	0	0	0	0.6
c3	57015	-	3	50433	51646	Transporter	Amino acid permease	Aromatic amino acid permease	F	0	0	0	0.7
c3	104067	-	3	54076	55182	Secreted	Glycoside Hydrolase?		P	0	0	0	0.6
c3	56350	-	3	58386	59334	Metabolism	Amino acids	Cysteine synthase	F	0	0	0	0.6
c3	56840	+	3	61027	62005	Metabolism	Oxidoreductase		F	0	0	0	0.7
c3	56684	+	3	63008	64766	Transporter	MFS, Sugar	Sugar transporter	F	1	1	0	0.7
c3	104071	+	3	65211	67367	Unknown	Unknown		P	0	0	0	0.9
c3	104072	+	3	68870	70590	Transporter	MFS, Sugar	TRHXT1, Xylose permease	F	0	0	0	0.3
c3	104073	-	3	70871	71570	Metabolism	2ndary	Aspartate racemace, mainly bacterial homologues	A	0	0	0	0.7
c4	30776	-	7	708719	709804	Metabolism	Taurine oxidoreductase		F	0	0	0	0.8
c4	3532	+	7	711116	712796	Transporter	MFS	Major facilitator superfamily	F	0	0	0	0.7
c4	121405	-	7	713513	715569	Metabolism	Nitrogen, Amino acids	4-aminobutyrate transaminase	F	0	0	0	0.8
c5	60489	+	7	798601	799296	Carbohydrate esterase	Family 5	Cutinase	F	0	1	1	0.9
c5	106479	-	7	799610	800831	Metabolism	Epimerase		F	0	0	0	0.7
c5	106480	-	7	802324	803778	Unknown	FAD-binding		P	0	0	0	0.8
c6	121498	-	7	1378518	1380150	Metabolism	Phospholipids	Phosphatidylserine decarboxylase	E	0	0	0	0.5
c6	121499	+	7	1381169	1382357	Unknown	Unknown		T	0	0	0	0.7
c6	v1.2_18282	-	7	1382791	1382970	Unknown	Unknown			0	0	0	0.7
c6	v1.2_18290	-	7	1382989	1383366	Unknown	Unknown			0	1	0	0.8

c6	41208	+	7	1384838	1385792	Regulatory functions	Protein phosphatase		P	0	0	0	0.7
c6	106706	+	7	1386797	1388847	Regulatory functions	Transcription factor	Fungal transcriptional regulatory protein, N-terminal	T	0	1	0	0.8
c6	60418	-	7	1388977	1390513	Metabolism	Aldehyde dehydrogenase		F	0	0	0	0.4
c6	60374	+	7	1391266	1392944	Metabolism	GMC	Glucose-methanol-choline (GMC) oxidoreductase	F	0	0	0	0.7
c6	59952	+	7	1393990	1395786	Transporter	Amino acid permease		F	0	0	0	0.7
c7	80654	-	19	580777	583600	Glycosyltransferase	Family 1		F	0	0	0	0.7
c7	66657	-	19	584651	586037	Transporter	MFS	Major facilitator superfamily	F	0	0	0	0.5
c7	110311	+	19	587584	588459	Unknown	Unknown		T	0	0	0	0.7
c7	66766	+	19	589077	590030	Metabolism	Nitrilase	Amidohydrolase	F	0	1	1	0.7
c7	123278	-	19	590133	592535	Cell cycle	Incompatibility	Heterokaryon incompatibility domain	P	0	0	0	0.8
c7	123279	+	19	596022	598928	Metabolism	Sulfur	Arylsulfatase	P	0	0	0	0.2
c8	123987	+	29	290485	291559	Unknown	Unknown		T	0	0	0	0.2
c8	v1.2_15293	-	29	292313	292807	Unknown	Unknown			0	0	0	0.8
c8	111827	-	29	293070	293977	Unknown	Unknown		T	0	0	0	0.5
c8	69493	-	29	294320	297033	Glycoside Hydrolase	Family 92	Related to a bacterial a-1,2-mannosidase, secreted	F	0	0	0	0.6
c8	69651	-	29	297692	299492	Transporter	MFS, Sugar	Sugar transporter	F	0	0	0	0.8
c8	69465	-	29	301200	302437	Unknown	FAD-binding		F	0	0	0	0.8
c8	111832	-	29	304276	305291	Unknown	Unknown		P	0	0	0	0.8
c8	111833	+	29	306467	306944	Unknown	Unknown		T	0	0	0	0.7
c8	82041	+	29	308932	310245	Transmembrane	Unknown		P	0	0	0	0.4
c8	5787	+	29	311440	313322	Transporter	Amino acid permease		F	0	0	0	0.8
c8	111837	+	29	313958	315279	Metabolism	Esterase	Esterase/lipase/thioesterase	F	0	0	0	0.4
c8	111838	+	29	315964	318417	Protein degradation	Peptidase	Alkaline serine protease	F	0	0	0	0.5
c8	69563	-	29	318617	320191	Transporter	MFS	Major facilitator superfamily	F	0	0	0	0.7
c8	69529	+	29	321063	322388	Metabolism	Taurine oxidoreductase		F	0	0	0	0.8
c8	5789	-	29	322659	324275	Metabolism	2ndary	Trichothecene C-15 hydroxylase, Fusarium austroamericanum , cytochrome P450	F	0	0	0	0.7
c8	69555	-	29	327423	328260	Protein degradation	Peptidase A4	Aspergillus niger pepB	F	0	1	1	0.7
c8	123989	+	29	332378	334402	Glycoside hydrolase	Family 7	CBH1, cellobiohydrolase 1	F	0	0	0	0.6
c8	69483	-	29	334496	335510	Transmembrane	Unknown		P	0	0	0	0.8
c8	v1.2_39661	+	29	336401	341963	Unknown	Unknown			0	0	0	0.8
c9	71077	-	50	3755	5906	Regulatory functions	Transcription factor	C2H2-type zinc fingers	P	0	0	0	0.8
c9	112633	+	50	9051	10105	Cell wall?	GST	Glutathione S-transferase homologues to S. cerevisiae ECM4	F	0	0	0	0.8
c9	71072	-	50	10241	14096	Metabolism	2ndary	Polyketide synthase	F	0	0	0	0.8
c9	71059	+	50	14662	16325	Transporter	MFS	Major facilitator superfamily	F	0	0	0	0.9
c9	71080	+	50	16968	19252	Regulatory functions	Transcription factor	Fungal transcriptional regulatory protein, N-terminal	F	0	0	0	0.6
c9	71078	-	50	19791	20955	Regulatory functions	Signalling	Kinase	F	0	0	0	0.9
c9	112638	+	50	22507	23793	Protein degradation	Peptidase S8		P	0	0	0	0.8
ce1	102487	-	1	464672	466353	Metabolism	2ndary	Cytochrome P450	F	0	0	0	0.8
ce1	54723	-	1	467944	468854	Secreted	Peptidoglycan binding domain		P	0	0	0	0.6
ce1	102489	+	1	470470	472068	Secreted	Unknown		T	0	0	0	0.5
ce1	53949	+	1	472797	475920	Glycoside hydrolase	Family 18		A	0	0	0	0.7
ce1	v1.2_9009	-	1	477919	478092	Unknown	Ankyrin			0	0	0	0.8
ce1	53776	-	1	478516	479863	Regulatory functions	Protein kinase		F	0	0	0	0.5
ce1	102492	-	1	479968	481737	Metabolism	SDR	Short-chain dehydrogenase	F	0	0	0	0.4
ce1	73618	-	1	482161	490278	Metabolism	2ndary	PKS	F	0	0	0	0.8

ce1	73621	+	1	491045	499128	Metabolism	2ndary	PKS	F	0	0	0	0.8
ce1	73623	+	1	499862	501401	Metabolism	2ndary	salicylate 1-monooxygenase	F	0	0	0	0.7
ce1	43701	-	1	501681	503623	Transporter	MFS		F	0	0	0	0.8
ce1	102497	+	1	504139	506467	Regulatory functions	Transcription factor	Fungal transcriptional regulatory protein	A	0	0	0	0.7
ce1	73631	-	1	506653	508537	Metabolism	Oxidases/reductases	FAD linked oxidase	F	0	0	0	0.9
ce1	102499	-	1	510531	512829	Regulatory functions	Transcription factor	Fungal transcriptional regulatory protein	A	0	0	0	0.4
ce1	102500	-	1	518168	518762	Secreted	Unknown		T	0	0	0	0.4
ce1	73632	-	1	520228	521337	Carbohydrate esterase	Family 5	axe1, acetylxylanesterase	F	0	0	1	0.6
ce1	73638	-	1	523039	524217	Carbohydrate binding	CBM domain protein	cip1	P	0	0	0	0.2
ce1	73643	-	1	524767	526118	Glycoside hydrolase	Family 61	egl4	F	0	0	0	0.2
ce1	102504	+	1	529180	530027	Unknown	Unknown		T	0	0	0	0.9
ce1	54230	+	1	531948	533538	Unknown	Unknown		F	0	0	0	0.3

Table 7 - Negativel correlating chromosomal gene clusters detected by chromosomal triplets of genes with SPPR under -0.7

See Supplementary Table 6 for legend.

Cluster	Gene ID	Strand	Scaffold	Start	End	Class	Extension	Description	Taxon	D03/D06	D03/HD	D06/HD	Cor2SPPR
cn1	73832	-	1	1121551	1128991	Regulatory functions	Transcription cofactor	S. cerevisiae sip3	F	0	0	0	-0.5
cn1	v1.2_8998	-	1	1121692	1122003	Unknown	Unknown			0	0	0	-0.2
cn1	73837	-	1	1130038	1131795	Protein synthesis	Termination	Eukaryotic peptide chain release factor subunit 1 (eRF1)	F	0	-1	0	-0.8
cn1	102668	+	1	1132400	1132977	Unknown	Unknown		A	0	0	0	-0.8
cn1	43919	-	1	1133056	1135525	Unknown	Unknown		P	0	-1	0	-0.8
cn1	73840	+	1	1137079	1138175	Ribosomal		S. cerevisiae RPL2A	F	0	-1	0	-0.7
cn1	73842	+	1	1139597	1141330	Metabolism	Adenosine kinase		F	0	0	0	-0.2
cn2	120676	+	4	700959	703878	Metabolism	Trehalose	S. cerevisiae NTH1	F	0	-1	0	-0.9
cn2	21758	+	4	705566	707521	Metabolism	TCA	S. cerevisiae ICL1, isocitrate lyase	F	0	-1	-1	-0.9
cn2	45998	-	4	707614	710727	Regulatory functions	Kinase	S. cerevisiae SNF1, serine threonine protein kinase	F	0	-1	0	-0.9
cn3	76540	-	5	1335545	1340010	Regulatory functions	Signalling	S. pombe SPAC11E3.02c C2 domain protein	F	0	0	0	-0.3
cn3	76543	+	5	1342105	1345742	Protein transport	Golgi	S. pombe cnt5 SPBC17G9.08	F	0	-1	0	-0.7
cn3	121061	+	5	1347871	1350841	Cell cycle			F	0	0	0	-0.8
cn3	46702	+	5	1351312	1353178	Protein synthesis	Initiation		F	0	-1	0	-0.8
cn3	76551	+	5	1353954	1355002	Metabolism	Nucleotide		F	0	0	0	-0.8
cn4	121579	+	8	317622	318845	Cell wall		hypothetical peptidoglycan binding	P	0	-1	-1	-0.9
cn4	77481	-	8	318938	321971	Metabolism	Carbon	D-xylulose 5-P/D-fructose 6-P phosphoketolase	F	0	-1	-1	-0.9
cn4	60773	+	8	324580	325242	Unknown	Ankyrin		F	0	-1	0	-0.9
cn4	77485	+	8	325776	328003	Cytoskeleton	Actin tubulin assembly	S. cerevisiae CCT7	F	0	-1	-1	-0.9
cn4	61081	-	8	329639	330701	Regulatory functions	Histone modification	S. cerevisiae SPT10, activator of histone genes, GCN5-related N-acetyltransferase	F	0	0	0	-0.6
cn4	121582	-	8	332757	334414	Ribosomal			F	0	0	0	-0.8
cn4	60796	-	8	334942	336921	Cell cycle?		S. pombe mug70 (Meiotically upregulated gene 70 protein) SPAC24C9.05c	F	0	0	0	-0.7
cn4	121584	+	8	340752	342574	Cell cycle		S. cerevisiae & S. pombe ace2	F	0	0	0	-0.7
cn4	22191	+	8	347355	349441	Cytoskeleton	Actin tubulin assembly	S. cerevisiae CCT6	F	0	-1	0	-0.9
cn4	60780	+	8	349727	351088	Unknown	Unknown		F	0	0	0	-0.7

cn4	77495	-	8	351309	352609	Protein synthesis	Initiation	Eukaryotic translation initiation factor 3 subunit 11	F	0	0	0	-0.6
cn5	47897	-	8	1286592	1291537	Transporter	ABC		F	0	-1	-1	-0.8
cn5	61208	-	8	1296687	1298331	Mitochondrial	Translocation		F	0	0	0	-0.7
cn5	77732	+	8	1299061	1300190	Protein transport	ERAD	S. cerevisiae UBC6	F	0	0	0	-0.7
cn6	22481	-	11	226327	228378	Mitochondrial	Lactate	S. cerevisiae CYB2 cytochrome B2	F	0	0	0	-0.8
cn6	122212	-	11	229623	232882	Protein transport	Vacuolar targeting		F	0	-1	-1	-0.8
cn6	78611	-	11	234996	237713	Regulatory functions	Signalling	Emericella nidulans phospholipase D	F	0	0	0	-0.7
cn6	22484	+	11	239378	240308	Incompatibility	Self incompatibility		F	0	0	0	-0.8
cn7	v1.2_18708	-	13	111788	112551	Unknown	CCHC	CCHC Zn finger protein		0	0	0	-0.7
cn7	64044	-	13	115777	116288	Unknown	Unknown		P	0	0	0	-0.8
cn7	64000	-	13	119227	120461	RNA	Splicing	S.pombe SPBC1289.11 splicing factor Spf38 nuclear mRNA splicing	F	0	0	0	-0.8
cn7	64332	+	13	120896	121646	Protein transport	ER to Golgi	S. cerevisiae BET3	F	0	0	0	-0.8
cn7	108697	+	13	124392	127081	Regulatory functions	Signalling	Neosartorya fischeri NFIA_114980 CAMP-dependent protein kinase pathway protein (Som1)	P	0	0	0	-0.7

Table 8 - Comparison of negatively correlating gene cluster 7 (cn7) between fungal species (Figure 5) as table

Identifier, scaffold (including the species abbreviation as in Figure 5), start and end co-ordinates and strand of genes. Interpro domains (excluding the leading IPR0) and a protein cluster identifier. For genes of the actual cluster 7 an abbreviation as used in Figure 5. As this comparison of 33 fungal species was done using *T. reesei* genome version 1.2 the chromosomal position data is as 1.2 version. The v1.2 genes map to genome version 2.0 as follows: 4494 = v1.2_29323, 63966 = v1.2_34762, 64000 = v1.2_29327, 64044 = v1.2_34759, 64314 = v1.2_34763, 64332 = v1.2_29326, 64370 = v1.2_.34761, 79222 = v1.2_.18490, 108686 = v1.2_34765, 108697 = v1.2_18491, 122506 = v1.2_44380, 122511 = v1.2_42305. Genes v1.2_18540, v1.2_18691, v1.2_18708, v1.2_18546 have no corresponding genes in version 2.0.

Gene	Scaffold	Start	End	Strand	All IPRs	Protein cluster	Abbr.
Afu5g03620	Afun_CM000175	580301	581845	+	04875, 06600	3501	
Afu7g02170	Afun_CM000175	582476	583177	+	00504, 02130	2666	
Afu7g02180	Afun_CM000175	584569	586104	+	02618	1667	
Afu7g02190	Afun_CM000175	588286	588417	+	01878	697	CZ
Afu7g02200	Afun_CM000175	590189	590653	+	01553, 03593, 10995	470	
Afu7g02210	Afun_CM000175	591449	592393	+	00010, 12677	4717	
Afu7g02220	Afun_CM000175	593455	594219	+		7229	
Afu7g02230	Afun_CM000175	599850	600848	+	00504	687	
Afu7g02240	Afun_CM000175	603044	604357	+		8281	
Afu7g02250	Afun_CM000175	605605	605943	+		23966	
Afu7g02260	Afun_CM000175	609840	608638	-	06594	3952	OefA
Afu7g02270	Afun_CM000175	614596	614045	-	07194	1738	bet3
Afu7g02280	Afun_CM000175	615351	616349	+	01680	12	WD
Afu7g02290	Afun_CM000175	621105	623321	+	01382	1159	
Afu7g02300	Afun_CM000175	627173	627922	+		9710	
Afu7g02310	Afun_CM000175	629238	628741	-	00836, 02375, 05764	1409	
AN9081.2	Anid_AACD01000169	79576	78278	-	00873	76	
AN9082.2	Anid_AACD01000169	81549	80287	-		4336	
AN9083.2	Anid_AACD01000169	84210	84710	+	00836, 02375, 05764	1409	
AN9084.2	Anid_AACD01000169	88523	86412	-	01382	1159	
AN9085.2	Anid_AACD01000169	93116	92115	-	01680	12	WD
AN9086.2	Anid_AACD01000169	93928	94416	+	07194	1738	bet3
AN9087.2	Anid_AACD01000169	97699	99258	+	06594	3952	OefA

AN9088.2	Anid_AACD01000169	101830	101549	-		20628	
AN9089.2	Anid_AACD01000169	104758	103346	-		8281	
AN9090.2	Anid_AACD01000169	107738	106869	-	00504	687	
AN9091.2	Anid_AACD01000169	113423	112452	-		7229	
AN9092.2	Anid_AACD01000169	115305	114367	-	01553, 03593, 10995	470	
AN9093.2	Anid_AACD01000169	118156	118043	-	01878	697	CZ
AN9094.2	Anid_AACD01000169	120744	120010	-	02618	1667	
AN9095.2	Anid_AACD01000169	123576	122851	-	00504, 02130	2666	
AN9096.2	Anid_AACD01000169	127501	128625	+		20629	
CHG08397.1	Cglo_supercontig1.6	192608	193426	+	00757, 08985	2595	
CHG08398.1	Cglo_supercontig1.6	194582	196939	+	02110	31	
CHG08399.1	Cglo_supercontig1.6	197901	198095	+		41683	
CHG08401.1	Cglo_supercontig1.6	203079	202162	-	00172, 07867, 12132	34	
CHG08402.1	Cglo_supercontig1.6	206412	205777	-	06594	3952	OefA
CHG08403.1	Cglo_supercontig1.6	208486	208623	+		41684	
CHG08403.1	Cglo_supercontig1.6	208791	208922	+		41684	
CHG08404.1	Cglo_supercontig1.6	211789	210743	-		41685	
CHG08405.1	Cglo_supercontig1.6	213263	212946	-	07194	1738	bet3
CHG08406.1	Cglo_supercontig1.6	214517	215314	+	01680	12	WD
CHG08407.1	Cglo_supercontig1.6	218695	219378	+		6460	UN1
CHG08408.1	Cglo_supercontig1.6	222536	222931	+	01878	697	CZ
CHG08409.1	Cglo_supercontig1.6	225949	226809	+	08313, 08928	555	
CHG08410.1	Cglo_supercontig1.6	228462	227431	-	06353, 06357	277	
CHG08411.1	Cglo_supercontig1.6	232267	231335	-		15988	
CHG08413.1	Cglo_supercontig1.6	234789	234319	-		41686	
CIMG.07748.2	Cimm_supercontig_2.4	1124567	1124812	+		44869	
CIMG.07749.2	Cimm_supercontig_2.4	1126342	1125317	-		44870	
CIMG.07750.2	Cimm_supercontig_2.4	1129139	1128243	-	01680	12	WD
CIMG.07751.2	Cimm_supercontig_2.4	1130523	1130906	+	07194	1738	bet3
CIMG.07752.2	Cimm_supercontig_2.4	1134096	1135967	+	06594	3952	OefA
CIMG.07753.2	Cimm_supercontig_2.4	1138588	1137557	-		8281	
CIMG.07754.2	Cimm_supercontig_2.4	1141210	1140929	-		44871	
CIMG.07755.2	Cimm_supercontig_2.4	1142604	1141471	-		44872	
CIMG.07756.2	Cimm_supercontig_2.4	1145813	1145067	-	00504	687	
CIMG.07757.2	Cimm_supercontig_2.4	1148535	1148834	+		44873	
CIMG.07758.2	Cimm_supercontig_2.4	1152612	1151770	-		7229	
CIMG.07759.2	Cimm_supercontig_2.4	1154269	1153391	-	01553, 03593, 10995	470	
CIMG.07760.2	Cimm_supercontig_2.4	1156147	1155737	-	01878	697	CZ
CIMG.07761.2	Cimm_supercontig_2.4	1158230	1157349	-	02618	1667	
CIMG.07762.2	Cimm_supercontig_2.4	1160978	1160025	-	00504, 02130	2666	
CIMG.07764.2	Cimm_supercontig_2.4	1164461	1165582	+		44875	
FG02639.1	Fgra_AACM01000132	96389	96949	+	03511	2005	
FG02640.1	Fgra_AACM01000132	99617	97341	-	00449, 01394, 01607	1726	
FG02641.1	Fgra_AACM01000132	101928	100993	-	03663, 05828, 05829, 07114	1	
FG02642.1	Fgra_AACM01000132	105470	106615	+		51068	
FG02643.1	Fgra_AACM01000132	109435	108221	-		4674	
FG02644.1	Fgra_AACM01000132	114290	112320	-		51069	
FG02645.1	Fgra_AACM01000132	116960	117670	+	01300	992	
FG02646.1	Fgra_AACM01000132	120458	120015	-	01878	697	CZ
FG02647.1	Fgra_AACM01000132	124673	123942	-		6460	UN1
FG02648.1	Fgra_AACM01000132	127425	126493	-	01680	12	WD
FG02649.1	Fgra_AACM01000132	128475	128786	+	07194	1738	bet3
FG02650.1	Fgra_AACM01000132	132182	133369	+	06594	3952	OefA
FG02651.1	Fgra_AACM01000132	136503	135601	-	00757, 08985	2595	
FG02652.1	Fgra_AACM01000132	139059	138559	-		6527	
FG02653.1	Fgra_AACM01000132	139968	140219	+		8441	
FG02654.1	Fgra_AACM01000132	141418	143082	+		16637	
NCU05786.1	Ncra_AABX01000353	48733	48840	+		58377	
NCU05787.1	Ncra_AABX01000353	52348	51704	-		58378	
NCU05789.1	Ncra_AABX01000353	58303	59001	+	00757, 08985	2595	
NCU05790.1	Ncra_AABX01000353	62006	60426	-	01294, 01789, 03018, 03594, 03661, 04358, 05467, 11006	2790	
NCU05791.1	Ncra_AABX01000353	68905	67919	-	06594	3952	OefA
NCU05792.1	Ncra_AABX01000353	70760	71017	+		58379	
NCU05793.1	Ncra_AABX01000353	72980	73159	+		58380	
NCU05794.1	Ncra_AABX01000353	77861	77484	-		58381	
NCU05795.1	Ncra_AABX01000353	79581	79126	-		58382	
NCU05796.1	Ncra_AABX01000353	81176	80862	-	07194	1738	bet3
NCU05797.1	Ncra_AABX01000353	82623	83483	+	01680	12	WD
NCU05798.1	Ncra_AABX01000353	86370	87374	+		6460	UN1
NCU05799.1	Ncra_AABX01000353	91528	91301	-		58383	
NCU05800.1	Ncra_AABX01000353	92406	93020	+	01878	697	CZ
NCU05801.1	Ncra_AABX01000353	98441	96342	-		17306	
NCU05802.1	Ncra_AABX01000353	105958	101744	-	01410, 01650, 02464, 07502, 11545, 11709	37	
v1.2_34765	TrRee_scaffold_13	88237	86693	-	01461, 09007	4751	
v1.2_44380	TrRee_scaffold_13	93117	93551	+	02952	90349	
v1.2_18540	TrRee_scaffold_13	94322	94504	+	00073, 00379, 03089	89159	
v1.2_18691	TrRee_scaffold_13	97014	96508	-	07219	1759	
v1.2_34763	TrRee_scaffold_13	98738	99220	+	11701	248	
v1.2_34762	TrRee_scaffold_13	103819	104148	+	03663, 05828, 05829, 07114	1	
v1.2_34761	TrRee_scaffold_13	107965	106226	-	01300	992	
v1.2_18708	TrRee_scaffold_13	112321	111788	-	01878	697	CZ
v1.2_34759	TrRee_scaffold_13	116830	115982	-		6460	UN1
v1.2_29327	TrRee_scaffold_13	120092	119334	-	01680	12	WD
v1.2_29326	TrRee_scaffold_13	120896	121642	+	07194	1738	bet3
v1.2_18491	TrRee_scaffold_13	125200	126183	+	06594	3952	OefA
v1.2_42305	TrRee_scaffold_13	129368	128586	-	00757, 08985	2595	
v1.2_29323	TrRee_scaffold_13	132617	132087	-		6527	
v1.2_18546	TrRee_scaffold_13	133319	133113	-		89160	
v1.2_18490	TrRee_scaffold_13	135784	136452	+		12274	

Table 9 - Candidate novel genes detected with sparse arrays

Identifier of the novel gene, scaffold and co-ordinates, differential expression significance test results for the three different contrast (see Supplemenatry Table 2) and correlation to specific protein production rate. Remarks details neighbouring genes of note, especially for novel genes with significant differential expression or correlation.

Name	Scaffold	Start	End	D03/D06	D03/HD	D06/HD	Cor2SPPR	Remarks
1_1624	1	1422866	1423277	0	0	0	0.52	
1_1764	1	1354842	1355171	0	0	0	0.64	
1_1789	1	1342889	1343416	0	-1	0	-0.64	Between 43974 hut1 glycosylation factor and 73895 with similarity to telomer related proteins
1_3448	1	567068	567676	0	1	0	0.68	Between 73654 bZIP transcription factor and 54893 signalling kinase mob2
1_3454	1	563951	564329	0	0	0	-0.09	
1_3463	1	560148	560477	0	0	0	-0.01	
1_3944	1	340742	341104	0	0	0	-0.22	
1_72	1	2165189	2165534	0	1	0	0.73	Between unknown and v1.2 gene
1_94	1	2150066	2150455	0	0	0	0.62	
10_1091	9	528125	528986	0	1	0	0.78	Between unknown and 61819 doc1 anaphase promoting
10_1754	9	835264	835581	0	0	0	0.28	
10_1832	9	876975	877640	0	0	0	0.63	
10_1841	9	880777	881140	0	0	0	0.39	
10_196	9	88874	89228	0	0	0	0.56	
10_591	9	279191	279797	0	0	0	0.38	
11_1383	3	296230	296661	0	0	0	-0.23	
11_1444	3	268473	269204	0	0	0	0.76	
11_1798	3	115291	115630	0	0	0	0.23	Next to ire1
11_782	3	569507	569999	0	0	0	0.61	
11_87	3	898365	898733	0	0	0	0.02	
12_1290	15	288959	289244	0	0	0	0.09	
12_1323	15	275355	275738	0	0	0	-0.23	
13_1237	14	585575	585889	0	0	0	0.16	
13_1422	45	63972	64706	0	0	0	0.82	Between 70934 oligopeptide transporter and scaffold end
13_1613	14	763798	764130	0	1	0	0.71	Between unknowns
13_1642	14	778720	779689	0	0	0	0.59	
14_1144	12	487635	488241	0	0	0	0.32	
14_1295	12	416882	417143	0	0	0	0.61	
14_172	12	926824	927403	0	0	0	0.66	
14_1750	12	191713	192348	0	0	0	0.62	
14_1755	12	189063	189692	0	0	0	-0.20	
14_334	12	862648	863289	0	0	0	0.56	
15_1138	17	257883	258149	0	0	0	-0.01	
15_517	17	562390	562699	0	0	0	0.40	
16_41	1	3734232	3734556	0	0	0	0.60	
16_470	1	3541260	3541670	0	0	0	0.51	
16_765	1	3400309	3400734	0	0	0	-0.59	
16_840	1	3361562	3361849	0	0	0	0.25	
17_578	18	402235	402751	0	0	0	-0.27	
17_766	18	309983	310363	0	0	0	-0.07	
18_1160	16	268827	269171	0	0	0	0.54	
18_1364	16	178483	179220	0	0	0	0.74	
19_1159	19	531096	531483	0	0	0	-0.08	
19_431	19	191393	192428	0	0	0	0.71	
19_455	19	203731	204390	0	0	0	0.60	
19_868	19	389452	389748	0	1	0	0.69	Between unknown and 80593 pap2 RNA quality control
2_1044	2	645934	647841	0	0	0	-0.78	
2_1351	2	789518	789857	0	0	0	-0.46	Next to cre1
2_1404	2	814943	815342	0	0	0	0.60	
2_238	2	270648	271208	0	0	0	0.26	
2_2586	2	1388525	1389181	0	0	0	-0.31	
2_2919	2	1548526	1548883	0	0	0	0.24	
2_3064	2	1631486	1632355	0	0	0	0.52	
2_857	2	548236	548724	0	1	1	0.78	Between unknown transporters
20_255	20	497845	498544	0	0	0	0.04	
20_864	20	210277	210682	0	0	0	0.37	
21_266	11	672193	672625	0	0	0	0.70	
21_435	11	752591	752972	0	0	0	0.11	
23_469	21	327277	327606	0	0	0	-0.29	
24_301	11	407065	407484	0	0	0	-0.55	
24_795	11	155005	155326	0	0	0	-0.44	
24_868	11	122450	123118	0	0	0	0.38	

25_1011	22	33336	33750	0	1	1	0.77	Between 81007 rio1 cell cycle kinase and 123445 IPR001138 trancription factor
25_407	22	346020	346377	0	0	0	0.08	
25_415	22	340418	340852	0	0	0	0.48	
26_227	3	1671469	1671750	0	0	0	-0.18	
26_502	3	1543443	1544150	0	0	0	-0.32	
26_552	3	1520215	1520662	0	0	0	0.52	
26_610	3	1486786	1487658	0	0	0	0.01	
27_145	23	63661	64014	0	1	1	0.79	Between 67941 putative transmembrane and 81149 F-box protein
27_531	23	245738	246028	0	0	0	-0.51	
27_637	23	290411	290779	0	0	0	0.43	
27_731	23	340060	340470	0	-1	-1	-0.69	Between 110960 Ras2 like GTPase and 123556 with similarity to opy2 high-osmolarity glycerol (HOG) signalling
27_734	23	340615	340962	0	0	0	-0.28	
28_34	24	485172	485547	0	1	0	0.69	Between 23190 clp1 mRNA related and 81430 P-type ATPase pump
28_371	24	329677	329983	0	0	0	0.05	
29_534	25	188564	188872	0	0	1	0.22	Between unknowns
3_1276	6	837620	838066	0	0	0	0.22	
3_2105	6	475784	476134	0	0	0	0.69	
3_2138	6	459111	459518	0	0	0	-0.61	
31_252	29	124732	125202	0	0	0	0.36	
33_421	8	1271898	1272243	0	0	0	-0.47	
33_606	8	1358151	1358504	0	0	0	-0.34	
38_15	4	236850	237164	0	0	0	0.65	
38_452	4	18115	18441	0	0	0	0.43	
39_56	32	198103	198396	0	0	0	0.67	
4_1511	5	871623	871994	0	0	0	0.42	
4_1530	5	863415	864458	0	0	0	0.41	
4_1579	5	836102	836611	0	0	0	-0.44	
4_2310	5	469257	470004	0	0	0	-0.39	
4_2481	5	384735	385082	0	1	1	0.69	Between unknown and 46421 putative mannosyltransferase involved in protein glycosylation
4_2591	5	330157	330439	0	0	0	0.62	
4_2609	5	321636	322046	0	0	0	0.51	
4_319	5	1432904	1433503	0	0	0	0.94	Between 58928 chd1 chromatin remodelling factor and v1.2_20888 translation initiation factor
4_475	5	1359152	1360300	0	0	0	0.38	
4_813	5	1204591	1205058	0	1	0	0.73	Between 76505 seb1 C2H2-type zinc finger stress protein and unknown
42_300	33	154967	155410	0	0	0	0.05	
43_73	9	1181925	1182387	0	0	0	0.49	
44_120	34	107553	107945	0	0	0	0.82	Between 124113 GPCR and 82296 membrane transporter
44_243	34	39115	39504	0	0	0	0.24	
45_18	12	151545	151908	0	0	0	0.14	
46_264	16	131309	131710	0	-1	0	-0.67	is 122864
47_238	5	1691841	1692152	0	0	0	-0.65	
5_1266	7	751732	752052	0	0	0	0.15	
5_1784	7	515205	515843	0	0	0	0.11	
5_1856	7	478574	478924	0	0	0	0.72	
5_217	7	1238618	1239016	0	0	0	0.62	
5_2195	7	327787	329404	0	0	0	0.51	
5_2510	7	180823	181177	0	0	0	-0.77	
50_81	36	96949	97273	0	0	0	0.60	
6_1171	13	336448	336891	0	0	0	0.64	
6_2389	27	252264	252558	0	-1	0	-0.65	Surrounded by unknown genes
6_766	13	523205	524600	0	0	0	-0.32	
7_1555	10	420427	420769	0	0	0	0.75	
7_1746	10	335489	335840	0	0	0	0.55	
8_1028	4	1206242	1206716	0	0	0	-0.33	
8_1718	4	894365	894938	0	0	0	0.60	
8_1998	4	757648	758247	0	0	0	0.47	
8_317	4	1553132	1553452	0	0	0	-0.39	
8_32	4	1677154	1677750	0	0	0	0.75	
8_735	55	20486	22082	0	0	0	0.49	
9_1935	8	919787	920273	0	1	0	0.72	Between 121664 glutamate decarboxylase and 107035 GTPase
9_273	8	129617	130190	0	0	0	0.64	
9_407	8	185130	185703	0	0	0	0.04	

9_916	8	421250	422230	0	0	0	0.51	

Table 10 - Correlation between transcriptomics and proteomics data (main paper Figure 6) as table

For each gene its identifier, authors' manual annotation (Class and Extension) and name used in the main paper Figure 6 ('Remarks'). log2 fold change of proteomics (_P), transcriptomics (_T) and number of protein spots on 2D-gel (_S) in the two contrasts studied (D03/D06 and D03/HD). For proteins with multiple spots the log2 fold change is the most negative or positive depending on which sign the median log2 fold change of all the spots had. In addition for both contrasts protein - transcript relationship classifications based on translational control efficiency ratio (TCEr, _C) and on main paper Figure 5 (_I). For cases where both the protein and transcript fold change was above 1.5: Q1 = both protein and transcript have positive fold change above, Q2 protein +, transcripts -, Q3 protein - , transcript - and Q4 protein -, transcript + (main paper Figure 5). For cases where transcript fold change was below 0.75 'T0' for positive fold change of the protein and 'T-0' for negative fold change respectively.

Gene ID	Class	Extension	Remarks	D3/D6_P	D3/D6_T	D3/D6_C	D3/D6_S	D3/D6_I	D3/HD_P	D3/HD_T	D3/HD_C	D3/HD_S	D3/HD_I
22004	Metabolism	Carbon	gar1	2.3	1.0	2.34	1	Q1	2.3	0.5	3.59	1	
79921	Protein secretion	Glycosylation, GH Family 92	79921	1.0	0.7	1.23	1	Q1					
119731	Mitochondrial	Heat shock protein	119731	-1.7	-0.8	0.51	2	Q3	0.9	-0.8	3.24	1	Q2
121620	Metabolism	Thiamine	121620	-1.2	-1.4	1.16	1	Q3	-1.5	-1.3	0.85	1	Q3
108459	Metabolism	Amino acid	108459	-1.1	-0.7	0.76	1	Q3					
54694	Metabolism	Oxidoreductase	54694	-1.9	-1.0	0.56	1	Q3					
120053	Mitochondrial	Folding	hsp70	-1.4	-0.9	0.71	2	Q3					
4308	Protein degradation	Peptidase	4308	-2.1	-0.9	0.42	2	Q3					
109234	Protein degradation	Peptidase, ER	109234	-1.3	-1.2	0.96	1	Q3					
71363	Protein synthesis	Elongation	71363	-1.3	-0.7	0.65	1	Q3					
82049	Regulatory functions	Protein kinase	82049	-2.0	-1.1	0.55	2	Q3					
71326	Carbohydrate esterase	Family 9		2.4	-0.1	5.64	1	T0	1.9	-0.0	3.86	1	T0
72567	Glycoside hydrolase	Family 6	cbh2	2.3	0.1	4.68	1	T0	1.0	0.1	1.84	1	T0
23346	Glycoside hydrolase	Family 20	exc2y	1.8	0.2	2.90	1	T0					
58282	Metabolism			1.5	-0.3	3.42	2	T0					
52267	Metabolism	Carbon	mpdA	2.9	0.3	6.47	1	T0					
56920	Protein degradation	Peptidase		1.0	0.3	1.70	1	T0	-0.7	-0.4	0.85	1	
66608	Protein degradation	Peptidase		1.6	0.1	2.86	1	T0					
105692	Protein secretion	Golgi/Protein transport	105692	1.8	0.0	3.52	1	T0					
119890	Protein secretion	Protein folding	119890	1.0	0.0	2.01	1	T0					
74118	Protein secretion	Targeting to ER membrane	74118	0.6	-0.1	1.60	1	T0	0.9	-0.5	2.68	1	
47838	Regulatory functions	Nucleasome assembly	ASF1	1.0	0.1	1.94	1	T0					
21737	Unknown			1.2	0.1	2.15	1	T0					
120534	Cytoskeleton	Actin	sac6	-1.1	-0.2	0.54	1	T-0	-0.6	-0.8	1.12	1	Q3
2687	Mitochondrial	Chaperone		-2.0	-0.1	0.28	1	T-0	-1.8	-0.1	0.32	1	T-0
123714	Stress	Oxidative		-1.2	-0.2	0.52	1	T-0	-0.9	-0.0	0.55	1	T-0
79993	Cytoskeleton	Actin/tubulin assembly		-1.2	-0.3	0.55	1	T-0					
81576	Metabolism	Amino acid		-1.0	0.2	0.42	1	T-0					
80231	Metabolism	Carbon	glk1	-1.2	-0.1	0.45	1	T-0					
21836	Metabolism	Glycolysis	PGM1-2	-1.3	-0.2	0.49	1	T-0	-0.6	-0.4	0.84	1	
80872	Metabolism	Nucleotide	ADE5,7	-1.0	-0.3	0.59	1	T-0					
123149	Metabolism	Oxidoreductase		-1.9	-0.0	0.27	1	T-0					
121166	Metabolism	Sterol		-1.1	-0.2	0.56	1	T-0					
57947	Mitochondrial	Heme biosynthesis		-1.0	-0.3	0.58	1	T-0					
50249	Protein degradation	Peptidase		-1.4	-0.3	0.47	1	T-0	-1.5	-0.5	0.51	1	
81972	Protein secretion	Golgi to membrane		-1.6	-0.2	0.38	1	T-0					
81822	Protein synthesis	tRNA synthesis		-1.3	-0.2	0.46	1	T-0					
48146	Protein synthesis	tRNA-synthesis		-1.3	-0.2	0.47	1	T-0					
69742	Unknown	Unknown		-1.1	-0.1	0.51	1	T-0					
33894	Unknown	Unknown		-1.0	0.1	0.46	1	T-0					
74198	Protein secretion	Glycosylation, GH Family 92	74198	1.6	0.5	2.20	1		2.7	1.1	2.99	2	Q1
123668	Regulatory functions	Chromatin remodeling	123668	0.7	0.4	1.21	1		0.9	0.9	1.00	1	Q1

79686	RNA	PolyA						1.6	-0.8	5.42	3	Q2
v1.2_46348	Unknown	TPR repeat	v1.2_46348					0.6	-0.6	2.44	1	Q2
22197	Glycoside hydrolase	Family 1	cel1b					-0.6	-0.9	1.21	1	Q3
74278	Metabolism	2ndary						-2.0	-1.0	0.51	1	Q3
123471	Metabolism	Amino acid	LYS21	-1.2	-0.5	0.59	2	-1.0	-0.8	0.87	1	Q3
68036	Metabolism	Amino acid	MET3					-2.1	-1.8	0.82	1	Q3
77481	Metabolism	Carbon	77481					-1.8	-2.4	1.53	1	Q3
123026	Metabolism	PPP	TAL1					-1.1	-1.1	1.02	1	Q3
121534	Metabolism	Pyruvate	PDC1-5-6					-1.9	-1.8	0.96	2	Q3
51365	Protein degradation	Peptidase						-1.3	-0.7	0.65	1	Q3
82534	Protein folding	Cytoplasmic						-2.2	-1.2	0.49	1	Q3
120235	Protein synthesis	Elongation						-3.3	-0.8	0.19	2	Q3
4537	Protein synthesis	tRNA-synthesis		-1.1	-0.3	0.59	1	-1.0	-1.0	0.95	1	Q3
77747	RNA	Binding						-2.0	-1.0	0.53	1	Q3
73818	Stress	Oxidative						-1.3	-1.1	0.86	1	Q3
55362	Stress	Protein folding, HSP70						-1.8	-0.8	0.52	1	Q3
50542	Transporter	ABC		-1.4	-0.3	0.47	1	-1.2	-0.6	0.68	1	Q3
1751	Unknown	Oxidoreductase	1751					-1.1	-1.6	1.47	1	Q3
123989	Glycoside hydrolase	Family 7	cbh1					1.4	0.1	2.47	1	T0
49081	Glycoside hydrolase	Family 74	cel74a	-1.3	0.4	0.29	2	1.5	0.1	2.65	2	T0
102382	Metabolism	2ndary						1.2	-0.3	2.70	1	T0
75294	Metabolism	Amino acid						0.6	-0.1	1.60	1	T0
51103	Metabolism	Amino acid						1.1	-0.2	2.54	1	T0
80003	Metabolism	Amino acid						1.3	0.3	2.06	1	T0
108463	Metabolism	Amino acid						1.4	-0.0	2.79	1	T0
66647	Metabolism	Haloacid dehalogenase						1.2	0.1	2.08	1	T0
68608	Metabolism	Thiamine						1.9	0.0	3.71	3	T0
122920	Protein secretion	bip1	bip1					0.9	-0.3	2.17	2	T0
111131	Protein synthesis	Initiation						1.4	-0.3	3.11	1	T0
71092	Secreted	Phosphatase		1.5	0.5	1.93	1	1.1	-0.1	2.16	1	T0
64561	Transporter	Electron transport chain						0.7	-0.2	1.88	1	T0
122696	Unknown	Pleckstrin						1.7	-0.1	3.47	1	T0
105381	Unknown	Unknown						1.1	0.1	1.99	1	T0
5614	Unknown	Unknown						1.2	-0.0	2.42	1	T0
78968	Unknown	Unknown						2.5	0.0	5.60	1	T0
68304	Protein degradation	Proteasome						-1.1	-0.3	0.56	1	T-0
47020	Protein secretion	Phosphatidylinositol transfer						-1.5	0.0	0.36	1	T-0
107639	Unknown	Unknown						-1.2	0.2	0.37	1	T-0
79568	Cell wall, glycosylation, GPI		QRI1	-2.1	-0.4	0.30	1					
121276	Cytoskeleton	Actin binding						-1.5	-0.5	0.51	1	
73733	Cytoskeleton	Actin/tubulin assembly		-1.2	-0.3	0.54	1					
102581	DNA	Excision repair						0.6	-0.6	2.29	1	
76672	Glycoside hydrolase	Family 3	bgl1					1.5	0.5	1.94	1	
104797	Glycoside hydrolase	Family 3						1.8	0.5	2.43	1	
120312	Glycoside hydrolase	Family 5	egl2					1.4	0.5	1.92	2	
123456	Glycoside hydrolase	Family 65	treA					-1.7	-0.5	0.45	3	
122081	Glycoside hydrolase	Family 7	egl1					1.8	0.3	2.83	1	
81014	Metabolism	2ndary		-1.4	-0.4	0.49	1	-1.0	-0.5	0.72	1	
4117	Metabolism	Amino acid		-1.1	-0.4	0.63	1					
73903	Metabolism	Carbon	SOL1-2-3-4					-1.5	-0.3	0.43	1	
123382	Metabolism	Heme						-2.2	-0.5	0.31	1	
79373	Metabolism	Nucleotide						1.5	-0.5	3.89	1	
56840	Metabolism	Oxidoreductase						1.1	0.5	1.47	1	
23090	Metabolism	Oxidoreductases						1.1	0.3	1.76	1	
121826	Metabolism	TCA	acl2	-1.1	-0.4	0.63	1					
43664	Metabolism	UPD-glucose						-0.4	-1.0	1.45	1	
74449	Mitochondrial	Protein misfolding						-1.1	-0.4	0.59	1	
47829	Mitochondrial	Respiration						-1.4	-0.6	0.57	1	
81450	Mitochondrial	Transport						-1.3	-0.3	0.51	1	
123114	Protein folding	Cytoplasmic		-1.8	-0.4	0.38	2					
121126	Protein secretion		121126	-1.7	-0.3	0.40	1					
122415	Protein secretion	Protein folding	pdi1					1.3	0.6	1.72	2	
121169	Protein secretion	vacuolar sorting/endocytosis	121169					1.2	-0.5	3.25	1	
77495	Protein synthesis	Initiation		-1.2	-0.3	0.53	1	-0.8	-0.3	0.72	1	

5227	Protein synthesis	tRNA-synthesis		-1.3	-0.5	0.58	1					
120120	Regulatory functions	Chromatin remodeling	120120	-1.0	-0.4	0.63	1		-1.1	-0.4	0.60	1
21324	Regulatory functions	Protein phosphatase	21324	-1.2	-0.3	0.54	1					
122230	RNA								0.7	-0.5	2.40	2
121717	Unknown	Unknown		2.3	0.4	3.74	1					

Table 11 - Proteome analysis details by 2D gel spot

For each spot its expression behaviour ('Expression' , i.e. -D03/D06 are proteins more abundant in D06 than in D03), gene identifier, spot identifier, average signal in first (i.e. D03 in -D03/D06) and second (i.e. D06 in -D03/D06) condition of a comparison and log2 fold change of the average signals. Then from Mascot identifications: score, numbers of query peptides that matched to the protein and percentage of protein sequence covered by the peptides.

Expression	Gene ID	Spot ID	Signal 1	Signal 2	Fold change	Score	Queries matched	Sequence coverage
-D03/D06	108459	pH_3_5.6_355	0.96	2.08	1.11	2017	77	35
-D03/D06	109234	pH_5.3_6.5_1824	0.60	1.44	1.26	484	19	27
-D03/D06	119731	pH_3_5.6_891	0.38	1.19	1.64	1843	68	51
-D03/D06	119731	pH_3_5.6_892	0.40	1.32	1.72	2110	171	58
-D03/D06	120053	pH_3_5.6_691	0.68	1.55	1.19	1940	78	44
-D03/D06	120053	pH_3_5.6_715	0.69	1.83	1.40	2156	146	43
-D03/D06	120120	pH_3_5.6_1504	0.69	1.40	1.02	1093	52	67
-D03/D06	120534	pH_3_5.6_743	0.80	1.73	1.11	772	15	28
-D03/D06	121126	pH_5.3_6.5_1082	0.40	1.25	1.66	606	17	33
-D03/D06	121166	pH_5.3_6.5_1557	0.66	1.38	1.06	754	15	22
-D03/D06	121620	pH_5.3_6.5_1621	0.52	1.22	1.23	851	36	54
-D03/D06	121826	pH_3_5.6_949	0.53	1.11	1.08	1505	58	54
-D03/D06	123114	pH_3_5.6_582	0.86	1.79	1.06	2550	251	48
-D03/D06	123114	pH_3_5.6_583	0.59	2.13	1.84	1689	54	41
-D03/D06	123149	pH_5.3_6.5_1978	0.39	1.49	1.93	559	15	54
-D03/D06	123471	pH_5.3_6.5_1303	0.55	1.30	1.23	807	28	45
-D03/D06	123471	pH_5.3_6.5_1315	0.59	1.23	1.06	788	21	47
-D03/D06	123714	pH_5.3_6.5_1946	0.61	1.38	1.18	686	22	57
-D03/D06	21324	pH_3_5.6_756	0.77	1.79	1.21	943	20	30
-D03/D06	21836	pH_3_5.6_911	0.51	1.23	1.26	268	7	11
-D03/D06	2687	pH_5.3_6.5_753	0.36	1.40	1.98	805	17	30
-D03/D06	33894	pH_3_5.6_673	0.90	1.86	1.04	131	4	8
-D03/D06	4117	pH_5.3_6.5_326	0.59	1.23	1.07	1861	47	41
-D03/D06	4308	pH_5.3_6.5_569	0.62	1.33	1.10	1243	31	36
-D03/D06	4308	pH_5.3_6.5_577	0.40	1.71	2.11	655	15	21
-D03/D06	4537	pH_3_5.6_395	0.94	1.99	1.08	2245	57	39
-D03/D06	48146	pH_5.3_6.5_375	0.53	1.31	1.29	738	21	20
-D03/D06	49081	pH_3_5.6_434	0.70	1.76	1.32	661	19	15
-D03/D06	49081	pH_3_5.6_444	0.90	1.61	0.84	584	15	15
-D03/D06	50249	pH_3_5.6_621	0.79	2.00	1.35	134	4	7
-D03/D06	50542	pH_5.3_6.5_869	0.46	1.22	1.42	775	25	34
-D03/D06	5227	pH_5.3_6.5_339	0.62	1.53	1.30	546	13	14
-D03/D06	54694	pH_5.3_6.5_1526	0.39	1.44	1.87	680	27	37
-D03/D06	57947	pH_5.3_6.5_1181	0.60	1.24	1.04	588	14	29
-D03/D06	69742	pH_3_5.6_1480	0.78	1.65	1.07	315	9	45
-D03/D06	71363	pH_5.3_6.5_419	0.66	1.63	1.30	503	11	14
-D03/D06	73733	pH_3_5.6_860	0.68	1.57	1.21	517	7	15
-D03/D06	77495	pH_3_5.6_1503	0.93	2.18	1.23	533	21	39
-D03/D06	79568	pH_3_5.6_932	0.26	1.13	2.13	424	10	22
-D03/D06	79993	pH_3_5.6_835	0.68	1.52	1.16	489	12	16
-D03/D06	80231	pH_3_5.6_879	0.58	1.36	1.24	918	24	39
-D03/D06	80872	pH_3_5.6_605	0.91	1.87	1.04	572	15	16
-D03/D06	81014	pH_5.3_6.5_274	0.62	1.67	1.44	1316	31	30
-D03/D06	81576	pH_3_5.6_374	0.97	2.00	1.04	1004	24	20
-D03/D06	81822	pH_5.3_6.5_488	0.59	1.47	1.32	388	10	15
-D03/D06	81972	pH_3_5.6_919	0.37	1.11	1.57	899	31	40
-D03/D06	82049	pH_3_5.6_1220	0.31	1.23	1.99	791	27	43
D03/D06	105692	pH_3_5.6_1061	0.85	0.24	-1.84	258	12	32
D03/D06	119890	pH_3_5.6_1197	0.94	0.46	-1.03	1142	88	52
D03/D06	121717	pH_5.3_6.5_2048	1.65	0.34	-2.32	428	11	44
D03/D06	123668	pH_3_5.6_1521	1.63	1.03	-0.67	766	40	77
D03/D06	21737	pH_5.3_6.5_2034	1.41	0.63	-1.15	314	7	27
D03/D06	22004	pH_5.3_6.5_1704	1.63	0.34	-2.25	502	13	38
D03/D06	23346	pH_5.3_6.5_803	1.56	0.45	-1.79	300	8	17
D03/D06	47838	pH_3_5.6_1239	1.21	0.59	-1.03	236	9	21
D03/D06	52267	pH_3_5.6_1102	1.32	0.17	-2.94	825	29	49
D03/D06	56920	pH_3_5.6_1257	1.34	0.65	-1.06	591	24	27
D03/D06	58282	pH_3_5.6_1354	1.55	0.68	-1.18	537	17	14
D03/D06	58282	pH_5.3_6.5_1912	1.76	0.63	-1.47	132	4	4
D03/D06	66608	pH_5.3_6.5_639	1.50	0.50	-1.60	295	6	6
D03/D06	71092	pH_5.3_6.5_1179	1.51	0.55	-1.47	721	28	58
D03/D06	71326	pH_5.3_6.5_1691	1.83	0.35	-2.40	593	16	15
D03/D06	72567	pH_3_5.6_1136	1.37	0.28	-2.32	437	16	13
D03/D06	74118	pH_3_5.6_1152	0.97	0.64	-0.60	1287	66	62

D03/D06	74198	pH_5.3_6.5_570	1.34	0.43	-1.64	867	24	25
D03/D06	79921	pH_3_5.6_496	1.63	0.81	-1.00	307	8	9
-D03/HD	107639	pH_5.3_6.5_1879	0.55	1.27	1.20	590	11	45
-D03/HD	120120	pH_3_5.6_1306	0.62	1.32	1.08			
-D03/HD	120235	pH_5.3_6.5_1865	0.18	1.72	3.24	1219	45	22
-D03/HD	120235	pH_5.3_6.5_742	0.24	1.24	2.35	661	16	20
-D03/HD	120534	pH_3_5.6_588	0.74	1.13	0.61	772	15	28
-D03/HD	121276	pH_5.3_6.5_515	0.68	1.91	1.49	1510	31	32
-D03/HD	121534	pH_5.3_6.5_1184	0.41	1.50	1.86	1000	25	43
-D03/HD	121534	pH_5.3_6.5_1191	0.65	1.40	1.10	1078	26	48
-D03/HD	121620	pH_5.3_6.5_1848	0.44	1.28	1.53	851	36	54
-D03/HD	123026	pH_3_5.6_1099	0.63	1.33	1.08	1477	127	61
-D03/HD	123382	pH_5.3_6.5_1858	0.35	1.59	2.19	564	15	38
-D03/HD	123456	pH_5.3_6.5_329	0.81	2.64	1.70	392	7	9
-D03/HD	123456	pH_5.3_6.5_334	0.88	2.33	1.40	462	10	10
-D03/HD	123456	pH_5.3_6.5_347	0.87	2.11	1.28	657	15	15
-D03/HD	123471	pH_5.3_6.5_1303	0.55	1.30	1.23	807	28	45
-D03/HD	123471	pH_5.3_6.5_1315	0.59	1.23	1.06	788	21	47
-D03/HD	123471	pH_5.3_6.5_1532	0.54	1.07	0.98	788	21	47
-D03/HD	123714	pH_5.3_6.5_2178	0.56	1.02	0.87	686	22	57
-D03/HD	1751	pH_3_5.6_543	0.58	1.21	1.06	806	19	33
-D03/HD	21836	pH_3_5.6_741	0.74	1.16	0.65	268	7	11
-D03/HD	22197	pH_5.3_6.5_1335	0.86	1.30	0.59	779	25	37
-D03/HD	2687	pH_5.3_6.5_935	0.44	1.50	1.77	1005	21	33
-D03/HD	43664	pH_5.3_6.5_1352	0.85	1.13	0.41	1079	33	40
-D03/HD	4537	pH_3_5.6_230	0.69	1.42	1.03	2245	57	39
-D03/HD	47020	pH_5.3_6.5_1901	0.57	1.56	1.45	159	4	16
-D03/HD	47829	pH_3_5.6_1107	0.49	1.27	1.38	654	21	40
-D03/HD	50249	pH_5.3_6.5_933	0.48	1.36	1.52	476	10	14
-D03/HD	50542	pH_5.3_6.5_1078	0.56	1.27	1.20	356	10	16
-D03/HD	51365	pH_3_5.6_353	0.63	1.57	1.32	497	9	13
-D03/HD	55362	pH_3_5.6_566	0.46	1.57	1.77	1295	32	32
-D03/HD	56920	pH_3_5.6_1074	0.71	1.14	0.70	591	24	27
-D03/HD	68036	pH_5.3_6.5_1275	0.39	1.68	2.10	616	14	29
-D03/HD	68304	pH_5.3_6.5_1334	0.76	1.66	1.12	402	9	19
-D03/HD	73818	pH_5.3_6.5_1434	0.52	1.27	1.29	481	13	23
-D03/HD	73903	pH_3_5.6_1210	0.74	2.09	1.51	89	2	10
-D03/HD	74278	pH_3_5.6_1058	0.37	1.46	1.96	460	10	25
-D03/HD	74449	pH_5.3_6.5_806	0.59	1.29	1.13	1149	27	31
-D03/HD	77481	pH_5.3_6.5_891	0.55	1.92	1.81	1141	28	30
-D03/HD	77495	pH_3_5.6_1305	0.86	1.48	0.78	533	21	39
-D03/HD	77747	pH_5.3_6.5_2104	0.35	1.36	1.98	330	5	20
-D03/HD	78661	pH_3_5.6_698	0.62	1.49	1.26	955	21	46
-D03/HD	81014	pH_5.3_6.5_388	0.98	1.91	0.96	1316	31	30
-D03/HD	82534	pH_5.3_6.5_1142	0.39	1.78	2.19	182	3	4
D03/HD	102382	pH_5.3_6.5_2087	1.40	0.63	-1.15	523	9	36
D03/HD	102581	pH_3_5.6_849 ng	1.44	0.96	-0.60	504	11	41
D03/HD	104797	pH_3_5.6_174	1.63	0.48	-1.74	490	11	14
D03/HD	105381	pH_5.3_6.5_2326	1.10	0.50	-1.15	303	9	31
D03/HD	108463	pH_5.3_6.5_1980	1.83	0.67	-1.47	215	6	29
D03/HD	111131	pH_5.3_6.5_1906	1.66	0.64	-1.40	568	16	40
D03/HD	119731	pH_3_5.6_675	1.30	0.68	-0.92	1505	113	56
D03/HD	120312	pH_3_5.6_793	1.41	0.54	-1.40	160	3	11
D03/HD	120312	pH_3_5.6_891	1.41	0.53	-1.43	161	3	12
D03/HD	121169	pH_5.3_6.5_2150	1.55	0.67	-1.22	103	2	8
D03/HD	122081	pH_3_5.6_701	1.55	0.44	-1.79	119	3	10
D03/HD	122230	pH_3_5.6_904 ng	1.26	0.76	-0.74	710	21	46
D03/HD	122415	pH_3_5.6_694	1.25	0.63	-1.00	1396	33	47
D03/HD	122415	pH_3_5.6_814	1.40	0.55	-1.32	335	6	14
D03/HD	122696	pH_3_5.6_129	1.71	0.53	-1.69	385	6	13
D03/HD	122920	pH_3_5.6_533	1.19	0.66	-0.84	235	5	7
D03/HD	122920	pH_3_5.6_545	2.13	1.19	-0.84		3	4
D03/HD	123668	pH_3_5.6_1317	1.45	0.77	-0.92	766	40	77
D03/HD	123989	pH_3_5.6_709	1.34	0.50	-1.40	104	3	6
D03/HD	22004	pH_5.3_6.5_1913	1.86	0.37	-2.32	1087	25	60
D03/HD	23090	pH_3_5.6_1245	1.44	0.66	-1.12	335	9	38
D03/HD	49081	pH_3_5.6_266	1.25	0.73	-0.79	691	23	21
D03/HD	49081	pH_3_5.6_267	1.35	0.48	-1.51	422	12	13
D03/HD	51103	pH_5.3_6.5_997	1.39	0.64	-1.12	892	20	32
D03/HD	5614	pH_3_5.6_876	1.36	0.58	-1.22	425	7	21
D03/HD	56840	pH_5.3_6.5_1845	1.35	0.65	-1.06	1324	36	58
D03/HD	64561	pH_3_5.6_1430 ng	1.40	0.84	-0.74	83	4	16
D03/HD	66647	pH_5.3_6.5_2114	1.24	0.55	-1.18	332	6	29
D03/HD	68608	pH_5.3_6.5_1806	1.73	0.46	-1.89	757	49	56
D03/HD	68608	pH_5.3_6.5_1809	1.73	0.77	-1.18	497	27	57
D03/HD	68608	pH_5.3_6.5_1812	1.32	0.57	-1.18	642	79	50
D03/HD	71092	pH_5.3_6.5_1371	1.54	0.74	-1.06	721	28	58
D03/HD	71326	pH_5.3_6.5_1920	1.83	0.48	-1.94	593	16	15
D03/HD	72567	pH_3_5.6_947	1.30	0.64	-1.03	437	16	13
D03/HD	74118	pH_3_5.6_969	1.20	0.63	-0.92	1287	66	62
D03/HD	74198	pH_5.3_6.5_731	1.48	0.54	-1.47	832	18	16
D03/HD	74198	pH_5.3_6.5_745	1.79	0.28	-2.64	822	21	19
D03/HD	75294	pH_3_5.6_908 ng	1.18	0.79	-0.60	334	12	29
D03/HD	76672	pH_5.3_6.5_914	1.64	0.60	-1.47	1188	25	33
D03/HD	78968	pH_3_5.6_1349	1.89	0.33	-2.47	175	3	22
D03/HD	79373	pH_5.3_6.5_2241	1.36	0.48	-1.51	117	4	16
D03/HD	79686	pH_5.3_6.5_816	1.43	0.60	-1.25	825	16	22
D03/HD	79686	pH_5.3_6.5_817	1.88	0.60	-1.64	1903	53	45
D03/HD	79686	pH_5.3_6.5_822	1.51	0.64	-1.22	2019	69	47
D03/HD	80003	pH_5.3_6.5_1262	1.56	0.62	-1.32	751	14	28
D03/HD	v1.2_46348	pH_3_5.6_565 sg	1.22	0.78	-0.64	721	19	36
